# A Dynamic Clamp on Every Rig

**DOI:** 10.1523/ENEURO.0250-17.2017

**Published:** 2017-10-23

**Authors:** Niraj S. Desai, Richard Gray, Daniel Johnston

**Affiliations:** Center for Learning and Memory and Department of Neuroscience, The University of Texas at Austin, Austin, TX 78712

**Keywords:** Dynamic clamp, electrophysiology, patch clamp

## Abstract

The dynamic clamp should be a standard part of every cellular electrophysiologist’s toolbox. That it is not, even 25 years after its introduction, comes down to three issues: money, the disruption that adding dynamic clamp to an existing electrophysiology rig entails, and the technical prowess required of experimenters. These have been valid and limiting issues in the past, but no longer. Technological advances associated with the so-called maker movement render them moot. We demonstrate this by implementing a fast (∼100 kHz) dynamic clamp system using an inexpensive microcontroller (Teensy 3.6). The overall cost of the system is less than USD$100, and assembling it requires no prior electronics experience. Modifying it—for example, to add Hodgkin–Huxley-style conductances—requires no prior programming experience. The system works together with existing electrophysiology data acquisition systems (for Macintosh, Windows, and Linux); it does not attempt to supplant them. Moreover, the process of assembling, modifying, and using the system constitutes a useful pedagogical exercise for students and researchers with no background but an interest in electronics and programming. We demonstrate the system’s utility by implementing conductances as fast as a transient sodium conductance and as complex as the Ornstein–Uhlenbeck conductances of the “point conductance” model of synaptic background activity.

## Significance Statement

We describe a system for adding dynamic clamp capability to any existing intracellular electrophysiology rig. Built around a simple microcontroller, the addition is inexpensive (<USD$100), can be used in parallel with existing data acquisition systems (and hence entails no disruption of existing experiments), and does not require any technical experience that a typical neuroscientist is unlikely to possess. Its performance is comparable in speed and accuracy to the leading alternatives. This system should make the dynamic clamp method accessible to a wide range of cellular electrophysiologists.

## Introduction

Current clamp and voltage clamp are the standard configurations of cellular electrophysiology. Every trained electrophysiologist is familiar with their properties, and every standard electrophysiological system incorporates them. And yet there is a third extant and potentially useful configuration: dynamic clamp (a.k.a., conductance clamp; Prinz et al., 2004; Destexhe and Bal, 2009; Prinz and Cudmore, 2011). Introduced independently and concurrently by two different groups (Sharp et al., 1992, 1993; Robinson and Kawai, 1993), dynamic clamp is grounded in the idea that the effects that voltage-gated and ligand-gated channels have on a neuron’s membrane potential can best be understood as changes in conductance rather than in current. The shift in emphasis requires that the electrophysiological system monitor membrane potential and use it, in real time, to calculate what current simulated channels would have passed had they been physically present.

The idea of dynamic clamp is simple, but how to implement it is not. The stumbling block is that dynamic clamp calculations must be done in real time, which is to say faster than any meaningful changes in channel properties or in membrane potential. In mammalian cortex, this means (much) faster than 10 kHz. A wide variety of implementations have been proposed since the earliest years, using technically sophisticated manipulations of hardware and software (Dorval et al., 2001; Pinto et al., 2001; Kullmann et al., 2004; Raikov et al., 2004; Desai and Walcott, 2006; Nowotny et al., 2006; Olypher et al., 2006; Milescu et al., 2008; Kemenes et al., 2011; Clausen et al., 2013; Ortega et al., 2014; Biró and Giugliano, 2015; Yang et al., 2015). These efforts have been useful and have had a broad impact, but they have not established dynamic clamp as a part of the standard repertoire of contemporary cellular electrophysiology. The limiting issues have been cost (as much as USD$6000, in one case), the requirement in some cases that existing data acquisition systems be replaced or substantially modified rather than merely supplemented, and the technical demands required of users in some cases (e.g., use of a digital signal processing board or facility with C++ or real-time Linux).

These limiting issues have all been valid heretofore, but we would argue that they are no longer. The present decade has witnessed an explosion of projects designed to enable nonengineers and other nonspecialists interested in building tools and other useful objects to make use of modern technological advances (Anderson 2014; Finley 2014; Morozov 2014). The Arduino microcontroller, in the field of embedded electronics, is perhaps the premier example (https://www.arduino.cc). Collectively called the “maker movement,” these projects have already had a substantial impact in multiple areas of modern neuroscience, including multielectrode electrophysiology, imaging, behavioral neuroscience, and automated patch clamping (Teikari et al., 2012; Potter et al., 2014; Baden et al., 2015; Desai et al., 2015; Siegle et al., 2017).

In this article, we demonstrate that one product of the maker movement, a microcontroller called the Teensy 3.6 (https://www.pjrc.com), can be used to add dynamic clamp capability to any intracellular electrophysiology rig (whether for patch or sharp electrodes). The addition is cheap (less than USD$100), can be used in parallel with existing data acquisition systems (and hence entails no disruption of existing experiments), and does not require any technical experience that a typical neuroscience graduate student is unlikely to possess. Indeed, the process of building and using the system will likely teach novices and even moderately experienced researchers some useful things about electronics and programming, as well as initiating them into the potential of other maker movement projects. We demonstrate that the system can handle conductances as fast as a transient Hodgkin–Huxley-style sodium conductance and as complex as the Ornstein–Uhlenbeck conductances of the “point conductance” model of synaptic background activity (Destexhe et al., 2001).

## Methods

**Figure 1.**
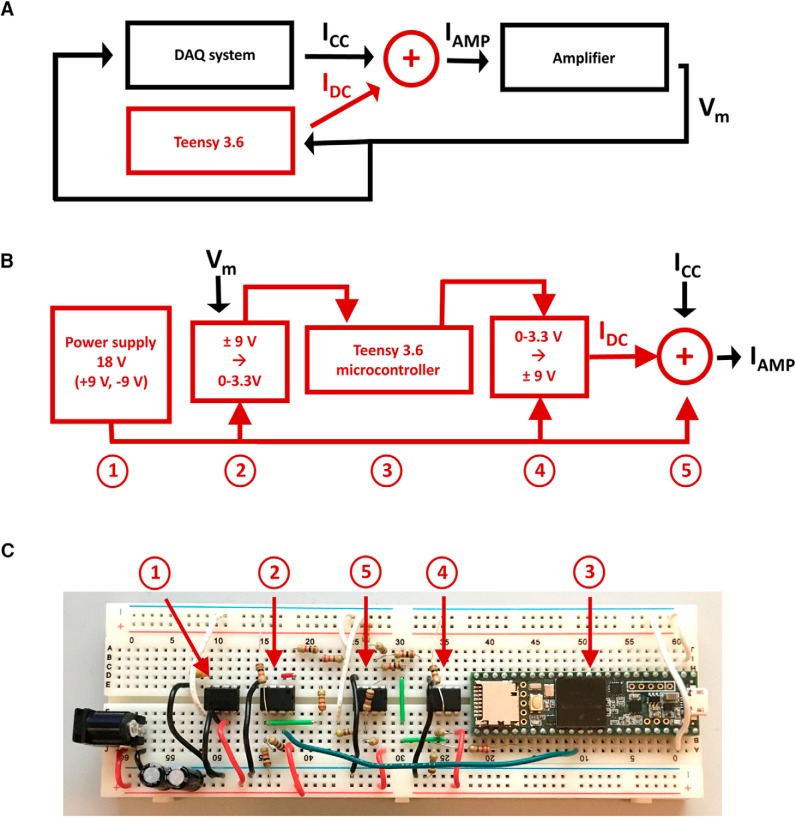
Schematic representations of the system. ***A***, The Teensy 3.6 microcontroller and its associated electronics (red) are added to an existing system (black) consisting of an intracellular amplifier and a data acquisition (DAQ) system. The amplifier sends the membrane potential *V_m_* to both the DAQ system and the Teensy system. The DAQ system, which could (for example) be comprised of a Digidata 1500 and pClamp 10 software, records *V_m_* to disk as usual and specifies whatever current (I_CC_) it would inject in a standard current-clamp configuration. The microcontroller uses *V_m_* to calculate what current (I_DC_) the dynamic clamp conductances would have passed had they been physically present. The sum of these two currents, I_AMP_ = I_CC_ + I_DC_, is sent to the command input of the amplifier to be injected into the neuron. ***B***, The Teensy system consists of five parts: (1) a power supply 18 V, which is broken up into a positive voltage (9 V) and a negative voltage (–9 V) to power the other circuits and to provide both positive and negative rails; (2) a differential amplifier circuit that maps the output of the intracellular amplifier, which will be in the range ±9 V, onto the range 0–3.3 V that the Teensy can read; (3) the Teensy controller itself; (4) a second differential amplifier circuit that maps the output of the Teensy, which will be in the range 0–3.3 V, onto the range ±9 V the intracellular amplifier expects at its command input; and (5) a summing circuit that adds the voltage commands representing I_CC_ and I_DC_. A voltage representing the sum I_AMP_ is sent to the intracellular amplifier and thereafter injected into the neuron. ***C***, The entire system can be built on a standard solderless breadboard. The five parts of the system are indicated by the arrows. All of the components (resistors, capacitors, ICs, wires, and microcontroller) can be secured simply by pushing their wires into the breadboard holes; no soldering is required. A detailed, step-by-step description of how to assemble the five parts of the system on a breadboard is available in the Extended Data 1 (“Assembling the system on a solderless breadboard”).

A schematic overview of the system is given in Fig. 1*A*. The portions in black are present on every intracellular electrophysiology rig: an amplifier and a data acquisition (DAQ) board. The amplifier could be a Multiclamp 700B, a Dagan BVC-700A, a HEKA EPC-10, an AM Systems 2400, or any other amplifier that monitors a neuron’s membrane potential and injects current into that neuron through a patch or sharp electrode. The DAQ board could be a Molecular Devices Digidata 1500, a HEKA ITC-18, a National Instruments PCIe-6343, or any of a huge number of other boards that work with Macintosh, Windows, or Linux operating systems; our dynamic clamp system places no constraint in this regard. We assume that the DAQ board is controlled on the host computer by a DAQ system suitable for intracellular electrophysiology. The system could be one of the commercial systems on the market (e.g., Molecular Devices pClamp 10 or AxoGraph), or it could be open source (e.g., Janelia’s Wavesurfer) or homemade. The point is simply that the electrophysiology rig already includes the components necessary to patch or impale neurons and record current clamp data.

Into this existing, working configuration, we insert the portion of Fig. 1*A* depicted in red. It consists of a Teensy 3.6 microcontroller and associated electronics. We chose the microcontroller because, compared with other devices of its class and in its price range, it is fast (180 MHz clock speed), has substantial memory (256 kB RAM), and has a floating point unit (more on this in Discussion). The Teensy is responsible in our system for performing all the dynamic clamp calculations. It determines, moment by moment, what current a voltage- or ligand-gated conductance would pass were it physically present and adds this to the current that the existing DAQ system has been instructed to inject (e.g., a family of current steps). That is, the existing DAQ system continues to perform all the standard current clamp (or voltage clamp) functions. The Teensy system simply adds a dynamic clamp component: it adds the current from simulated conductances to the current that the existing current clamp system specifies.

For the Teensy to do this, some electronic additions are required (Fig. 1*B*): (1) a power supply, (2) circuitry to map the voltage output of the intracellular amplifier (typically ±9 V) representing the neuron’s membrane potential to the voltages the Teensy can read (0–3.3 V), (3) electrical connections to and from the Teensy, (4) circuitry to transform the Teensy’s output (0–3.3 V) into a voltage the intracellular amplifier can correctly interpret (typically ±9 V) as a current (in pA) to be injected into the neuron, and (5) circuitry to sum the dynamic clamp currents specified by the Teensy and the current clamp currents specified by the DAQ system. We explain these five additions, all of which can be built on a single solderless breadboard (Fig. 1*C*), in the five sections that follow.

After that, we discuss the software that controls the Teensy. Although the Teensy 3.6 is not a microcontroller of the Arduino line (www.arduino.cc), it is very similar and can be programmed using the (open-source) Arduino integrated development environment (IDE). We provide, in the online material, the code we used to program various simulated conductances; these serve as examples for users who might wish to program different simulated conductances. We also discuss code written in the (open-source) Processing language (www.processing.org) to change dynamic clamp parameters “on the fly” (i.e., during a recording). Processing is a useful language because it is very simple and because, like Arduino, its code can be used without modification on all three major operating systems (Macintosh, Windows, and Linux). We end the methods section by discussing how to calibrate the electronic components of the dynamic clamp system to ensure best performance.

Source code, a parts list, photographs, and step-by-step instructions are included in the Extended Data 1. Postpublication updates will be available at a website we have created for this purpose (dynamicclamp.com), with software archived at the public repository Github (https://github.com/nsdesai/dynamic_clamp)

10.1523/ENEURO.0250-17.2017.ed1 Extended Data 1 Parts list.Assembling the system on a solderless breadboard.Where to obtain and how to install Arduino and Processing.Using and modifying Arduino software (including our Arduino code).Using and modifying Processing software (including our Processing code).Adding a potassium M conductance.Matlab alternative to Processing.Calibration procedure.Alternatives to Teensy. Download Extended Data 1, ZIP file.

### Power supply

The power supply serves two purposes: it provides power for the operational amplifiers (“op-amps”) of the other circuits, and it provides the positive (negative) reference voltages the other circuits use to shift up (down) the voltages sent to (from) the Teensy microcontroller. For both these purposes, we require a positive voltage (approximately 9 V) and a negative voltage (approximately –9 V).

The simplest power source suitable for both purposes is an 18-V DC wall adaptor (colloquially called a “wall wart”). Such an adaptor typically terminates in a barrel plug that can be plugged into a barrel connector. (We provide a full parts list, including links to supplier webpages, in the Extended Data 1, so readers can see for themselves what all the parts look like.) This power source cannot work alone because it is positive only, whereas we wish to have both positive and negative voltages. More precisely, we wish to break up the 18 V into one rail at 9 V, one rail at –9 V, with a ground (called a “virtual ground”) right at the halfway point.

**Figure 2.**
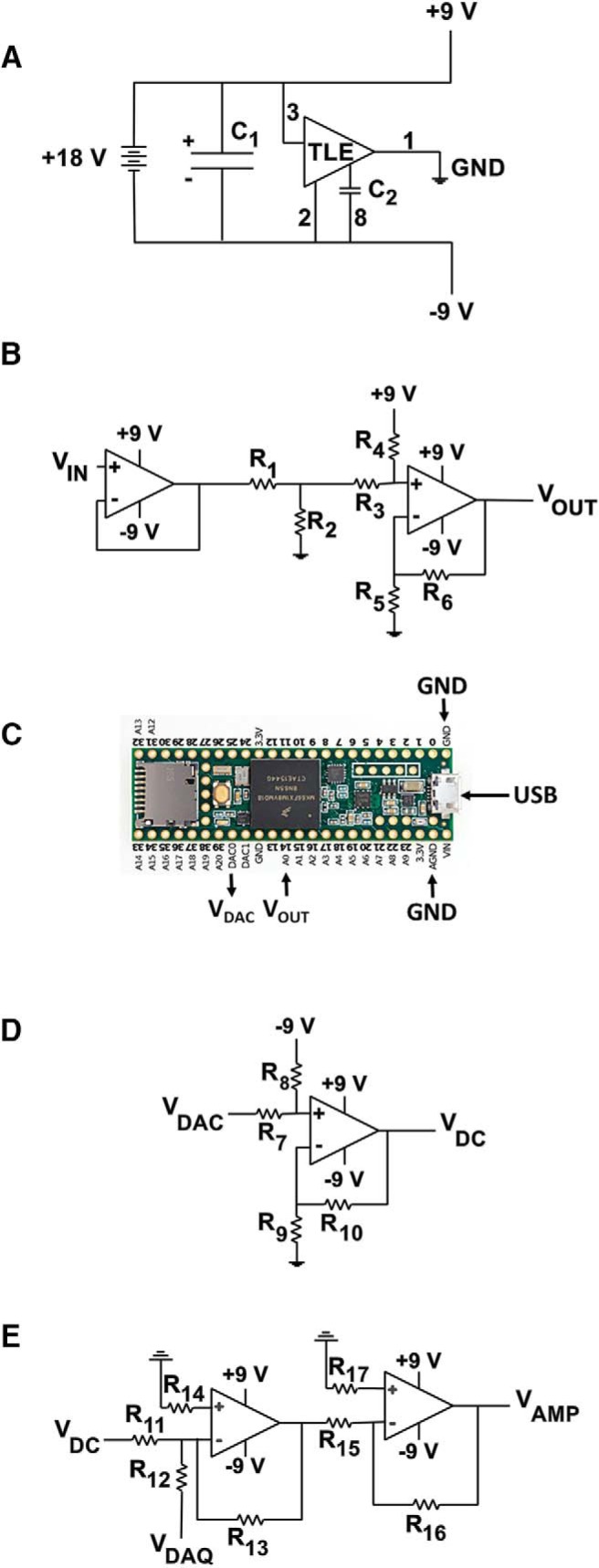
Breadboard electronics. The five parts of the system illustrated in Fig. 1*B* are shown schematically. ***A***, Rail splitter power supply. An 18-V power supply (wall adaptor) is split by a TLE 2426 rail splitter IC into 9 V, –9 V, and ground. The capactors are C_1_ = 200 μF and C_2_ = 1 μF. The dark numbers refer to the pins of the TLE 2426 IC. ***B***, Follower, voltage divider, and differential amplifier circuits to map the range −9 to 9 V onto the range 0 to 3.3 V. The resistor values are R_1_ = 2200 Ω, R_2_ = 470 Ω, R_3_ = 4700 Ω, R_4_ = 22,000 Ω, R_5_ = 10,000 Ω, and R_6_ = 100 Ω. ***C***, Connections to the Teensy 3.6 microcontroller. The output of the previous circuit is fed to pin A0, and the output of pin DAC0 is fed to the next circuit. ***D***, Differential amplifier circuit to transform the output of the microcontroller (0–3.3 V, representing the dynamic clamp current) into a range (±9 V) expected by the intracellular amplifier. The resistor values are R_7_ = 4700 Ω, R_8_ = 22,000 Ω, R_9_ = 10,000 Ω, and R_10_ = 10,000 Ω. ***E***, Summing amplifier. The voltage command from the DAQ board (representing the current clamp’s specified current) is added to the voltage command from the Teensy microcontroller. The sum is sent to the command input of the intracellular amplifier. Resistors R_11_, R_12_, R_13_, R_15_, and R_16_ are 10,000 Ω; resistor R_14_ is 3300 Ω; and R_17_ is 4700 Ω.

Circuits that perform this function are called “rail splitters.” In principle, a purely passive rail splitter circuit—essentially a voltage divider—would suffice here, but in this design we opted for an op-amp circuit, because it minimizes asymmetry between the positive and negative rails and buffers the power supply from the downstream circuits. (Keeping one part of the system from interfering with other parts—“buffering”—is a general principle of electronic design.) The op-amp circuit we chose is a ubiquitous integrated circuit (IC) from Texas Instruments (TLE2426). Fig. 2*A* shows schematically how the IC is connected.

In the Extended Data 1, we describe and show (with pictures) how the circuit looks when the parts are physically connected on a solderless breadboard. The breadboard has four power rails. One rail is marked 9 V and another is marked –9 V. The downstream circuits use these two rails to power their own op-amps and as reference points for 9 V and –9 V. A third rail on the breadboard is connected to the virtual ground of the rail splitter. Every other voltage in this system will be referenced to this ground rail.

### Amplifier output to microcontroller input

An intracellular electrophysiology amplifier in current clamp mode monitors a neuron’s membrane potential and outputs a signal representing this value. For real neurons, the membrane potential will be in a range no wider than –90 to 90 mV. The representative output will depend on the gain of the amplifier, but for the typical settings of commonly used amplifiers the corresponding range will be –9 to 9 V. This is too broad a range for the analog inputs of the Teensy microcontroller (or other controllers of this class), which are limited to 0–3.3 V.

To map ±9 V from the amplifier onto 0–3.3 V to the microcontroller, we employ three distinct elements (Fig. 2*B*). The first is a follower circuit. It takes an input (±9 V) and simply sends out an identical output (±9 V). The follower’s purpose is to separate—buffer—the input from the output, to keep them from interfering with each other. The second is a voltage divider that transforms the voltage from the amplifier (±9 V) onto a more limited range (approximately ±1.6 mV; the precise numbers depend on the precise resistor values chosen). The third is a differential amplifier that adds (approximately) 1.6 V to shift the output of the second element into the range 0–3.2 V, which roughly matches the dynamic range of the Teensy’s analog-to-digital (ADC) input.

One can calculate the relationship between the input (*V_IN_*) to this three-element circuit and its output (*V_OUT_*) by assuming that all the resistor values and power supply voltages are exact and that all the op-amps are ideal (Senturia and Wedlock, 1975), as follows:

$$V_{OUT}=\left(1+\frac{R_{6}}{R_{5}}\right)\left[\frac{R_{4}R_{2}}{\left(R_{3}+R_{4}\right)\left(R_{1}+R_{2}\right)}V_{IN}+\frac{R_{3}}{\left(R_{3}+R_{4}\right)}V_{+}\right].$$

In this equation, *V*_+_ is the voltage of the positive power rail (9 V). Note that the relationship between *V_IN_* and *V_OUT_* is linear. We constructed this circuit on a breadboard using resistor values between 100 Ω and 22 kΩ (specified in the figure caption) and an IC that contains two op-amps (LM358n). Testing the breadboard circuit, we found that the empirical relationship between *V_IN_* and *V_OUT_* was indeed strictly linear (see Calibration), but that the numerical values of the slope and intercept were somewhat different from what the exact equation would predict (by ∼2%). This discrepancy resulted from imperfections in the (inexpensive) electronic components we chose and the nonideal behavior of the op-amps of the LM358n chip.

Fortunately, the discrepancy can be corrected in software, without having to substitute better (and more expensive) electronic components. As explained in Calibration, this can be done by measuring *V_OUT_* values in response to a range of known *V_IN_* values. The numbers can be fitted to a straight line and the resulting slope and intercept used instead of the calculated slope and intercept.

### Microcontroller connections

The output of the three-element circuit (now 0–3.2 V) is fed to an ADC input on the Teensy microcontroller (Fig. 2*C*). The Teensy 3.6 has 25 ADC inputs; our default software simply selects the first of these (A0, pin 14), but any can be used. The Teensy analog ground should be connected to the virtual ground defined by the rail splitter circuit. The Teensy has two digital-to-analog (DAC) outputs; our default software uses the first of these (A21).

### Microcontroller output to amplifier input

The output of the Teensy DAC will be a voltage between 0 and 3.3 V, but most amplifiers in current-clamp mode expect command voltages between –9 and 9 V, with negative voltages representing hyperpolarizing current injections and positive voltages representing depolarizing current injections. Mapping 0–3.3 V onto the range ±9 V is the inverse of the problem we faced earlier, and its solution is similar but inverted. We use a differential amplifier both to shift 0–3.3 V down to the range ±1.65 V and to amplify the result (Fig. 2*D*).

Assuming perfect, ideal components, we can calculate the relationship between the input supplied by the Teensy (*V_DAC_*) and the output of the circuit (*V_DC_*, representing the dynamic clamp command signal; Senturia and Wedlock, 1975):

$$V_{DC}=\left(1+\frac{R_{10}}{R_{9}}\right)\left[\frac{R_{8}}{\left(R_{7}+R_{8}\right)}V_{DAC}+\frac{R_{7}}{\left(R_{7}+R_{8}\right)}V_{-}\right].$$

Here, *V*_−_ is the voltage of the negative power rail (–9 V). Again, the relationship between input (*V_DAC_*) and output (*V_DC_*) is linear. And again, when we constructed the circuit on a breadboard, we found that, although the empirical relationship between *V_DAC_* and *V_DC_* was linear, it was not strictly given by the calculated formula (off by ∼2%). This discrepancy too can be resolved in software (see Calibration).

### Summing circuit

The fifth and last electronic circuit is designed to sum the dynamic clamp command voltage *V_DC_* and the current clamp command voltage from the DAQ system *V_DAQ_*. Summing voltages is a common electronics task, and we perform it in a standard fashion: an inverting amplifier that sums the two voltages but switches their polarity, followed by a second inverting amplifier that switches the polarity back (Fig. 2*E*).

### Software

#### Arduino IDE

There are multiple ways of programming the Teensy 3.6, including simply using the C language, but the most sensible way to do so is through the Arduino IDE (https://www.arduino.cc/en/Main/Software). Arduino has emerged over the last 5 y as the microcontroller of choice of the maker movement, including nearly all the neuroscience-related projects (Potter et al., 2014; Baden et al., 2015; Desai et al., 2015; Siegle et al., 2017). The Arduino IDE and its associated language retain the essential syntax of C while making the process of interfacing with a microcontroller straightforward. Although Teensy is not part of the Arduino line of microcontrollers, a Teensy-specific add-on to the Arduino IDE exists and allows one to use the IDE and most of its libraries (https://www.pjrc.com/teensy/teensyduino.html). Detailed installation and use instructions are included in the Extended Data 1.

We wrote our dynamic clamp software using the Arduino IDE. The code is contained in the Extended Data 1 folder *dynamic_clamp* and the main file is called *dynamic_clamp.ino*. Opening the latter opens not only the main file but also its associated files, which appear in separate tabs. Each tabbed file contains the code for a specific conductance. The main file is structured in three parts: global variables, a setup function, and a loop function. The global variables are self-explanatory (variables needed by all and therefore accessible to all functions); the setup function is run once when the program is uploaded to the board and does things like initialize serial communication between the host computer and the Teensy board; the loop function is run at every time step: it calls each of the conductance-specific tabbed files to get the current specified by that conductance.

In the example software, we coded five separate conductances: (1) a simple shunt conductance, (2) a hyperpolarization-activated cyclic nucleotide–gated (HCN) conductance (Gasparini et al., 2004), (3) a fast, transient sodium conductance (Johnston and Wu, 1994), (4) an excitatory postsynaptic conductance (EPSC; Compte et al., 2000), and (5) “high conductance state” synaptic background conductances (Destexhe et al., 2001, 2003). The first is simple. The second and third are Hodgkin–Huxley conductances with one and four gates, respectively. The fourth is a synaptic conductance that is triggered by a transistor–transistor logic (TTL) pulse sent by the DAQ board to the Teensy microcontroller. The fifth is comprised of two conductances—one excitatory, one inhibitory—generated by Ornstein–Uhlenbeck (OU) processes. Our example code demonstrates how to numerically integrate the stochastic OU equations and how to generate the Gaussian random numbers the OU processes require (Marsaglia and Bray, 1964). Together, these five examples span the range of conductances users are likely to wish to use, and this code is meant to provide templates from which users can create other conductances. As a further aid, in the Extended Data 1, we also describe step-by-step how to add a potassium M conductance (Fransen et al., 2002).

#### Processing

When the Arduino program is uploaded to the Teensy microcontroller, all of the dynamic clamp conductances are initialized to zero. They can be changed to nonzero values while the program is running. That is, the dynamic clamp conductances can be changed on the fly during a given recording.

The simplest way for the host computer to tell the microcontroller to modify the dynamic clamp conductances is through the USB port that connects them. In our default Arduino software, the microcontroller constantly checks for a serial communication from the host computer and changes the conductance values as soon as it arrives. Unfortunately, the Arduino IDE itself has no good way of sending real-time communication from the host computer to the microcontroller. Fortunately, many other programs do. One called Processing (www.processing.org) is especially well suited for this purpose: it is an open-source environment with a simple syntax (based on Java and thus possessing a family resemblance to the C-like Arduino) that has a huge user base and is platform independent (Windows, Macintosh, or Linux).

In the Extended Data 1, we include a Processing sketch (called *processing_control.pde*) that creates a graphical user interface (GUI) through which users can change the values of the maximal conductances (in nanoSiemens) of the five conductances of our default software. The GUI also allows users to modify the diffusion constants (in square nanoSiemens per millisecond) of the OU processes. All the parameters can be modified by moving the sliders in the GUI and pressing the “upload” button.

But there is nothing unique about Processing. Users are free to use any software they wish in order, for example, to couple their data acquisition and dynamic clamp software more tightly. To emphasize this point, we also include Matlab software (Windows, Macintosh, or Linux) in the Extended Data 1 that does the same things as the Processing sketch.

### Calibration

Electronic components are typically specified with some margin of error (e.g., the specified resistance or capacitance will be good only to within 1%) and no op-amp or other active component behaves ideally. This means that the slope and intercept values for the electronic circuits calculated above will be not quite correct. Empirically, using the components specified in the parts list, we find that “not quite correct” means “not good enough.”

**Figure 3.**
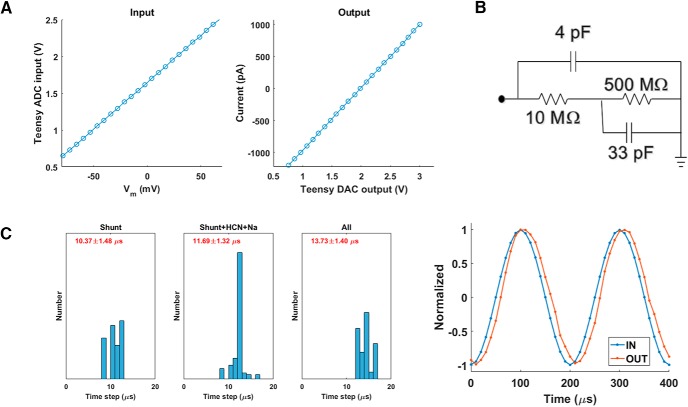
Calibrating and testing the system. ***A***, Although the electronic components were not ideal, the input-output characteristics of the system were highly linear. Left, the voltages measured by the microcontroller’s analog input in response to a range of membrane potentials. Right, the currents injected into a model cell in response to a range of voltages sent out by the microcontroller’s analog output. ***B***, To test the system, a model cell was attached to the intracellular amplifier’s headstage. Shown is the model’s equivalent circuit. ***C***, The system’s speed approaches 100 kHz and depends only weakly on the number of conductances being simulated. We recorded the durations of 51,200 time steps (at microsecond resolution) for each of three dynamic clamp configurations: shunt conductance only; shunt, HCN, and sodium conductances together; and shunt, HCN, sodium, Ornstein–Uhlenbeck, and EPSC conductances all together. Shown in the three figures at left are the resulting histograms; the number at the top of each is mean ± SD. The temporal jitter in all cases was 1–2 μs. To check the temporal latency, sinusoidal voltages (5 kHz) were fed to the system’s input (replacing *V_IN_* of Fig. 2*B*) and the resulting outgoing current commands (*V_AMP_* of Fig. 2*E*) were measured for a shunt conductance (2 nS). Both the input signal and the output signal were sampled at 100 kHz. The latency between input and output was roughly 10 μs.

Fortunately, the input-output functions of the circuits in question (Fig. 2*B*,*D*) are strictly linear (Fig. 3*A*). We determined this by directly measuring the output of the two circuits when they were subjected to a range of input voltages. In principle, users could do the same thing we did in that figure: use the DAQ board to send a voltage directly to the circuit input (Fig. 3*A*, left) or to measure the circuit output directly (Fig. 3*A*, right). But a slightly simpler method exists: attach a model cell to the headstage of the amplifier, measure the responses to a variety of current/voltage commands, and use these together with the model cell’s known architecture (arrangement of resistances and capacitances) to calculate the calibration parameters. The model cell could be as simple as a single resistor, but in Fig. 3*B* we used a Patch-1U model cell (“cell mode”) attached to a Multiclamp 700B amplifier (Molecular Devices). In cell mode, this model cell incorporates two resistors, representing input resistance and series resistance, and two capacitors, representing membrane capacitance and stray capacitance (due to the glass electrode; Fig. 3*B*).

In the Extended Data 1, we include a Processing sketch and step-by-step instructions for calibrating the system using a model cell attached to an amplifier.

### Testing

We tested the dynamic clamp system mainly using a Windows 10 computer, Multiclamp 700B amplifier (Molecular Devices), PCIe-6343 data acquisition board (National Instruments), Patch-1U model cell (Molecular Devices), and custom Matlab (The Mathworks) data acquisition software. To check that the system was indeed independent of operating system and equipment type, we also tested it on a rig with a Mac OS X computer, an Axopatch 200B amplifier (Molecular Devices), an ITC-18 data acquisition board (HEKA Instruments), and custom Igor Pro (Wavemetrics) data acquisition software.

Where indicated, we replaced the model cell in our tests with whole-cell patch-clamp recordings from mouse layer 2/3 or 5 pyramidal neurons. The mice were C57BL/6 males (6–8 wks old, Jackson Laboratory). Brain slices containing medial prefrontal cortex were prepared using standard procedures. Shortly after receiving a lethal dose of ketamine/xylazine, mice were perfused transcardially with an ice-cold solution containing (in mm): 2.5 KCl, 1.25 NaH_2_PO_4_, 25 NaHCO_3_, 0.5 CaCl_2_, 7 MgCl_2_, 7 dextrose, 205 sucrose, 1.3 ascorbate, and 3 sodium pyruvate (bubbled with 95% O_2_/5% CO_2_ to maintain pH at ∼7.4). Brains were removed, and a vibratome was used to make 300-µm-thick coronal sections. Slices were cut in the same ice-cold saline used for perfusion and then were held for 30 min at 35°C in a chamber filled with artificial CSF containing (in mm): 125 NaCl, 2.5 KCl, 1.25 NaH_2_PO_4_, 25 NaHCO_3_, 2 CaCl_2_, 2 MgCl_2_, 25 dextrose, 1.3 ascorbate, and 3 sodium pyruvate (bubbled with 95% O_2_/5% CO_2_). Thereafter, they were maintained at room temperature in the same solution. Patch-clamp recordings were obtained under visual guidance at 35°C using patch electrodes (3–7 MΩ) filled with an internal solution containing (in mm): 125 K-gluconate, 10 KCl, 4 NaCl, 10 HEPES, 4 Mg-ATP, 0.3 Tris-GTP, and 7 phosphocreatine (pH 7.4 at physiologic temperatures). All animal procedures were approved by the Institutional Animal Care and Use Committee of the University of Texas at Austin and were in accordance with National Institutes of Health guidelines.

### Software accessibility

The software for the system is available, together with other useful materials (a parts list, assembly and use instructions, photographs), as Extended Data 1. These are also available at a website we have created for this purpose (dynamicclamp.com), with software archived by the public repository Github (https://github.com/nsdesai/dynamic_clamp). Any postpublication improvements to the hardware or software will be available at these sites.

## Results

We validated the dynamic clamp system in two distinct but complementary ways: (1) with a model cell (Fig. 3*B*) attached to the amplifier headstage, and (2) with whole-cell patch-clamp recordings from layer 2/3 or 5 pyramidal neurons in slices of mouse prefrontal cortex (6–8 wks old). The first was our principal method because it provided a steady baseline against which the effects of added (simulated) conductance could be reliably contrasted. The second was useful because it more closely matched the experimental configuration in which potential users of this system are likely to be interested.

### Timing

One question is important in both cases: how fast is the dynamic clamp? For a single simulated conductance, the answer approaches 100 kHz, with a jitter of <2 μs. We determined this by measuring the distribution of time steps for different dynamic clamp configurations (Fig. 3*C*, three leftmost panels). Moreover, the time per cycle does not grow linearly with the number of simulated conductances, because most of the time cost (analog read + analog write) is fixed and common for all conductances. So, for example, simulating shunt, HCN, and sodium conductances at the same time can be done at better than 80 kHz (Fig. 3*C*, second from left). In keeping with these numbers, the latency between membrane potential *V_m_* and the dynamic clamp current was ∼10 μs (Fig. 3*C*, right).

In principle, the system could be sped up by using less averaging when reading from an analog input or using higher-quality op-amps (with a larger bandwidth and slew rate). However, we found—and show in what follows—that the system as described and constructed can handle even the most challenging conductance (i.e., transient sodium) without difficulty. We discuss the potential speed improvements in Discussion.

### Model cell: shunt conductance

**Figure 4.**
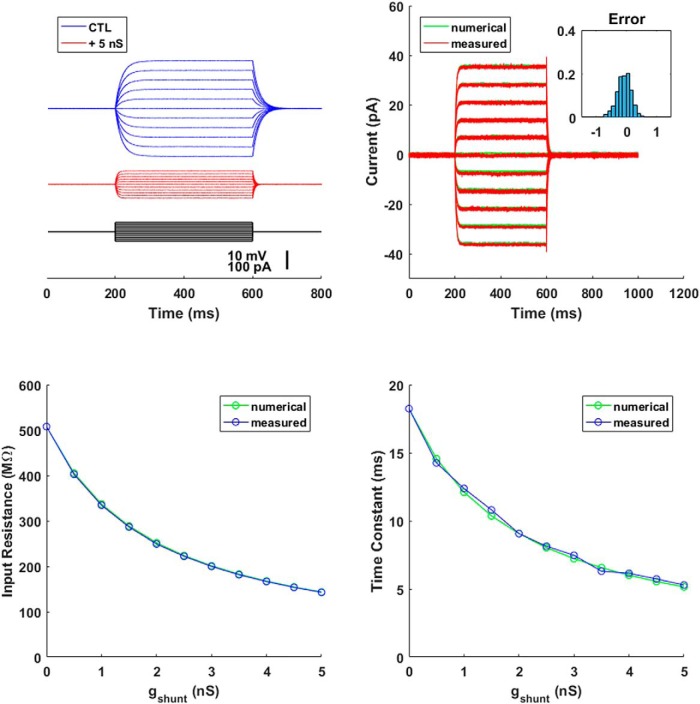
Shunt conductance. Adding 5 nS of shunt conductance to the model cell reduced and quickened the voltage deflections to a family of current steps (upper left). The shunt currents added by the dynamic clamp system (measured directly by the DAQ board) closely matched what they should have been given the recorded membrane potentials (numerical; upper right). The inset shows a probability histogram of the difference (error) between the measured and numerical currents. Varying the amplitude of the shunt conductance affected the input resistance and time constant (measured) as expected given the numerical values of the model cell resistance and the shunt conductance amplitudes (numerical; lower panels).

The simplest intrinsic conductance a neuron might possess is a shunt conductance, which is a constant conductance. The current it passes is the product of its amplitude and the driving force (the difference between the membrane potential and the conductance’s reversal potential): $I=-g_{shunt}\left(V_{m}-E_{rev}\right)$. In Fig. 4, we added a shunt conductance to the model cell of Fig. 3*B*; we used the amplifier’s built-in bridge balance and capacitance compensation circuitry to minimize the effects of series resistance and pipette capacitance. From the diagram, the equivalent circuit, after balance and compensation, should have been 500 MΩ in parallel with 33 pF. However, the components used to make up the model cell are far from ideal (see the Molecular Devices page on the model cell’s precision: http://bit.ly/2qHavi1). By injecting a variety of time-varying currents, we found that the model cell’s parameters were better fitted by resistance and capacitance values of 507.7 MΩ and 35.9 pF. We used these numbers to check the precision of our dynamic clamp currents.

In Fig. 4, we injected a family of current steps (–50 to 50 pA) into the model cell. The model was simply a resistor in parallel with a capacitor (an RC circuit). We therefore expected and found that the voltage responses to the steps were exponential growth and decay. Adding 5 nS of shunt conductance preserved the basic shape of the responses—all the shunt conductance does is decrease the value of R—but it reduced the steady-state deflections and made the responses faster. To check the system’s behavior quantitatively, we used a National Instruments DAQ board (50 kHz, 16 bit) to directly measure the dynamic clamp current emitted by the system and to compare it to the current that should have been passed given the recorded membrane potential had the system worked perfectly. These numbers (Fig. 4, upper right, measured versus numerical) were in good agreement. The inset shows a histogram of the deviation (error) between the measured and numerical currents during the steps. The absolute value of the error averaged 0.2 ± 0.2 pA (mean ± SD). Moreover, when we varied the amplitude of the shunt conductance, the measured input resistances (lower left) and time constants (lower right, estimated by fitting single exponentials to the voltage responses to current steps) closely matched the correct values determined by numerical calculation. The average difference was <2%, and in no case was the difference >5%.

### Model cell: HCN conductance

**Figure 5.**
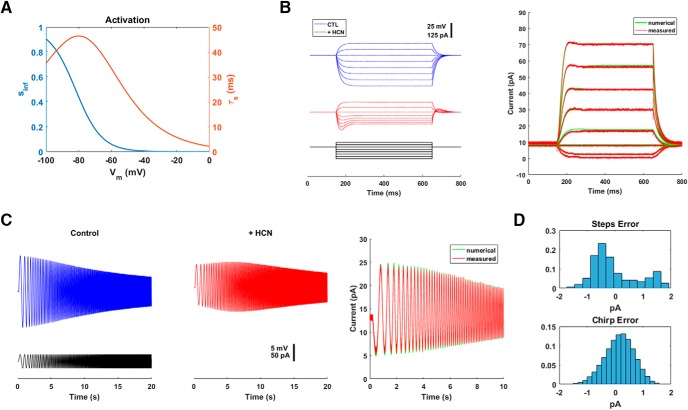
HCN conductance. ***A***, The conductance was modeled by a single-activation gate that had a steady-state value *s_inf_*(*V*) and a time constant τ*_s_*(*V*). ***B***, Current steps (–100 to 40 pA) were injected into a model cell without (CTL) and with (+HCN) the addition of 2 nS HCN conductance. Note that adding the simulated conductance resulted in the appearance of a sag potential (left). At right, the currents injected by the dynamic clamp system (measured, directly by the DAQ board) for the eight steps are plotted together with the result of numerically integrating the Hodgkin–Huxley equation using the fourth-order Runge–Kutta method (numerical). The top histogram (“steps error”) of ***D*** shows how good the agreement between the measured and predicted (numerical) currents was. ***C***, The model cell (Fig. 3*B*) is essentially an “RC circuit.” In response to a time-varying input, it acts like a low-pass filter. This can be seen (left) by its response to a chirp stimulus (black); the voltage deflection steadily decreases as the frequency increases. Addition of 4 nS HCN conductance transforms the system into a bandpass filter, with a resonant frequency. Again, the agreement between the current injected by the dynamic clamp system (measured) and the expected current given by numerical integration of the Hodgkin–Huxley equations (numerical) was excellent. ***D***, Histograms of the error between the measured and expected currents for step currents (top) and the chirp current (bottom).

One step up in complexity from a shunt conductance is an intrinsic conductance with a single activation gate. Several such conductances are important physiologically, including delayed-rectifier potassium and the HCN conductance. Here we focus on the HCN conductance. It activates but does not inactivate. What makes the conductance unusual is that the proportion of activated channels is increased by hyperpolarization (Fig. 5*A*) and that the reversal potential sits near the base of the activation curve. This configuration imbues the HCN conductance with interesting physiologic properties (Biel et al., 2009). The dynamics of the HCN conductance can be modeled by a single differential equation:

$$\frac{ds\left(t\right)}{dt}=-\left[s\left(t\right)-s_{inf}\left(V_{m}\right)\right]/\tau_{s}\left(V_{m}\right),$$

where s(t) represents the fraction of open HCN channels at any moment in time *t*, and $s_{inf}\left(V\right)$ and $\tau_{s}\left(V\right)$ are the voltage-dependent steady-state value and time constant, respectively (Fig. 5*A*; Gasparini et al., 2004). Our dynamic clamp system integrated this equation using the forward Euler method with a time step of ∼10 μs.

How well it did this is shown in Fig. 5*B*,*C*. Two important physiologic signatures of an HCN conductance are sag and resonance (Biel et al., 2009). Adding a simulated HCN conductance to the model cell introduced a sag potential (Fig. 5*B*) when the model was subjected to hyperpolarizing current steps. (The baseline potential of the model cell here and below was set at a resting potential of –70 mV.) The HCN conductance also effectively added an inductance to the circuit (termed a “phenomenological inductance” by Narayanan and Johnston, 2008). This transformed the equivalent circuit of the model cell from an RC circuit to an RLC circuit: it transformed the cell from a low-pass filter into a bandpass filter, with a distinct resonance frequency (Fig. 5*C*).

More important than these qualitative effects was that the simulated currents were quantitatively correct. We directly measured the currents emitted by the dynamic clamp system and compared them to the currents specified by precise numerical integration (fourth-order Runge–Kutta, 10-μs time step) of the differential equation. The agreement was very good (Fig. 5*B–D*); for both step currents and oscillating (chirp) currents, the average error (absolute value, mean ± SD) was <1 pA (steps: 0.7 ± 0.5 pA; oscillating: 0.4 ± 0.3 pA), and in no case was the absolute error ever >2 pA.

### Model cell: sodium conductance

The classic Hodgkin–Huxley formulation of the sodium conductance involves three identical and independent activation gates m(t) and a single inactivation gate h(t) (Johnston and Wu, 1994). The behavior of each is determined by a differential equation that involves voltage-dependent functions:

$$\frac{dm\left(t\right)}{dt}=-\left[m\left(t\right)-m_{inf}\left(V_{m}\right)\right]/\tau_{m}\left(V_{m}\right),$$

$$\frac{dh\left(t\right)}{dt}=-\left[h\left(t\right)-h_{inf}\left(V_{m}\right)\right]/\tau_{h}\left(V_{m}\right).$$

**Figure 6.**
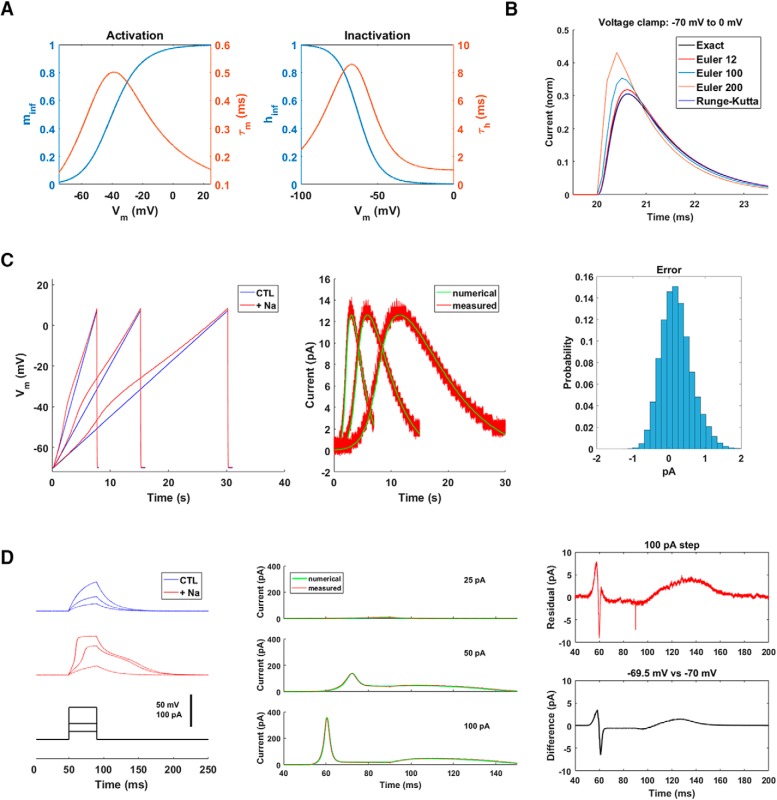
Sodium conductance. ***A***, The conductance was modeled using both an activation gate *m*(*V_m_*,*t*) and an inactivation gate *h*(*V_m_*,*t*). The steady-state and kinetic values of the two gates are plotted. The total sodium current was given by *g_Na_m* ^3^*h*(*V*_m_ – *E_Na_*), where *g_Na_* is the maximal sodium conductance and *E_Na_* is the sodium reversal potential (50 mV). ***B***, Comparison of different numerical integration methods. In a simulation, the voltage was stepped instantaneously from –70 mV to 0 mV at a time *t* = 20 ms. The resulting (inward) sodium current was calculated using the forward Euler method (time step 200, 100, or 12 μs), the fourth-order Runge–Kutta method (time step 10 μs), or an exact analytical calculation (exact). For the Euler simulation of 12 μs, the time step was also jittered by 2 μs (standard deviation). ***C***, At left, the response of the model cell to slow current ramps (5, 10, and 20 pA/s) is plotted in the absence (CTL) and presence (+ Na) of an added sodium conductance (*g_Na_* = 20 nS). In the middle, the close agreement between the sodium currents produced by the dynamic clamp system (measured) and those expected from precise numerical integration of the Hodgkin–Huxley equations (numerical) is demonstrated. At right is a histogram of the error (difference) between the measured and expected currents. ***D***, The model cell was subjected to brief current steps. Without a sodium conductance, the responses showed pure exponential growth, as expected of an RC circuit. With a sodium conductance (*g_Na_* = 80 nS), the responses showed nonlinear behavior above a threshold (>25 pA). The sodium currents (measured directly and expected from numerical integration) are plotted in the middle. Not only do the currents agree with each other, but they show a striking threshold behavior. At right (top), the error (difference) between the measured current and the expected current is plotted for the largest current step (100 pA). The shape of the error is consistent with what would be expected from a small offset in the baseline membrane potential. At right (bottom) is the difference between the fourth-order Runge–Kutta (time step 10 μs) estimate of the current for a baseline of –70 mV and the estimate for a baseline of –69.5 mV.

The voltage-dependent functions are shown in Fig. 6*A*. The sodium current is given by $I=-g_{Na}m{\left(t\right)}^{3}h\left(t\right)\left(V_{m}-E_{rev}\right)$, where $g_{Na}$ is the maximal sodium conductance and $E_{rev}$ = 50 mV is the sodium reversal potential. Integrating these equations in real time is more challenging than in the HCN case not only because there are now two equations rather than one, but also because the characteristic time associated with the activation gate $\tau_{m}\left(V\right)$ is very short (<0.5 ms). This sets the required time scale.

The microcontroller integrated these equations using the forward Euler method with a time step of <12 μs. To check that this was satisfactory, we simulated a voltage clamp experiment in which the model cell was stepped instantly from –70 to 0 mV. The sodium current elicited by such a step can be written down exactly. We compared the exact solution to those calculated using various numerical methods (Fig. 6*B*). As expected, the fourth-order Runge–Kutta method, which is widely used in modeling studies because of its stability and precision (Bettencourt et al., 2008), matched the exact solution almost perfectly. How well the forward Euler method did depended on the time step: time steps longer than 200 μs gave unstable solutions; those between 25 and 200 μs were stable but imprecise; but those <25 μs were generally satisfactory. In particular, forward Euler with a time step of 12 μs and a jitter of 2 μs, which matches the microcontroller’s performance, differed from the exact solution only near the peak of the sodium current and then only by a few percentage points (Fig. 6*B*, compare black and red traces). This result suggests that, with the possible exception of some fine details of spike shape (Bettencourt et al., 2008), forward Euler may be used to simulate Hodgkin–Huxley-style sodium conductances as long as the time step is <25 μs, which is the case here.

We tested the dynamic clamp system’s performance by using two types of stimuli (Fig. 6*C*,*D*): slow current ramps (5–20 pA/s) and brief current steps (0–100 pA, 40 ms). In response to the ramps, the simulated sodium current activated and inactivated as expected (Fig. 6*C*, left); the match between the measured currents and those predicted by a precise numerical integration (fourth-order Runge–Kutta, 10-μs time step) was very good—the absolute value of the error averaged 0.4 ± 0.3 pA (Fig. 6*C*, right). Even more striking was the response to brief current steps: these exhibited threshold behavior (Fig. 6*D*, left). Small currents (<25 pA) moved the model cell’s potential only a small distance from its baseline of –70 mV, thus eliciting minimal sodium current. But larger currents (>50 pA) resulted in sharp, spike-like bursts of sodium current. In the latter cases, the deviation between the measured current and the current expected from a precise numerical integration could be as large as 9 pA (Fig. 6*D*, right, top). This error was still small relative to the size of the underlying currents (>300 pA), and it likely originated from small imperfections in the patch clamp amplifier settings. Sodium conductance is very sensitive to such deviations. For example, if the baseline potential had been off by 0.5 mV because the model cell’s starting potential was not precisely –70 mV, that alone would have resulted in an error of the same shape and magnitude as the one we measured (Fig. 6*D*, right, bottom), without any contribution from the dynamic clamp system.

#### Model cell: EPSCs

Synaptic inputs to central neurons are mostly, although not exclusively, mediated by chemical synapses. These usually exhibit a rise time that is much faster than the decay time. There are several distinct ways of modeling the time course of synaptic currents, such as alpha functions and the difference of exponentials. Here we illustrate a particularly useful and general two-stage kinetic scheme (Destexhe et al., 1994; Compte et al., 2000; Walcott et al., 2011). We use it to model AMPA currents, but it is straightforward to modify it to model other types of synaptic currents. The kinetic scheme involves two variables:

$$\frac{dx\left(t\right)}{dt}=-\frac{x\left(t\right)}{\tau_{x}}+\underset{i}{\sum }\delta \left(t-t_{i}\right),$$

$$\frac{ds\left(t\right)}{dt}=-\frac{s\left(t\right)}{\tau_{s}}+\alpha_{s}x\left(t\right)\left[1-s\left(t\right)\right],$$

where α_*s*_ is a constant determining saturation properties; τ_*s*_ and τ_*x*_ are time constants controlling decay and rise times, respectively; δ(t) is the Dirac delta function; and $\left\{t_{i}\right\}$ are the presynaptic spike times. The resulting current is given by $I=-g_{syn}s\left(t\right)\left[V_{m}\left(t\right)-E_{rev}\right]$, where $E_{rev}$ is the synaptic reversal potential. To model AMPA currents, we set $E_{rev}=0\text{ mV}$, $\tau_{s}=10\text{ ms}$, $\tau_{x}=1\text{ ms}$, and $\alpha_{s}=1{\text{ ms}}^{-1}$.

**Figure 7.**
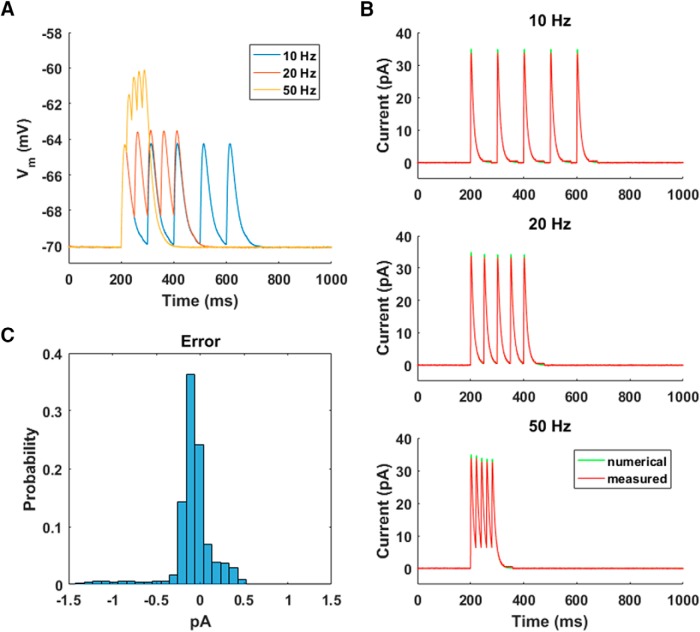
Excitatory postsynaptic currents. ***A***, EPSCs were triggered at fixed times with stimulation frequencies of 10, 20, and 50 Hz. Note how the potentials summate for the higher frequencies. ***B***, The agreement between the currents injected by the dynamic clamp system (measured) and the expected currents given by precise Runge–Kutta numerical integration of the two-stage kinetic scheme we used for EPSCs (Compte et al., 2000) was excellent at all frequencies. ***C***, Histogram of the difference (error) between the measured and expected currents. Data from all three frequencies were combined in this histogram.

The presynaptic spike times $\left\{t_{i}\right\}$ were determined by TTL triggers sent by the DAQ system. We sent triggers at rates of 10, 20, and 50 Hz (Fig. 7*A*). The higher frequencies showed evidence of synaptic summation. As was true of intrinsic conductances, the agreement between the measured dynamic clamp currents and numerical estimates of ideal behavior was excellent (Fig. 7*B*). This agreement can be quantified by considering a histogram of the errors, computed as the difference between the measured and expected currents, with the latter derived from a precise numerical integration of the differential equations (Fig. 7*C*). The average absolute error across all frequencies was 0.2 ± 0.2 pA (mean ± SD); the largest errors, which occurred at the peaks of the EPSCs, were <1.5 pA.

### Model cell: synaptic background activity

Most electrophysiological studies of neuronal properties have been conducted *in vitro*, using preparations such as brain slices and cell cultures, where the electrical and chemical background can be tightly controlled. However, neurons *in vivo* confront a rather different environment: they receive a continuous barrage of excitatory and inhibitory synaptic inputs, even in the absence of sensory or motor stimulation; a significant portion of total conductance is contributed by synaptic conductances rather than intrinsic ones; neurons are depolarized above rest by a much as 10 mV; and “resting” membrane potential fluctuates by as much as 5 mV (Steriade, 2001; Destexhe et al., 2003). A useful dynamic clamp tool to explore differences between *in vitro* and *in vivo* was contributed by Destexhe et al. (2001), who demonstrated that the electrical portion of synaptic background activity might be approximated by Ornstein–Uhlenbeck processes (Chance and Abbot, 2009).

**Figure 8.**
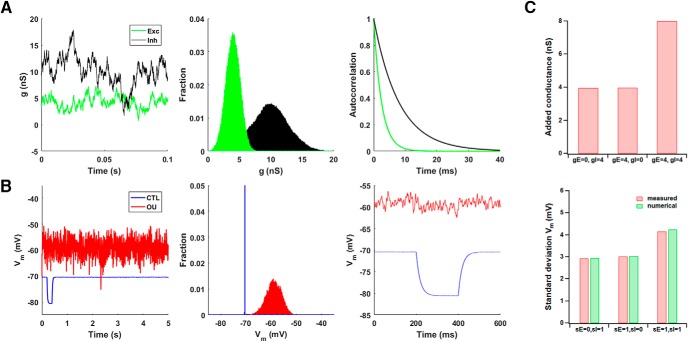
Synaptic background activity. ***A***, In the point conductance model of Destexhe et al., (2001), synaptic background activity is modeled by two noisy conductance trains. One represents excitatory input, and the other represents inhibitory input; both are generated by Ornstein–Uhlenbeck processes. Each train is normally distributed (middle) and is correlated at short times (right; the power spectrum goes like 1/*f* ^2^ for higher frequencies). ***B***, Without background activity, the model cell has a flat membrane potential (left) that is almost constant (middle); its input resistance is large (right). Adding *in vivo*–like background activity depolarizes the membrane, introduces membrane potential fluctuations, and reduces the input resistance. The vertical scale of the middle histogram is truncated so that the membrane potential distribution in the active state is easier to see. ***C***, Varying the mean (*g*, in nanoSiemens) and standard deviation (*s*, in nanoSiemens) of the excitatory and inhibitory conductances produced the predicted changes in total conductance (top) and membrane potential fluctuations (bottom). The added conductance is expected to equal the sum of *gE* and *gI*. The numerical estimates of $V_{m}$ standard deviation were calculated by simulating the model cell. When *sE* and *sI* were varied, *gE* and *gI* were held fixed at 4 nS.

Modeling synaptic background activity in this way, which has been called the “point conductance” method, requires integrating stochastic (rather than deterministic) differential equations and generating Gaussian random numbers. In our example code, we demonstrate how to do these things. Two conductance trains, one representing excitatory inputs and the other representing inhibitory inputs, were generated independently as OU processes. Each was determined by an equation of the form

$$\frac{dg\left(t\right)}{dt}=-\frac{1}{\tau }\left[g\left(t\right)-g_{0}\right]+\sqrt{D}\chi \left(t\right),$$

where *g*(*t*) is the value of the conductance, $g_{0}$ is its mean value, τ is a time constant, *D* is a diffusion constant, and χ(t) is a Gaussian white noise term of zero mean and unit standard deviation. As illustrated in Fig. 8*A* (left), such an equation produces a random walk in time around the mean value, with a variance given by σ^2^ = *D*τ/2.

Introduction of this synaptic background activity had three distinct effects on the model cell (Fig. 8*B*): a depolarization of 5–10 mV, membrane potential fluctuations of ∼10 mV, and a decrease in input resistance of more than a factor of 5. Varying the mean and standard deviation of the excitatory and/or inhibitory conductances produced changes in total conductance and membrane potential that closely matched what would be predicted given the model cell parameters (Fig. 8*C*).

### Pyramidal neurons

We further tested the system using whole-cell patch-clamp recordings from layer 2/3 and 5 pyramidal neurons in acute mouse prefrontal slices.

**Figure 9.**
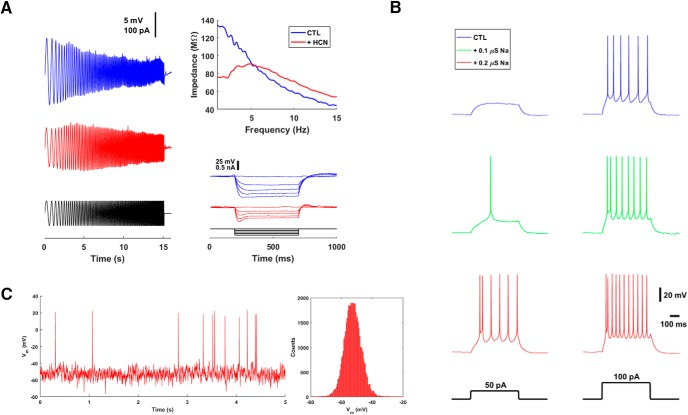
Dynamic clamp recordings from pyramidal neurons in slices of mouse prefrontal cortex. ***A***, HCN conductance. Left, a layer 2/3 pyramidal neuron shows a low pass filtering response (blue) when subjected to a chirp current (black). This is transformed into a bandpass response (red) when 6 nS HCN conductance is added by the dynamic clamp. Upper right, the impedance profiles show this effect quantitatively. Lower right, the neuron also developed a sag potential in response to hyperpolarizing current injections. ***B***, Sodium conductance. Addition of 100 or 200 nS of sodium conductance makes this layer 2/3 pyramidal neuron more excitable in a graded fashion. Shown are the responses to current steps of 50 and 100 pA. ***C***, Synaptic background activity in a layer 5 pyramidal neuron. The point conductance method was used to simulate an *in vivo*–like state (excitatory mean 3 nS, excitatory standard deviation 1.5 nS, inhibitory mean 6 nS, inhibitory standard deviation 3 nS). At left is a 5-s recording of membrane potential $V_{m}$. At right is a histogram of subthreshold membrane potential; the spikes of the 5-s recording were clipped out, and the remainder were used to construct the histogram.

Layer 2/3 pyramidal neurons have relatively little HCN current, at least when compared with pyramidal neurons in deeper layers (Biel et al., 2009). As a result, they respond to temporally fluctuating input as low-pass filters, and they exhibit no or very small sag potentials. To test the first property, we injected a chirp stimulus (sinusoidal current with a frequency that increases in time, also known as a ZAP stimulus) into a layer 2/3 pyramidal neuron. As expected, the voltage deflection dropped as the frequency increased (Fig. 9*A*, left). We quantified this effect by calculating the impedance, which can be thought of as a frequency-dependent resistance, by dividing the Fourier transforms of the voltage and the current (Fig. 9*A*, upper right). The impedance decreased monotonically between 0 and 15 Hz. Into this neuron, we then introduced an HCN conductance of 6 nS using the dynamic clamp system. With this addition, the voltage deflection in response to the chirp stimulus showed a peak (a resonance) when the stimulus frequency was near 5 Hz (Fig. 9*A*, left and upper right, traces in red). In the absence of the simulated HCN conductance, this neuron showed no sag potential, but addition of the HCN conductance both reduced the steady-state resistance (smaller voltage deflection in response to a fixed current) and added a sag potential (Fig. 9*A*, lower right).

To first order, the intrinsic excitability of neurons turns on the balance between hyperpolarizing currents (especially leak) and depolarizing currents (especially transient sodium). (To second and higher order, many other types of currents enter in.) We increased the intrinsic excitability of a layer 2/3 pyramidal neuron by injecting 100 or 200 nS of simulated sodium conductance (Fig. 9*B*). A current step that was subthreshold in the basal state (left, trace in blue) produced one or six action potentials (left, traces in green and red) when sodium conductance amplitude was increased. Likewise, a current step that was just suprathreshold in the basal state (right) produced more and more spikes as sodium conductance amplitude was increased. Moreover, the latency to first spike and the threshold of the first spike dropped as sodium conductance was added.

In an acute slice, neurons exist in a quiescent state with spontaneous firing rates and $V_{m}$ fluctuations near zero. This is very different from the active state that exists *in vivo* (Destexhe et al., 2003). We simulated an *in vivo*–like active state in a layer 5 pyramidal neuron in a brain slice using the point conductance method (Fig. 9*C*). The neuron’s activity reproduced the expected features of high conductance state background activity: a firing rate of 2 Hz, a mean depolarization of 10 mV, and $V_{m}$ fluctuations with a standard deviation of 5 mV.

#### Alternatives to Teensy

We built the dynamic clamp system around the Teensy 3.6 microcontroller because of its speed and memory, but the approach is more general. It is not limited to this particular device. In fact, many other microcontrollers could be used with only slight modifications to the code. To underline this point, in the online material (folder *Alternatives to Teensy*) we include software and instructions for using an Arduino Due or a chipKit uC32 in place of the Teensy 3.6. The first is a member of the Arduino line of microcontrollers (www.arduino.cc); the second is based on a separate family (called PIC) of microcontrollers (chipkit.net).

## Discussion

We have here introduced a dynamic clamp system built around a microcontroller and suitable for addition to any intracellular electrophysiology rig. In designing the system, we were guided by three requirements: (1) low cost (<USD$100), (2) compatibility with the wide range of hardware and software found on existing rigs, and (3) accessibility to researchers with little prior experience with electronics or programming. The system not only meets these three requirements, but its performance is also comparable in accuracy and speed to those posted by the leading alternatives (Destexhe and Bal, 2009; Prinz and Cudmore, 2011). It was able to simulate with an average error of only a few percentage points the same variety of conductances other systems have been used to simulate, and its single-conductance speed (90 kHz) was exceeded by only 3 of 24 published systems (pre-2012 systems reviewed by Lin [2012], Clausen et al. [2013], and Yang et al. [2015]).

The principal limitation on the accuracy of our system is that the ADC inputs and DAC outputs of the Teensy 3.6 microcontroller have 12-bit (4096-level) precision, rather than the 16-bit (65,536-level) precision available in recent DAQ systems. Although this is indeed a limitation, we would argue that it is not an important one. Consider the ADC input: 12-bit precision means that the input’s 3.3-V total range is sampled in increments of 0.8 mV. Given the empirical relationship between ADC input and membrane potential $V_{m}$ (Fig. 3*A*), we were therefore able to measure $V_{m}$ with a precision of 0.06 mV. For physiologically realistic experiments, this is almost certainly good enough. And it is a lower limit: as we note in the online material (“Assembling the system”), one can adjust the resistor values to take better advantage of the ADC input’s full dynamic range; our choices were conservative. An argument similar to the ADC one pertains to the DAC output.

A different potential limitation on accuracy—one that our system shares with most existing dynamic clamp systems (Destexhe and Bal, 2009)—arises from our use of forward Euler numerical integration for the Hodgkin–Huxley and Ornstein–Uhlenbeck equations (Bettencourt et al., 2008). However, our simulation of the transient sodium conductance (Fig. 6) suggests that this is not a determinative limitation, because the microcontroller can do the integration with a very fast time step (<12 μs), which is fast enough to obviate most problems. It is possible to implement a more precise numerical method on a Teensy-class microcontroller (see, e.g., http://bit.ly/2t6Jtyk), but this would come at the cost of simulation speed. For example, using the fourth-order Runge–Kutta method would drop the speed by a factor of two. There is a trade-off between accuracy and speed. Our data indicate that, even for transient sodium, forward Euler is satisfactory given how fast the Teensy microcontroller is.

The dynamic clamp system as a whole is fast because the microcontroller is doing nothing but implementing dynamic clamp routines. It is not maintaining an operating system, interacting with a user, updating a graphical interface, or implementing other unrelated routines. It is devoted exclusively to dynamic clamp. In this, it is similar to earlier hardware implementations, such as those based on digital signal processing boards (Desai and Walcott, 2006; Olypher et al., 2006), but with much reduced cost and complexity. Somewhat faster dynamic clamp systems have been introduced recently, including one based on Matlab’s xPC Target software (Clausen et al., 2013; >125 kHz) and another that uses Igor Pro software and National Instruments hardware (Yang et al., 2015; >100 kHz), but, for many potential users, these would require substantial modification of existing electrophysiology systems, as well as the purchase of new hardware or software.

In principle, the dynamic clamp system we describe could be made faster. On every cycle, the system spends the bulk of its time doing two things: reading from the ADC input and writing to the DAC output. One of our major goals was to keep the system as simple as possible, and so we used the built-in analog read and write functions of the Teensyduino version of the Arduino language. However, a defining feature of maker movement devices like the Teensy microcontroller—one that distinguishes them from many commercial devices—is that users have direct access to their inner workings; those innermost parts are not protected. One could reduce the time spent reading and writing the analog ports by addressing the registers directly and, for example, reducing how many samples the ADC input takes before reporting a result (see, e.g., https://github.com/pedvide/ADC). The cost of such a manipulation is in accuracy: it might increase noise. As always, there is trade-off between accuracy and speed. A smaller improvement might be obtained by using higher-quality electronic components. We chose the LM358 chip as an op-amp because of its ubiquity; it is available at nearly every hobbyist site. However, it has some limitations: it is not a “rail-to-rail” op-amp, which means that its behavior becomes erratic if the input voltages get close to the ±9 V rails of the power supply; it has a bandwidth and slew rate (how fast the voltage output can change in time) inferior to some other comparable op-amps (e.g., the OP484). Using these other op-amps would increase the cost of the system marginally (∼USD$20), but might be worthwhile depending on the user’s needs and would not degrade performance in any case.

Other microcontrollers (such as the Arduino Due, the top-of-the-line Arduino model) could be used in place of the Teensy 3.6 with only small modifications, but we chose the Teensy for three reasons. It has a clock speed equal to or faster than its competitors (180 versus 84 MHz for the Due), it has more memory (256 kB RAM versus 92 for the Due), and it has a floating point unit (which all of the Arduino models lack). The last point is the most important. Microcontrollers of the Arduino class tend to omit a floating point unit, a piece of hardware dedicated to and optimized for arithmetic on numbers where the number of digits after the decimal point might vary. (The opposite of a floating point number is called a fixed point number, which has a fixed number of digits after the decimal point. An integer is the simplest example.) Variables naturally represented by float point numbers are ubiquitous in neurophysiology and particularly in Hodgkin–Huxley calculations. One might attempt to transform floating point arithmetic to fixed point arithmetic for the sake of speed (instead of asking what 2.0 × 2.00 is, one might instead ask what 200 × 200 is and then divide the result by 10,000), but the Teensy’s architecture obviates the need for such machinations. Again, it keeps things simple.

## Synthesis

Reviewing Editor: Douglas Bayliss, University of Virginia School of Medicine Decisions are customarily a result of the Reviewing Editor and the peer reviewers coming together and discussing their recommendations until a consensus is reached. When revisions are invited, a fact-based synthesis statement explaining their decision and outlining what is needed to prepare a revision will be listed below. The following reviewer(s) agreed to reveal their identity: Astrid Prinz, Mark Beenhakker

This manuscript describes a new and affordable implementation of the dynamic clamp; both reviewers enthusiastically agree that it would be useful to share this “how-to” information with the electrophysiology community. That said, both reviewers raised issues regarding the imprecise nature of the language and qualitative comparisons to earlier implementations of the approach. Their specific comments and suggestions for improvement are provided below.

Reviewer #1

Major comments:

Throughout, the language of the manuscript sounds very colloquial (and thus often imprecise), more so than one would expect from a scientific publication. Furthermore, multiple statements about the performance of the described dynamic clamp system are qualitative ("very good", "meets the challenge", etc.) and would benefit from quantification - for example, what constitutes "very good"?

Statements about the rationales for testing the proposed dynamic clamp system with various conductance implementations, and their supposed relevance for neuroscience, are often unclear. For example, what exactly is meant by "one of the strongest positions in this debate" (L460 - L461)? What is described as a position is simply a collection of three parameter values - what system(s) are they supposed to apply to?

Minor comments:

L5: To be more general, I suggest replacing "patch clamp" with "electrophysiology". This applies throughout the manuscript, because the dynamic clamp is not limited to patch clamping, but is a general tool for other recording/simulation methods as well, such as sharp electrode experiments.

L11: Same suggestion.

L20: I suggest deleting "patch clamp".

L42: I find the description of the earliest dynamic clamp implementations as "just historical curiosities" somewhat disrespectful. At the time they were established, they were truly state-of-the-art, and describing them from the perspective of 25 years later is not doing them justice in terms of innovation.

L66: Looking at the cited website, I assume that it won't be very stable over time, as it describes the current product offerings of a commercial supplier. I leave it up to the eNeuro editors to decide whether this is appropriate for a scientific publication. The same concern applies to many other references in the manuscript, which are relevant to current product specifics, and will likely not last the test of time.

L166: Again, I find "patch clamp amplifier" not general enough. I will refrain from pointing this out again and again from now on, but it applies to the rest of the manuscript.

L186: The dimensions in the equation seem to be inconsistent. In the bracket, the first term is in units of voltage, whereas the second term is dimensionless (but should presumably also be in voltage units). That should be corrected.

L219: Same dimensionality issue.

L253: Spell out HCN on first use.

L718: Change "a patch clamp" to "an electronics".

L734: Delete one occurrence of "simply".

L746: Delete "patch clamp".

L749: Same.

Fig. 2: Compared to Fig. 1, the fonts and other components in Fig. 2 are much too tiny.

Reviewer #2

General Comments

The authors present a nice method to implement dynamic clamp in a cost-effective and relatively simple manner. Dynamic clamp is a powerful tool that enables the experimental electrophysiologist to artificially add (or subtract) membrane conductances into cells. The approach is now decades-old, but continues to provide fodder for new insights into cellular excitability. The primary advancement presented in the current study is the retooling of a powerful method such that virtually anybody can use it. I am enthusiastic about the possibility of bringing dynamic clamp to the masses (of patch clampers).

Specific Comments

Figure 1C: It may help future builders to label the components shown on the breadboard. The supplemental material is well-documented - nonetheless, adding labels to 1C might be helpful.

Line 343/Fig 3C: The authors present data showing that adding conductances adds a small increase in computing time (3us). Is there any concern that this increase comes at an experimental cost? Is it possible to empirically test (and quantify) how fast is fast enough? The authors point to the ability of the Teensy/forward Euler approach to produce transient sodium as evidence that their system is satisfactory. Does the qualitative comparison between numerical predictions and observed values provide the confidence? Perhaps plotting the comparison's residuals would be helpful.

Figure 8A Middle: Negative conductances? Is the X-axis on a log scale?

Figure 9C Right: Is there a description of this histogram in the text? There appears to be a brief description on lines 508-510, but it's a bit scant.

Line 516: The authors state that its "performance is comparable in speed and accuracy to the leading alternatives". How have the authors compared the performance of their system to that of other systems?

Minor Comments

Line 351: "speeded up" or "sped up"?

Line 459: A bit awkward. Perhaps "how much are neurons depolarized by synaptic..."
