# Supplementary material for: A Dynamic Clamp on Every Rig
Source: eNeuro. 2017 Oct 23;4(5):ENEURO.0250-17.2017. doi: 10.1523/ENEURO.0250-17.2017 (PMC5659377; doi:10.1523/ENEURO.0250-17.2017)
Supplement: Extended Data 1 [file enu005172417so1.zip › Extended_data/2_Assembling_the_system/Assembling the system.docx]

**Assembling the system**

**I. Before starting**

***Know how to use a breadboard***

You should know how to use a solderless breadboard. It is simple and very useful. If you haven’t used a breadboard before, there are many nice tutorials available online, including this one:

<https://www.sciencebuddies.org/science-fair-projects/references/how-to-use-a-breadboard>.

and this one:

<https://learn.sparkfun.com/tutorials/how-to-use-a-breadboard>.

***Get your tools together***

You will need three tools: a multimeter (to check resistances, the quality of connections, and voltages), a wire stripper (if you are using spools of insulated wire; if you are instead using the jumper wire kit mentioned in the parts list, then you don’t need this), and a voltage source (to check things are working properly as you build each part).

If you lack either of the first two tools, here are two suggestions:

Multimeter: <https://www.sparkfun.com/products/12966>

Wire stripper: <https://www.sparkfun.com/products/12630>

If you lack the third, you have a number of options, all of which are fine even though they differ in price and flexibility:

(1) Use a signal generator.

(2) Use the analog output of your data acquisition board.

(3) Use two AAA batteries and a battery holder (<http://bit.ly/2vRHPQV>). A single AAA battery has a voltage of 1.5 V; two of them together make 3.0 V. You will therefore be able to apply three voltages: +3 V, -3 V (by swapping the red and blue connections), and 0 V (by disconnecting the batteries and connecting input to ground directly with a wire). Obviously, having only three voltages isn’t as flexible as having a signal generator, but it’s sufficient to make sure everything is wired up correctly.

***Choose the resistor values***

In the manuscript, we gave resistor values suitable for our own patch clamp amplifier: Multiclamp 700B with a 500 MΩ feedback resistor and a current clamp gain of 10 (the latter two numbers are set in the Multiclamp Commander software). This amplifier with these settings amplifies the membrane potential $V_{m}$ by 100x at its scaled output (so that $V_{m}$ equal to -70 mV results in an output signal of -7.0 V) and uses a scaling of 400 pA/V to interpret signals at its command input (a change of -20 mV at the command input changes the injected current by -8 pA).

Other patch clamp amplifiers will have different gains/scalings. For example, an Axopatch 200B will have a gain of 10x from the output at its back panel (the output labeled “10$V_{m}$”) and a scaling of 2000 pA/V when the gain knob labeled β is at the default position of 1. A Dagan BVC-700 will have a gain of 100x (typically) but its current clamp scaling will be 200 pA/V when one uses a 0.01 headstage. Other combinations are possible for other amplifiers with other settings.

The resistor values of the breadboard circuit for a given amplifier should be such as to make good use of the full dynamic range of the Teensy 3.6 ADC input (0-3.3V) and DAC output (0-3.3V). The relevant equations are given on pp. 1-2 of the supplementary document *Calibration_procedure*. In the tables below, we indicate some good resistor choices to maximize use of dynamic range for different combinations of $V_{m}$ gain and current scaling. In choosing resistors for the ADC input (resistors 1-6), we were somewhat conservative. It is important never to feed the Teensy ADC input a negative voltage or one larger than +3.3 V because that can damage it, and so we avoided getting too close to the lower and upper limits. In choosing resistors for the DAC output (resistors 7-10), we aimed for a precision of 0.5-1.0 pA. We also chose resistor values that could be created easily using individual resistors found in Elegoo’s resistor kit (http://amzn.to/2vS6yVj); every resistor value can be created with at most two resistors in series. (We made the original board using Sparkfun’s resistor kit, but Elegoo’s kit is better. The wires are thicker and fit into the breadboard holes more snugly.)

**Resistor values for different ADC gains**


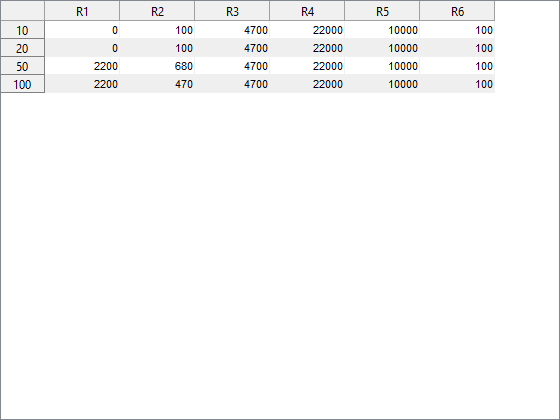


**Resistor values for different DAC scalings**


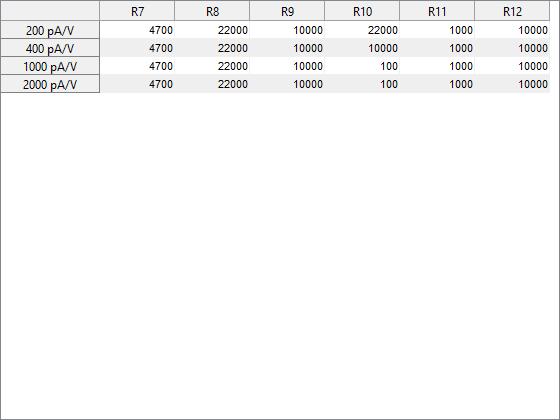


We will assemble the system compactly on a full-size breadboard, treating each of the five parts (main text Fig. 2) in turn.

**II. Power supply (Figure 2A)**

We begin with the power supply depicted in main text Fig. 2A. Power comes from a wall adapter connected through a barrel connector (Suppl. Fig. 1). (Higher-quality images of the breadboard are available in the subfolder *Higher_quality_images* within this folder.) It is very important that the wall adapter be a **regulated** one and not an unregulated one. A regulated adapter will maintain the same voltage output over a wide range of output current, whereas the voltage output of an unregulated adapter will vary with output current. The adapter we mention in the parts list is regulated; if you choose to use a different adapter, make sure that it is also regulated.

The adapter we use has a positive barrel and a negative center. The barrel connector has three pins: the one at the front is positive, the one at the back is negative, and the middle one can be ignored. You should insert the barrel connector into the breadboard as in Suppl. Fig. 1 and then use one wire to connect the front pin to the positive (red) power rail and the back pin to the negative (blue) power rail, as in the picture.

***Be careful!*** *Some wall adapters have the opposite polarity: the barrel is negative and the center is positive. If that is true of your adapter, you should reverse the connections to the power rails. In fact, to be careful, you should use your multimeter to measure the voltage difference between the red and blue rails when the wall adapter is plugged in. The multimeter probes most likely will not fit in the breadboard holes, but you can do this, as in the picture below, just by sticking wires with stripped ends into the board and pressing the probes against the ends.*


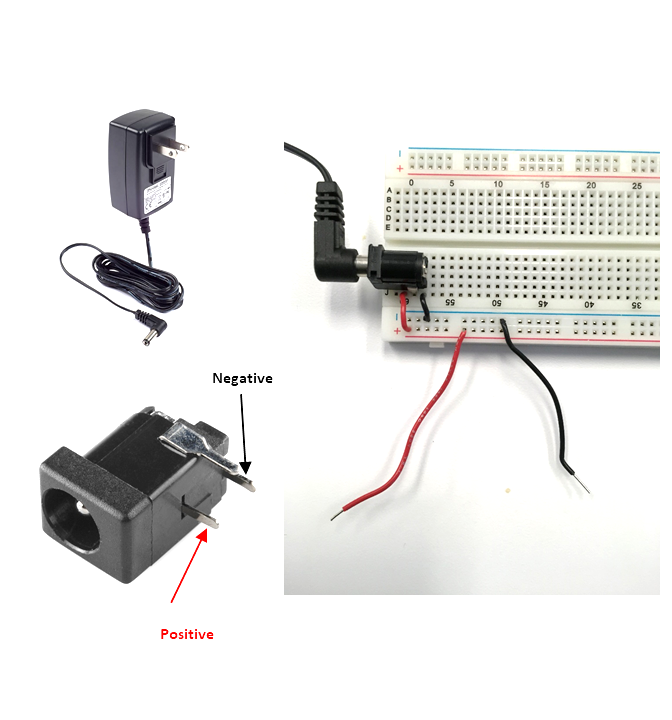


**Supplementary Figure 1.** Getting power. An 18 V wall adapter with a barrel plug end (upper left). A barrel connector with a positive pin and a negative pin (lower left). The connector on the breadboard (right). The small red wire connects the front pin to the red power rail; the small black wire connects the back pin to the blue power rail. You can check that the voltage polarity is correct by sticking wires into the rails as shown and measuring the voltage difference with a multimeter. Red should be +18 V compared to blue. If the sign is reversed, connect the front pin to the blue power rail and the back pin to the red power rail.

Unplug the wall adapter. **NEVER HAVE THE CIRCUIT CONNECTED TO AN ELECTRICAL OUTLET WHILE YOU’RE ASSEMBLING OR CHANGING IT.**

************************************************************************************

*AN ASIDE ABOUT BREADBOARDS*

*Some full-size breadboards, such as this one (*[*http://bit.ly/2iDXjqw*](http://bit.ly/2iDXjqw)*), have power rails that span their entire length, as indicated by unbroken blue and red lines. Others, such as this one (*[*http://bit.ly/2wjb9Sw*](http://bit.ly/2wjb9Sw)*), have breaks in the middle (Suppl. Fig. 2A). The break indicates that the rail on one side is not connected to the rail on the other. We want them to be connected. So, if you buy a breadboard like this, you should connect the two halves of all four rails with jumper wire (Suppl. Fig. 2B).*

*
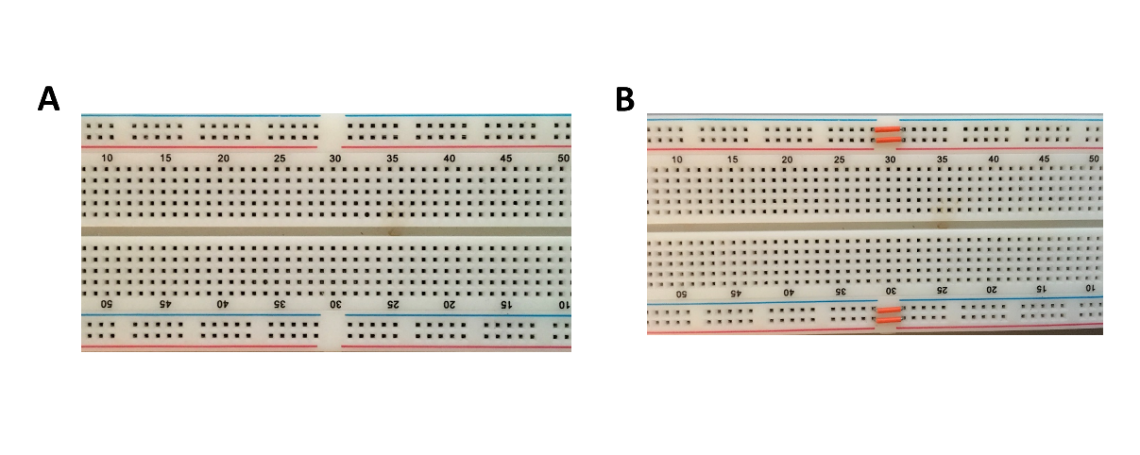
*

***Supplementary Figure 2.*** *(A) Breadboard with a break in the power rails in the middle. (B) Same board but with wires connecting rails on the left side to those on the right side.*

************************************************************************************

Now we want to set up the rail splitter. This is the circuit:


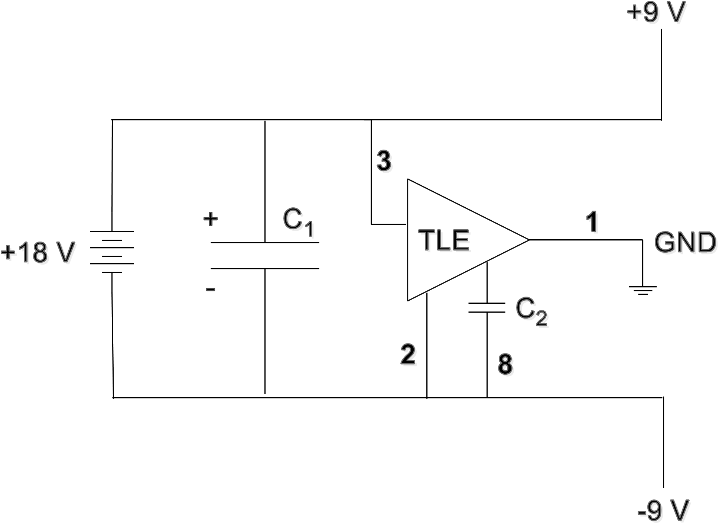


**Supplementary Figure 3.** Rail splitter circuit. The +18 V is the difference between the red and blue power rails at the bottom of the breadboard. *C_1_* is provided by electrolytic capacitors (200 μF) and *C_2_* is provided by a ceramic capacitor (1 μF). *TLE* is the TLE 2426 IC chip; the dark numbers refer to the IC chip’s pins. The circuit creates a virtual ground that will be 9 V more negative than the (lower) red power rail and 9V more positive than the (lower) blue power rail. We will put this virtual ground at the (upper) blue power rail.

There should be a capacitance of 200 μF between the positive (red) and negative (blue) power rails. The Sparkfun capacitor kit does not include a 200 μF capacitor, but it does include many 100 μF capacitors. Capacitors in parallel sum together and so we can put two of them in parallel between the positive and negative rails. These are electrolytic capacitors and they have a polarity: the negative end is marked by a white stripe, a negative symbol, and shorter wire. Stick the positive ends in holes in the red rail and the negative ends in holes in the blue rail. It helps to trim the wires first so that they are the same length; you want both to fit securely in the breadboard holes.

The pinout diagram of the TLE 2426 IC chip looks like this:


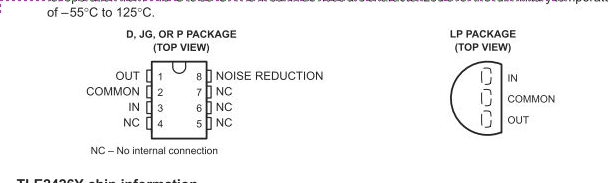
**Supplementary Figure 4.** The pinout of the TLE 2426 IC chip. The pins labeled “NC” are not connected. The other pins go to virtual ground (1), negative power rail (2), positive power rail (3), via 1 μF to negative power rail (8).

Put it in the breadboard and make the requisite connections. The breadboard should now look something like this:


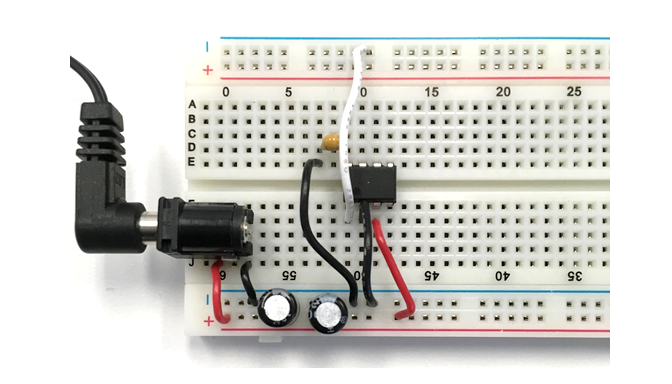
**Supplementary Figure 5.** Power supply circuit on the breadboard. The yellow element is the 1 μF capacitor. Unlike the electrolytic capacitors, it does not have a polarity: just stick one end in the same row as pin 8 of the TLE 2426 and connect the other end (here via a black wire) to the negative (blue) power rail. The white wire connects pin 1 to the virtual ground rail (upper blue). The right-most black wire connects pin 2 to the negative power rail (lower blue). The right-most red wire connects pin 3 to the positive power rail (red).

**GND**

**-9 V**

**+9 V**

A few things to note about this figure. (1) You can see the white stripes of the two 100 μF capacitors along the negative power rail. (2) 1 μF capacitor is inserted in breadboard row 7 (connected by a black wire to the negative power rail) and breadboard row 9 (same row as pin 8 of the TLE 2426). (3) There is a circle in the lower left corner of the TLE 2426 chip (easier to see in the high-quality image). This is analogous to the semi-circle at the top of the pinout diagram in Suppl. Fig. 4. By spotting it, you can tell which pin is which. Given how the chip is oriented in this picture, the pins at the bottom are 1, 2, 3, and 4 (left to right) and those at the top are 8, 7, 6, and 5 (left to right).

You should plug in the wall adapter and use a multimeter to check that the positive power rail (lower red) is approximately 9 V more positive than the virtual ground (upper blue) and that the negative power rail (lower blue) is approximately -9 V more negative than the virtual ground. You will likely measure small differences from these numbers (0-2%) depending on your wall adapter; that is fine, as these differences can be corrected in software through the calibration procedure.

**III. Follower / voltage divider / differential amplifier (Figure 2B)**

The op-amp IC we use throughout is the LM358. Here is its pinout diagram:


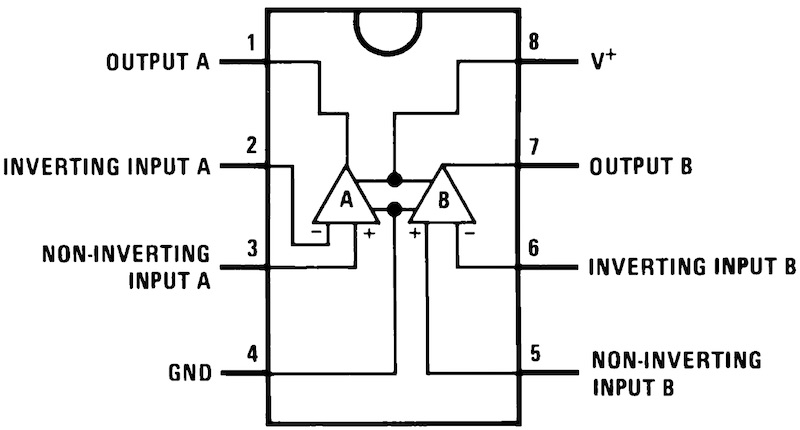


**Supplementary Figure 6.** LM358 integrated circuit chip. It contains two separate op-amps, which use common power supplies. In this diagram, pin 4 is labelled “GND” but really pin 4 is for the negative power supply. In some circuits, this will indeed be ground, but in our circuits it will be -9 V.

One nice thing about the LM358 is that it contains two separate op-amps, and so we can use one of these IC chips to create two separate circuits. In this section, we use a single LM358 to create both the follower and differential amplifier of main text Fig. 2B, along with a voltage divider.

First, let’s make the follower, as in the following figure.


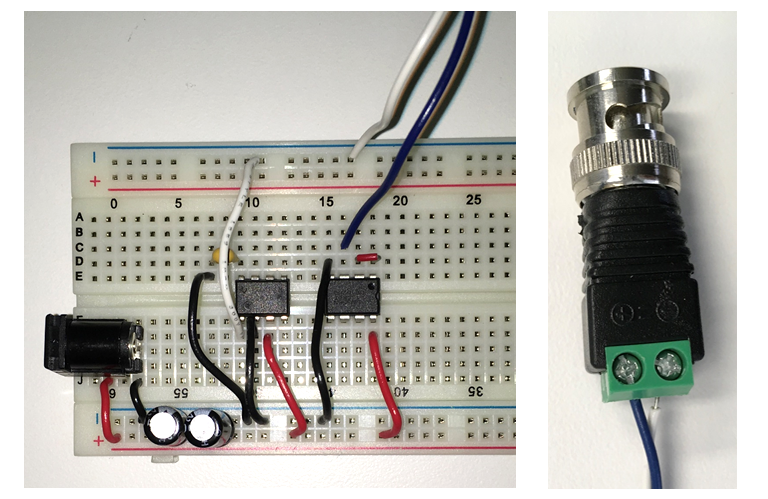


**Supplementary Figure 7.** Follower circuit added to the power supply of Suppl. Fig. 4. The dot at the upper right on the LM358 chip is analogous to the semi-circle of Suppl. Fig. 5 and helps orient us. The bottom pins are 5, 6, 7, and 8 (left to right), whereas the upper pins are 4, 3, 2, and 1 (left to right). The blue wire in row 16 (connected to pin 3) is the input signal from the patch clamp amplifier ($V_{IN}$). The amplifier’s reference is the white wire connected to the virtual ground (upper blue rail). The blue and white wires are connected to the BNC connector at right: blue goes to + and white goes to -.

In making the follower we used the op-amp in the upper half of the IC. We connected the pins 8 and 4 of the IC to the positive and negative power rails, respectively. We connected pins 1 and 2 (output and inverting input) via the little red wire (this just shorts them together). And we used a BNC connector (Suppl. Fig. 6 right) to connect them to the BNC output of the patch clamp amplifier. (Pictured is a male BNC connector. Similar female connectors are also available, if that would be more convenient.)

The output of the op-amp circuit is pin 1 (breadboard row 18 in the picture). Next, we will construct a voltage divider to scale the output down.


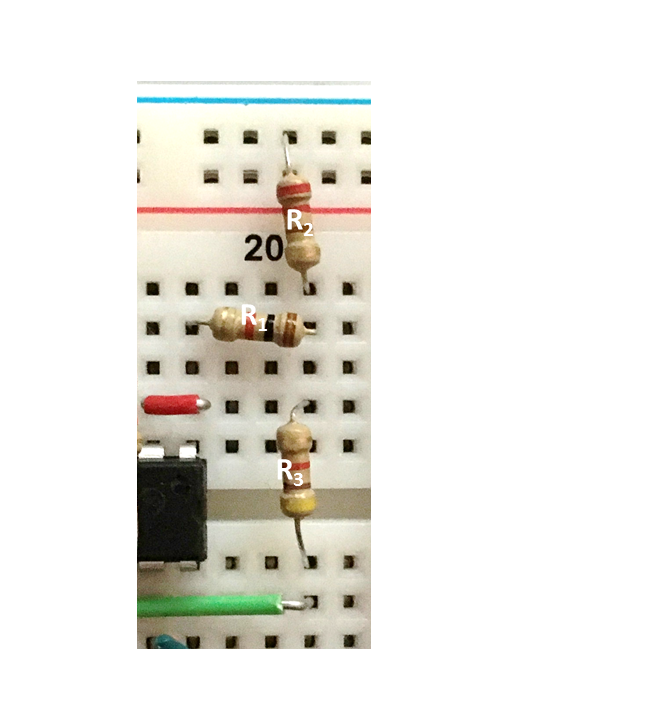


**Supplementary Figure 8.** Voltage divider. The output of the follower circuit (breadboard row 18) goes to one end of resistor R_1_ (using the nomenclature of main text Fig. 2B). This is in a voltage divider configuration with resistor R_2_. One end of R_2_ is connected to the virtual ground (upper blue rail). The other end, which is also connected to an end of R_1_ goes to resistor R_3_, which connects (via the green wire) to the non-inverting input of the other (lower) op-amp of the IC chip.

The green wire of Suppl. Fig. 8 should go to the non-inverting input of the op-amp in the lower half of the LM358 chip (pin 5 of pinout diagram of Suppl. Fig. 5). This pin is also connected to the positive (+9 V) power rail via a resistor (R_4_ of main text Fig. 2B).

Completing the differential amplifier by adding resistors R_5_ and R_6_, we have Suppl. Fig. 9.


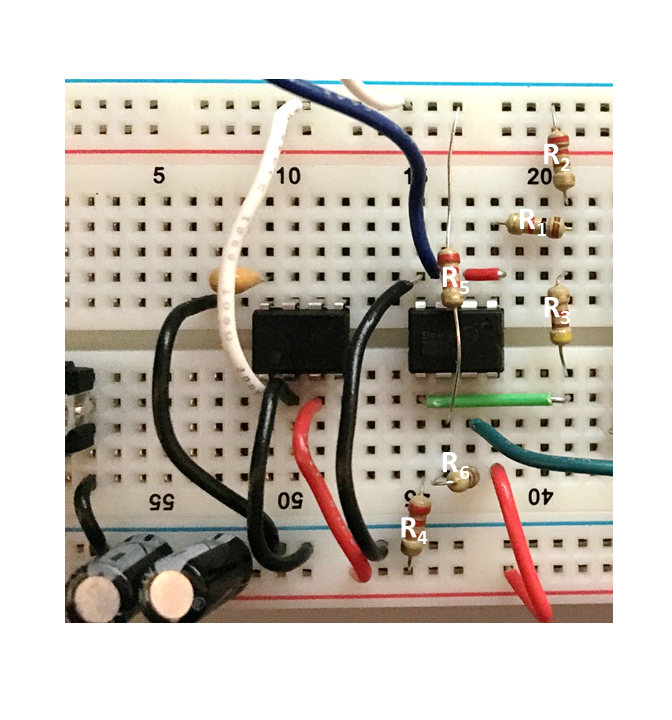


**Supplementary Figure 9.** The circuit now includes a power supply, a follower, a voltage divider, and a differential amplifier (main text Fig. 2A and 2B). The three new resistors are connected as follows: R_4_ goes from pin 5 of the LM358 (non-inverting input B) to the positive power rail (red); R_5_ goes from pin 6 of the LM358 (inverting input B) to the virtual ground; and R_6_ connects pin 6 to pin 7 (output B). The dark green wire also coming from pin 7 (breadboard row 43) goes to the ADC input of the Teensy microcontroller.

The output of this circuit ($V_{OUT}$, the dark green wire exiting stage right in the picture) should be related to the input ($V_{IN}$, dark blue wire of breadboard row 16) like this:

$$V_{OUT}=(1+ \frac{R_{6}}{R_{5}})\left[ \frac{R_{4}R_{2}}{\left( R_{3}+R_{4} \right)\left( R_{1}+R_{2} \right)}V_{IN}+\frac{R_{3}}{\left( R_{3}+R_{4} \right)}(9) \right]$$

The number *9* at right comes from the +9 V of the positive power rail.

It would be useful to check that this relationship is (approximately) true. If it’s not, then that is a strong indication that you should recheck all the connections using your multimeter – especially make sure that all the resistors and wires are well seated in the breadboard holes. This is especially a concern with the resistors, as their wires are thin and a bit too prone to bend in odd ways. In order to check the circuit, apply +3 V, -3 V, and 0 V to $V_{IN}$. If you’re using two AAA batteries and a battery holder, the simplest way to do this is: (1) remove the blue and white wires going to the BNC connector (Suppl. Fig. 7), (2) replace them with the red and blue wires of the battery holder (+3 V) and measure $V_{OUT}$, (3) swap the red and blue wires to get -3 V and measure $V_{OUT}$, and (4) remove the battery holder, short pin 3 of the LM358 (upper row 16 in Suppl. Fig. 9) directly to the virtual ground to get 0 V, and measure $V_{OUT}$. Given the resistor values from the paper (R_1_=2200 Ω, R_2_=470 Ω, R_3_=4700 Ω, R_4_=22000 Ω, R_5_=10000 Ω, and R_6_=100 Ω), you should approximately get $V_{OUT}$ = 2.04 V for $V_{IN}$ = 3 V, $V_{OUT}$ = 1.16 V for $V_{IN}$ = -3 V, and $V_{OUT}$ = 1.60 V for $V_{IN}$ = 0 V. If you use different resistor values, you should calculate the expected values for yourself. Remember that the numbers need only be *approximately* correct (within 5%) – large deviations indicate the connections are incorrect, but small deviations suggest only that the components are imperfect or the batteries are running down. Small deviations can be corrected by the calibration procedure for the software.

**IV. Teensy 3.6 connections (Figure 2C)**

Here (Suppl. Fig. 10) is the pinout diagram of the Teensy 3.6 microcontroller.

**
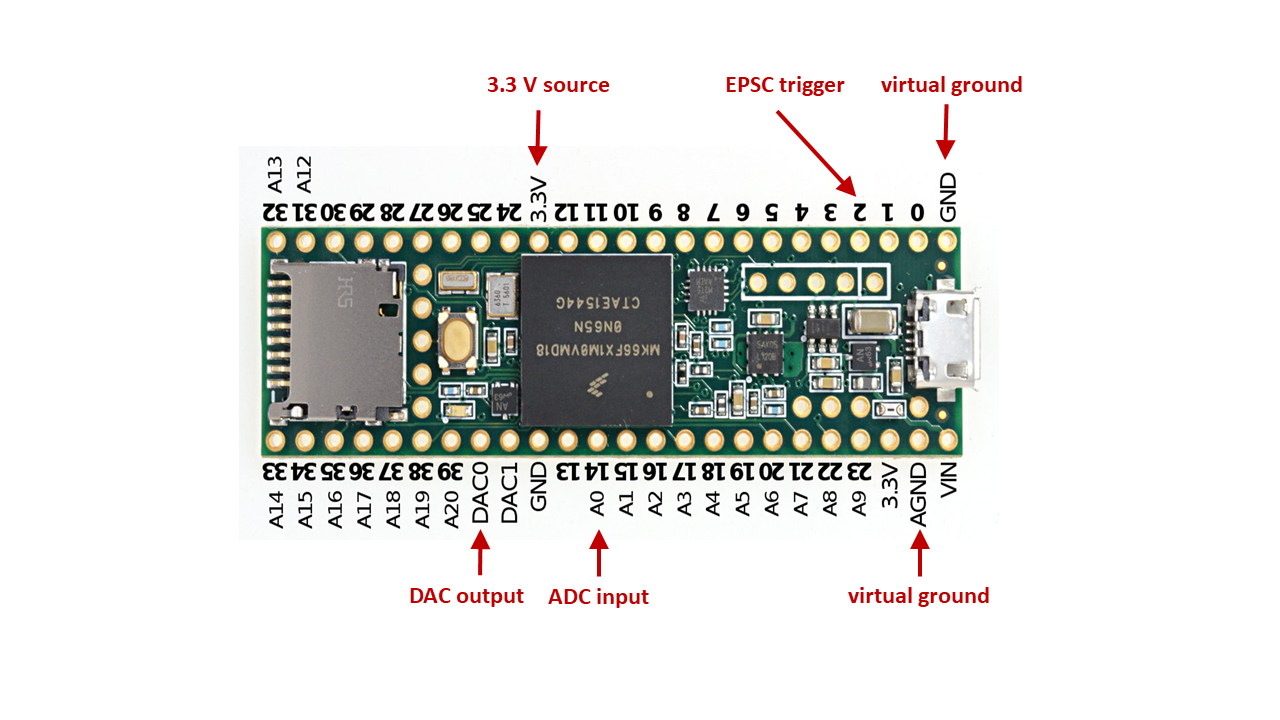
**

**Supplementary Figure 10.** Pinout diagram of the Teensy 3.6. Shown are the connections the Teensy makes with other parts of the system: the output of Suppl. Fig. 8 ($V_{OUT}$) goes to A0 (ADC input); the virtual ground (upper blue rail) is connected to both the GND pin at upper right and the AGND pin at lower right; the Teensy output (DAC output) will go to the differential amplifier we will construct in Part V); and the 3.3 V source is indicated for those who wish to check their circuits.

And here are how the connections look on a breadboard.


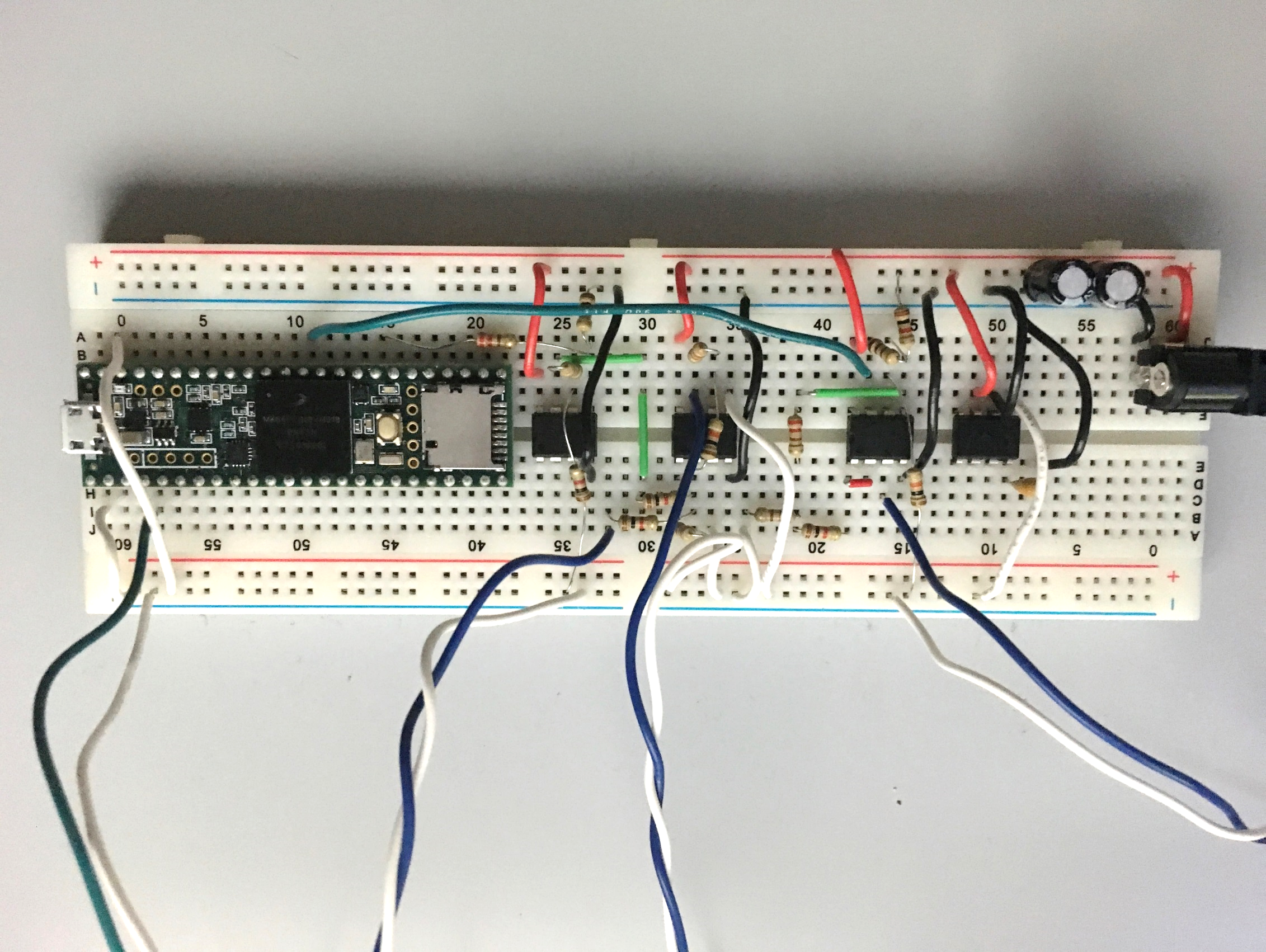


**Supplementary Figure 11.** Teensy connections. The dark green wire at upper right transmits a TTL trigger for EPSCs. It connects pin 2 of the Teensy (breadboard row 58 in the picture) with the positive terminal of a BNC connector. The connector’s negative terminal is attached to a white wire that connects to the virtual ground (upper blue rail). The output of the Teensy microcontroller (DAC0, breadboard row 15 in the picture) goes via a resistor (R_7_, in the nomenclature of main text Fig. 2) to the upcoming differential amplifier (main text Fig. 2D).

**V. Differential amplifier (Figure 2D)**

The output of the Teensy microcontroller (DAC0 in Suppl. Fig. 11, breadboard row 15 in Suppl. Fig. 10) goes to the differential amplifier of main text Fig. 2B via a resistor R_7_.


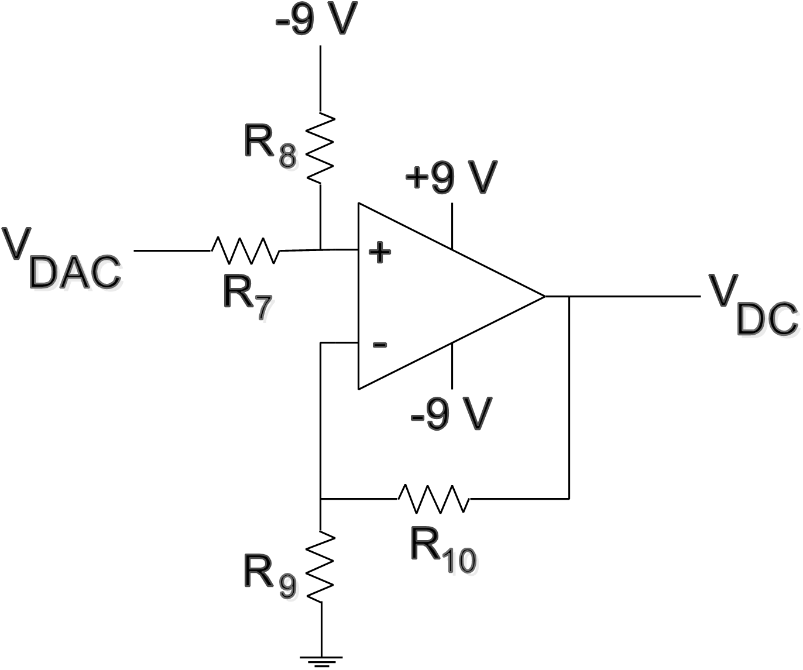
**Supplementary Figure 12.** The output (DAC0) of the Teensy microcontroller is V_DAC_. We connect it to a resistor that goes to the non-inverting input of the op-amp.

Again, we use a LM358 IC chip to provide an op-amp (Suppl. Fig. 6). But here we only use the lower op-amp.


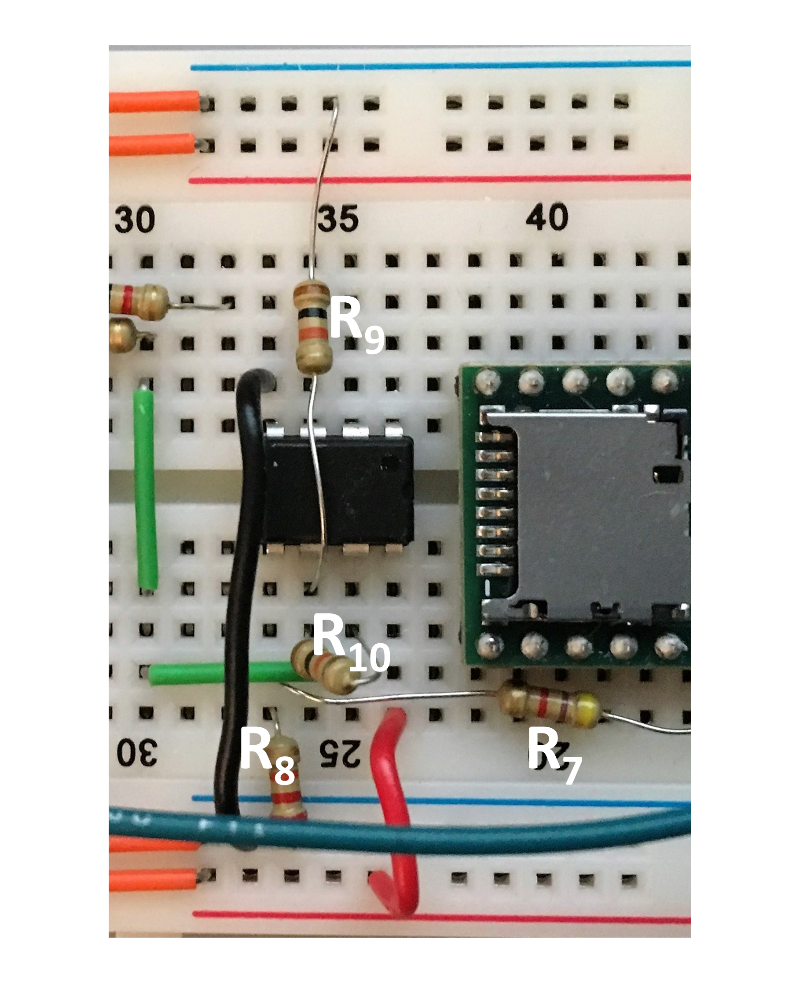


**Supplementary Figure 13.** The LM358 pinout diagram was given in Suppl. Fig. 6. The dot and semi-circle can be seen on the right side of the chip – so the lower pins are 5, 6, 7, and 8 (left to right), and the upper pins are 4, 3, 2, and 1 (left to right). The output of the lower op-amp is pin 7 (breadboard row 25.) The bright green wires connect it to breadboard row 30 of the upper half.

To test the differential amplifier circuit, do the following: (1) Remove the Teensy from the breadboard. (2) Apply +3 V to the right-hand side of resistor R_7_. If you’re using a battery pack, this means you should attach the pack’s ground (black wire) to the breadboard’s virtual ground, and the pack’s positive connection (red wire) to breadboard row 15. (3) Use the multimeter to measure the voltage difference between the op-amp’s output (its pin 7, on breadboard row 25) and the virtual ground. For the resistors of the main text (R_7_ = 4700 Ω, R_8_ = 22000 Ω, R_9_ = 10000 Ω, and R_10_ = 10000 Ω), the voltage difference should be roughly 1.78 V. Now repeat steps (1)-(3), but apply 0 V in step (2) by connecting the right-hand side of resistor R_7_ to ground. The voltage difference should now be -3.17 V, given the resistors of the main text. Finally, reattach the Teensy to the breadboard.

**V. Summing (Figure 2E)**

The fifth and last part of the system sums the dynamic clamp voltage command and the current clamp voltage command (main text Fig. 2E). This involves two inverting amplifiers in series.


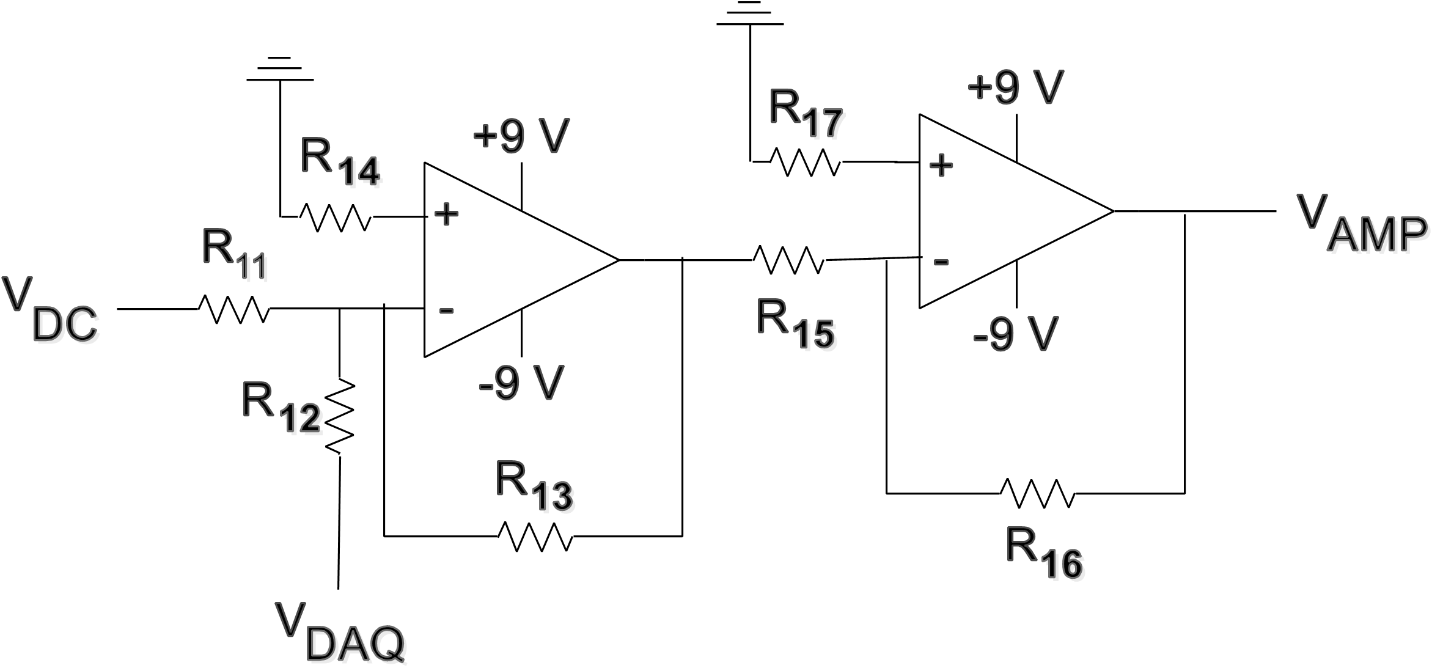


**Supplementary Figure 14.** Two summing circuits in series. V_DC_ is the output of the differential amplifier of Suppl. Fig. 12; V_DAQ_ is the output of the DAQ system (current clamp command).

The LM358 contains two op-amps and so we can construct both inverting amplifiers using just one. This is what the breadboard portion looks like (Suppl. Fig. 15):

**
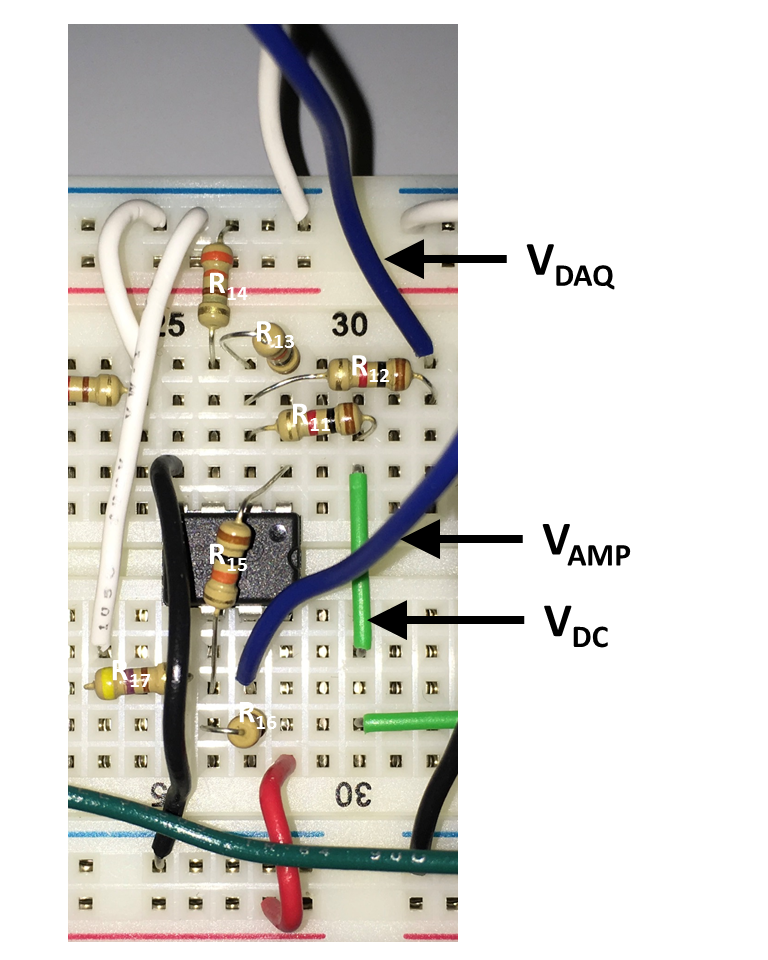
Supplementary Figure 15.** Summing circuit. The op-amp of the upper half of the IC sums and inverts signals from the dynamic clamp system (V_DC_, green wire ascending to breadboard row 30) and the current clamp system (V_DAQ_, dark blue wire going to breadboard row 32). The op-amp of the lower half inverts the signal back. Its output, which goes to the patch clamp amplifier, is carried by the dark blue wire called V_AMP_. The resistors are as defined in main text Fig. 2E. (NOTE: The orange jumper wires connecting the power rails of the two sides, visible in Suppl. Fig 13, are missing in this one. But they should be there.)

To test this circuit, temporarily remove the connections going to the right-hand side of resistors R_11_ and R_12_ (in the picture of Suppl. Fig. 15, this means remove the vertical green wire labeled $V_{DC}$ and the blue wire labeled $V_{DAQ}$). Connect one of the resistors directly to the virtual ground with a wire and the other to +3 V using the battery pack (or your signal generator or your DAQ board). Check that the output ($V_{AMP}$, pin 7 of the IC, breadboard lower row 27) also equals +3 V. Now reverse the polarity of the input voltage and check that the output voltage is now -3V. Repeat this procedure with the other input resistor. If all is well, you can reconnect the green wire $V_{DC}$ and the blue wire $V_{DAQ}$.

**VI. All together**

This (Suppl. Fig. 16) is what the complete breadboard circuit looks like.


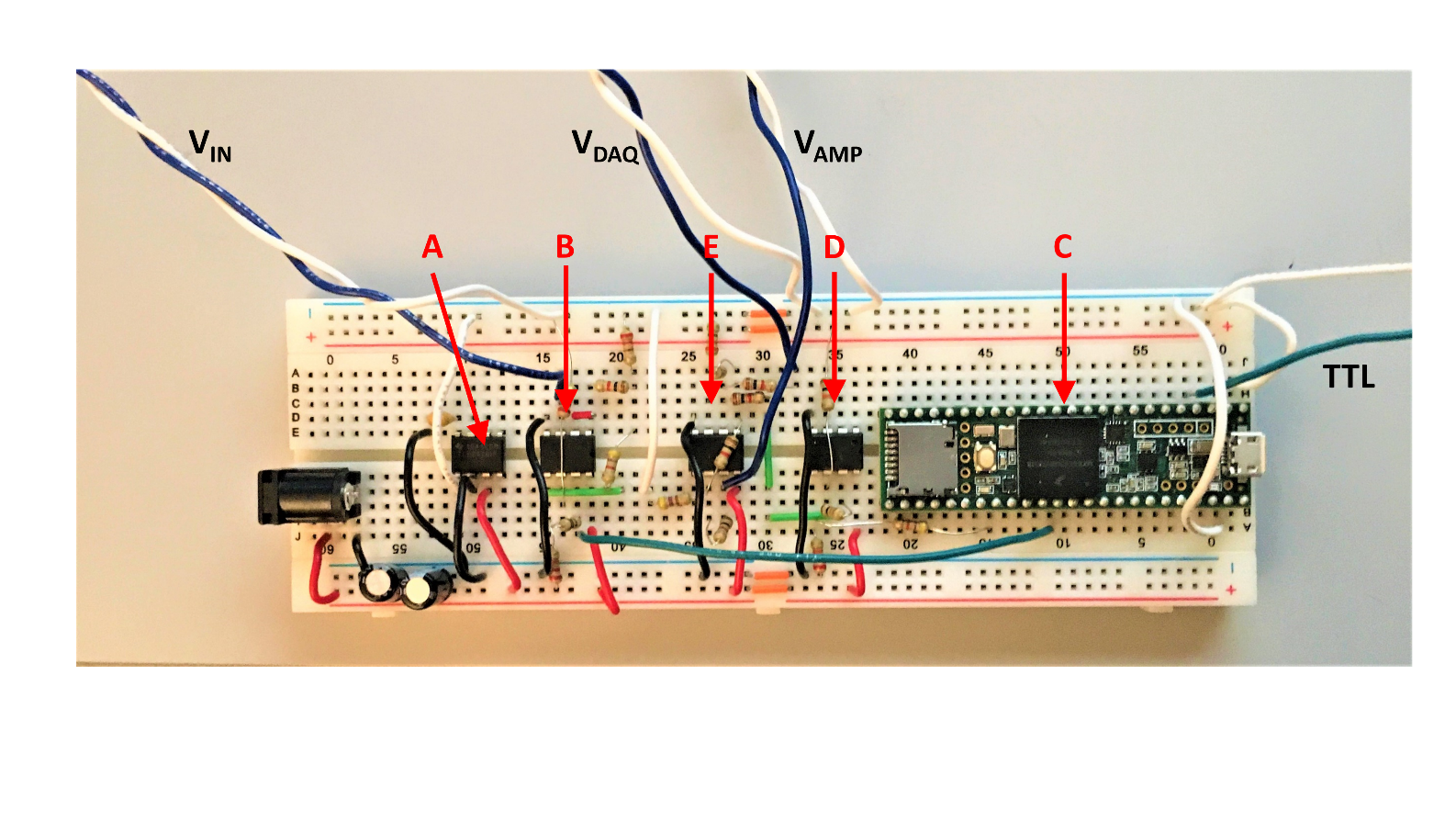


**Supplementary Figure 16.** The voltages (V_IN_, V_DAQ_, V_AMP_, and TTL) are the same as those of main text Fig. 2. The letters (A, B, C, D, E) refer to the five panels of main text Fig 2.

And this (Supp. Fig. 17) is what the complete circuit looks like in an electronic schematic:


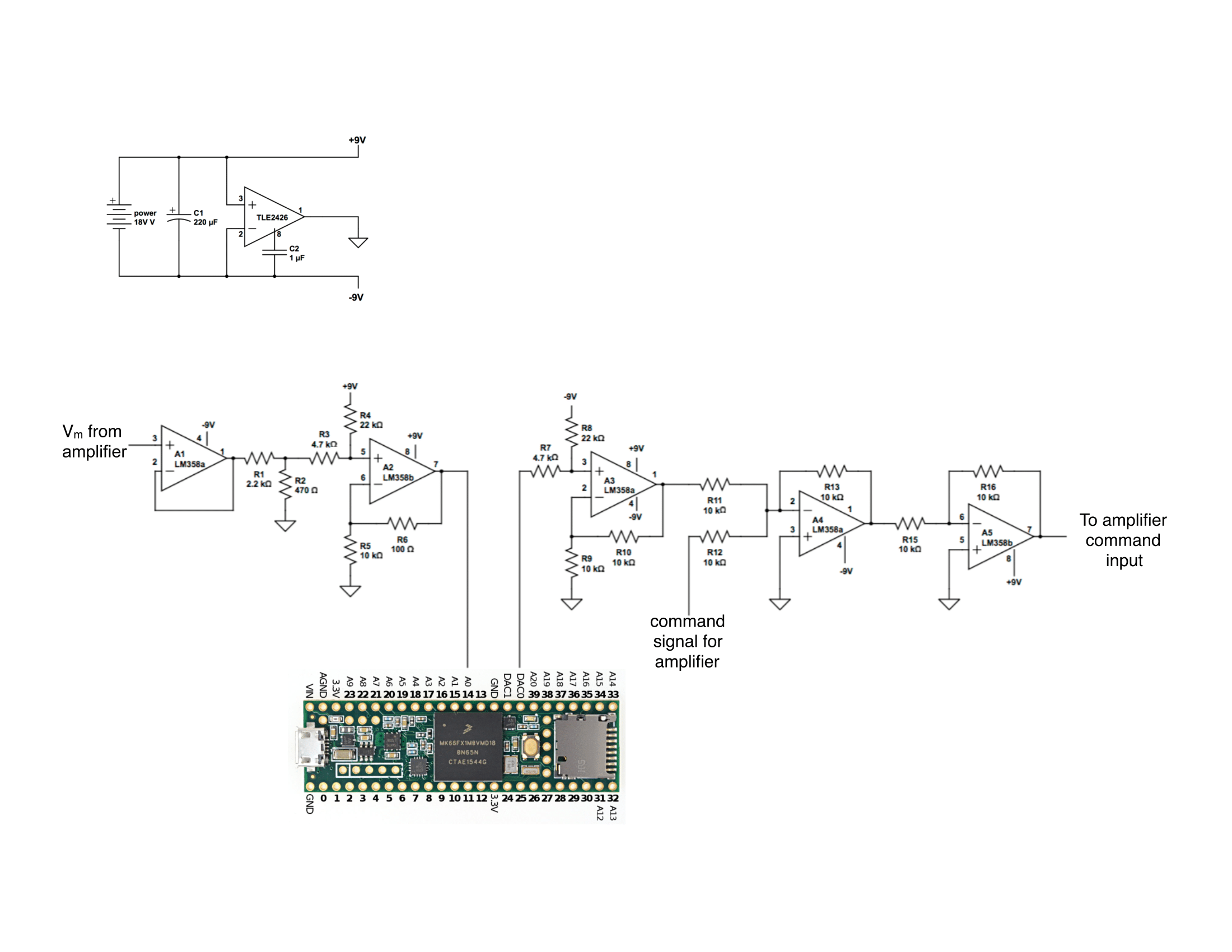


**Supplementary Figure 17.** Electronic schematic of full system.
