# Supplementary material for: A Dynamic Clamp on Every Rig
Source: eNeuro. 2017 Oct 23;4(5):ENEURO.0250-17.2017. doi: 10.1523/ENEURO.0250-17.2017 (PMC5659377; doi:10.1523/ENEURO.0250-17.2017)
Supplement: Extended Data 1 [file enu005172417so1.zip › Extended_data/3_Obtaining_and_installing_Arduino_and_Processing/Obtaining_and_installing_Arduino_and_Processing.docx]

**Obtaining and installing Arduino and Processing**

**ARDUINO**

The Arduino website is [www.arduino.cc](http://www.arduino.cc).

1) Download the Arduino software. The site offers an online IDE (called “Arduino Web Editor”), but we have not been able to get that to work with the Teensy 3.6. So stick with the standard IDE. At this writing (05/29/17), it is Arduino 1.8.2. This works with Windows, Mac, and Linux. Follow the installation instructions given by the Arduino site.

To allow Arduino software to communicate with the Teensy 3.6 microcontroller, we use an add-on called Teensyduino. Here is the website: <https://www.pjrc.com/teensy/td_download.html>.

2) Download Teensyduino (Windows, Mac, or Linux) and follow the installation instructions given by the site.

Attach the Teensy microcontroller to your computer through a USB port. Make note of the port name (on Windows, the name will be something like “COM3”; on Mac, something like “/dev/tty.usb3232”; on Linux, something like “ttyS3”.)

3) Open the Arduino software. From the Tools menu, make sure that the Board selected is the Teensy 3.6 and that the Port is the USB port to which you attached the Teensy.

To make sure everything is working properly, run the Blink program. This is like the microcontroller version of “Hello, world!” How to do so is explained here: <https://www.pjrc.com/teensy/td_usage.html>. (One correction: Blink can actually be found at File > Examples > Basic > Blink.) The page features a Teensy 2.0, which is physically smaller than the 3.6, but the process is the same. Note the location of the pushbutton on the Teensy 3.6. If the microcontroller ever behaves oddly, and especially if you’re not sure whether a program has successfully uploaded, try pushing the button; it acts like a hard reset.

**PROCESSING**

The Processing website is <https://processing.org>.

1) Download version 2.2.1. A newer version has recently been made available, but the library we used to create the GUI (ControlP5) has not yet been updated to match it (as of 05/29/17).

2) Open Processing. Add the ControlP5 library by selecting Sketch > Import Library … > Add Library …, searching for ControlP5, and installing it.

For future reference, this is the ControlP5 website: <http://www.sojamo.de/libraries/controlP5/>.
