# Supplementary material for: A Dynamic Clamp on Every Rig
Source: eNeuro. 2017 Oct 23;4(5):ENEURO.0250-17.2017. doi: 10.1523/ENEURO.0250-17.2017 (PMC5659377; doi:10.1523/ENEURO.0250-17.2017)
Supplement: Extended Data 1 [file enu005172417so1.zip › Extended_data/4_Using_and_modifying_Arduino_software/Using_and_modifying_Arduino_software.docx]

**Using and modifying Arduino software**

**I. Using the Arduino software**

You should already have installed Arduino and Teensyduino following the instructions in the document *Obtaining_and_installing_Arduino_and_Processing*.

Copy the folder *dynamic_clamp* to some place on your computer, such as the Arduino folder.

Find the main file *dynamic_clamp.ino* inside that folder and open it in Arduino. Make sure that your Teensy 3.6 is connected and recognized by going to the Tools menu. It should look something like this:


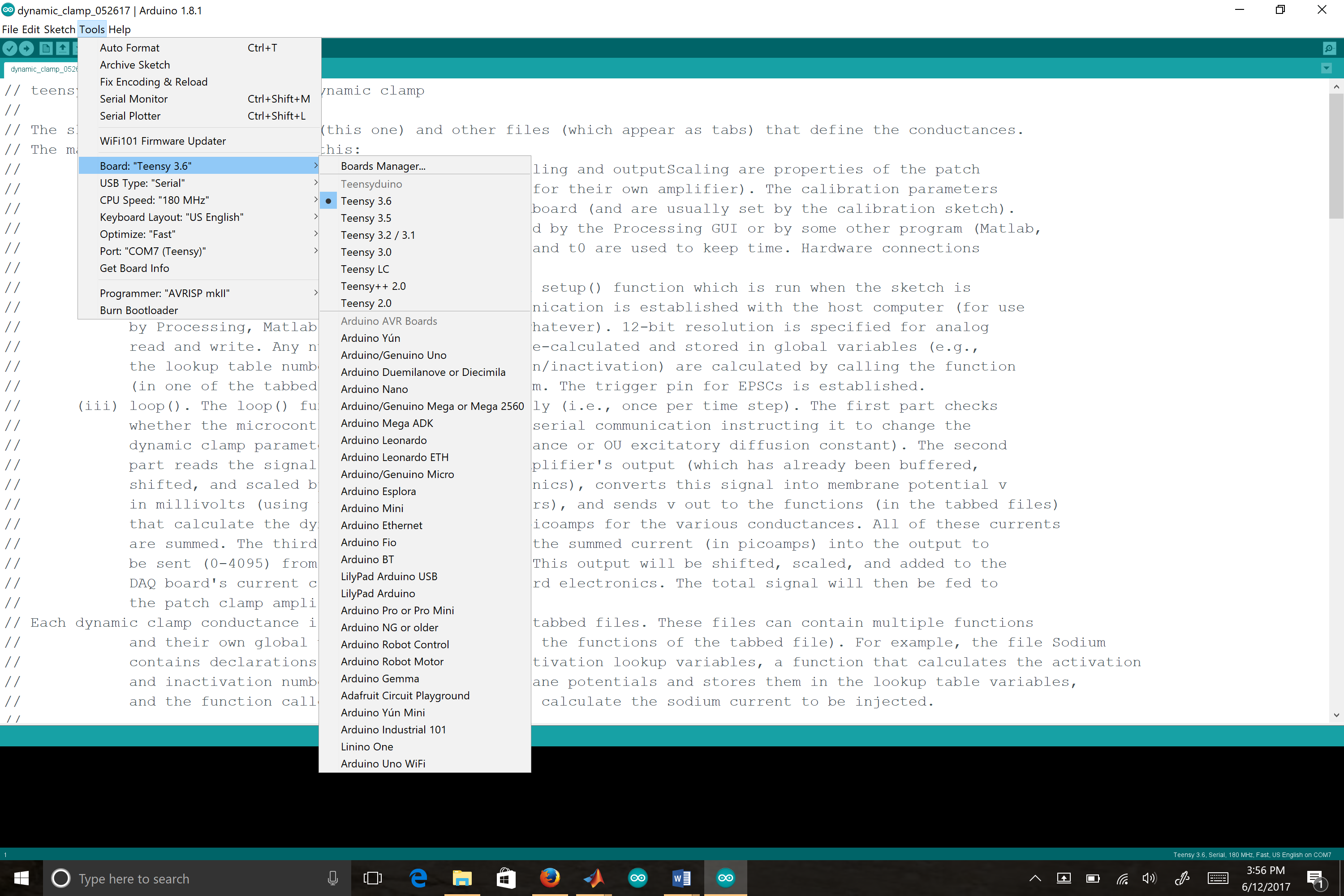


In our case, as you can see, the Teensy is located at COM7. Make note of the name of the USB port for your board.

Compile and upload the program to the microcontroller by pressing the right-facing arrow at the upper left of the window. It might take as long as a minute for the process to complete; be patient. The little window the Teensy opens up should flash “Rebook OK” if all has gone well. If you don’t see that (comforting) message, press the pushbutton on the Teensy (it is just in front of the microSD port); this will force an upload.

The program should now be on the microcontroller, and you can use the Processing GUI to control the dynamic clamp simulation (see *Using_and_modifying_Processing_software).*

Note that you need upload the program only once. It will remain on the microcontroller indefinitely.

**II. Modifying the Arduino software**

To modify the conductances or add new ones, you should understand the program structure. The program consists of multiple files. Opening the main file *dynamic_clamp* also opens the other Arduino files contained in the same folder. These appear in the tabs.

**Main file**

The main file consists of three parts: (1) global variables, (2) setup function, and (3) loop function.

*(1) Global variables*

These are variables accessible to all functions. The only ones that users are likely to wish to change are the ones that determine how the electronic circuits are calibrated.

// Scaling for the patch clamp amplifier

float inputScaling = 50.0; // number of millivolts sent out by amplifier for each millivolt of membrane potential

// e.g., with a scaling of 50, if the membrane potential is -65 mV, the amplifier outputs -3.25 V

float outputScaling = 400.0; // number of picoamps injected for every volt at the amplifier's command input

// e.g., if the DAC or the Teensy outputs -0.5 V, this is interpreted as -200 pA by the amplifier

// when the scaling is 400

// Calibrating the input/output numbers given the resistor values and power supply values of the breadboard

// N.B.: these parameters (numerators) are properties of the components on the breadboard;

// they are independent of the amplifier and DAQ board

const float inputSlope = 7.67812538/inputScaling;

const float inputIntercept = -15940.06952/inputScaling;

const float outputSlope = 757.2606458/outputScaling;

const float outputIntercept = 2417.9572;

We describe how to set these numbers in a separate supplementary document, *Calibration_procedure*.

Note that the global variables include all the conductance values (e.g., g_shunt, g_HCN, g_Na).

*(2) Setup function*

The setup function is run once, when the program is uploaded to the microcontroller. It mostly involves housekeeping functions (initializing the serial port to communicate with Processing or whatever program will set the conductance values, specifying the analog resolutions, specifying which pin is to be used when triggering EPSCs). It also calls the functions that generate the lookup tables used by several of the conductances; these functions are contained within the (tabbed) files that define those conductances.

The use of lookup tables (see, for example, <https://en.wikipedia.org/wiki/Lookup_table>) is a common tactic in real-time systems. Rather than doing calculations at runtime, one pre-calculates numbers for a wide range of parameters and then simply looks up the number for a given parameter when it is needed. This can save a lot of time. In the case of Hodgkin-Huxley-style conductances, one pre-calculates the steady-state values and time constants of gating variables for a wide range of membrane potentials. At every time step, rather than doing the calculation again, one finds the pre-calculated steady-state value and time constant most closely corresponding to the measured membrane potential and uses them in subsequent operations. Again, looking up numbers in a saved list tends to be much faster than doing calculations.

*(3) Loop function*

Every Arduino sketch contains a loop function. It is run constantly (i.e., at every time step).

Our loop function contains three parts. The first part checks the USB port for a serial communication instructing the microcontroller to change the values of the various conductances. Whenever the port receives 32 bytes (4 bytes for each of the eight expected numbers), the conductance values are updated.

The second part reads the membrane potential (using the calibration parameters to map the measured analog input into the neuron’s membrane potential) and uses this to calculate the current the dynamic clamp conductances should inject. It does this by calling the functions of all the active dynamic clamp conductances.

The third part transforms the total dynamic clamp current into an output to be sent through the Teensy’s analog output (DAC) (using the calibration parameters). Note that these numbers are limited to the range 0-4095 (12 bit).

**Tabbed files**

Each tabbed file contains the specifications for a single conductance. If you wish to modify any given conductance, this is the only file that you need change.

As an example, consider the transient sodium conductance. The code is reproduced on the next page.

There are three sections.

The first simply declares arrays in which to store the activation and inactivation parameters of the Hodgkin-Huxley formulation: α_m_, β_m_, α_h_, and β_h_ (Johnston and Wu, 1994). Each of these is a function of membrane potential V_m_. We assumed that only the potentials between -100 mV and +50 mV were likely to be important, and that 1 mV precision was sufficient. So each array stores 151 numbers (one for each millivolt between -100 and +50).

Declaring the array variables simple assigns them a place in memory, it doesn’t fill in their values for different values of V_m_. That is instead done by the second section, the function called GenerateSodiumLUT. This function is called by the setup function of the main file when the program is first uploaded to the board. There are three things you should note about this function. (1) The arrays are indexed beginning at 0, so that the first element of the array alphaM is alphaM[0] and its last element is alphaM[150]. The corresponding voltages are 100 less than the index number; that is why the line “v = (float)x – 100.0;” is there. Also, the (float) part simply converts the integer x into a floating point (32-bit number with a decimal point). (2) Rather than use the function *exp(A)* to exponentiate an argument A, we instead use the function *expf(A)*. The difference is that *expf* explicitly tells the compiler that its argument will be a 32-bit floating point number, as opposed to some other data type, and to use the Teensy’s built-in floating point unit (FPU) for the calculation. If we had instead used
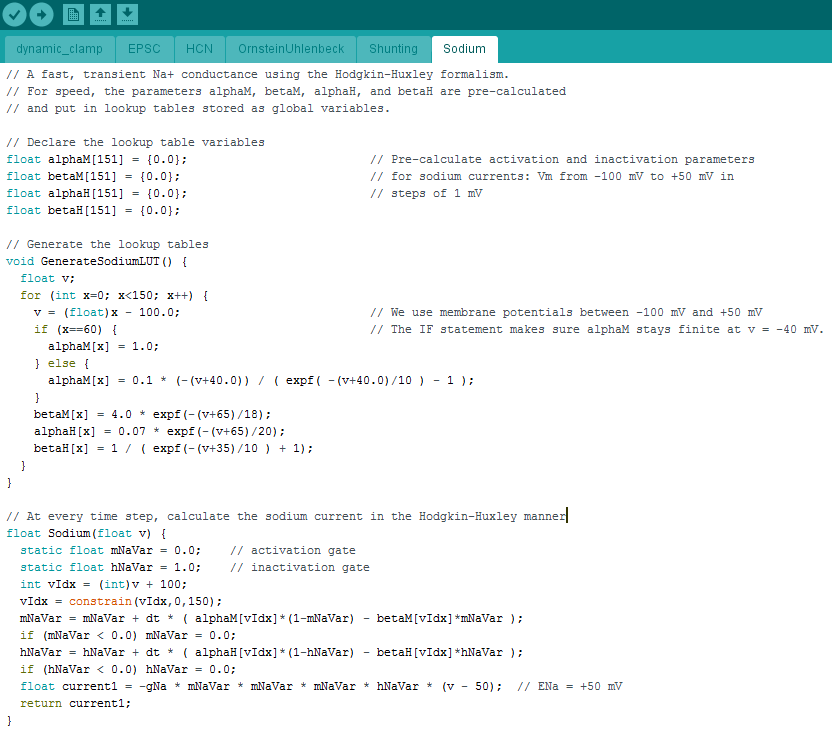
*exp,* the compiler might have failed to take advantage of the speed enhancement the FPU offers for calculations involving floating point numbers. For the same reason, you should always use math functions that end in *f* over those that do not when using the Teensy (*sinf* rather than *sin*, *cosf* rather than *cos*, *sqrtf* rather than *sqrt).* See, for example, this web page: <https://tympan.org/2017-02-09-for-speedy-float-math-specify-float/>. (3) When the index x =60, the voltage equals -40 mV. This is problematic for the Hodgkin-Huxley function α_m_. If you simply plug in V_m_= -40 mV, the numerator goes to zero but so does the denominator. The ratio 0/0 is ambiguous, though inspecting the function on either side of -40 mV indicates that it should equal 1. That is why the if-else statement is there: to ensure α_m_ equals 1 when V_m_= -40 mV. You should always be aware of potential problems like this. For example, if a function *expf(a/b)* appears in a denominator and there’s any chance that *b* will ever get close to zero, it is better to write this as *expf(a)* – *expf(b).*

The third section is the function Sodium(v) that calculates the sodium current on every time step. It involves the activation and inactivation gates *mNaVar* and *hNaVar.* These are integrated using the forward Euler method. This is a simple integration method, but as long as the time step *dt* is small (and here it is quite small compared to the kinetics of the sodium gates), the method is sufficient. Nearly every previous dynamic clamp implementation used forward Euler. Here too there are three things worth noting. (1) We convert the voltage (-100 to +50 mV) back into an index (vIdx) by adding 100 – just as we subtracted 100 in GenerateSodiumLUT. (2) We use *if* statements to make sure that *mNaVar* and *hNaVar* always stay non-negative. (3) We use the built-in function *constrain()* to make sure that vIdx always stays between 0 and 150 regardless of the value of membrane potential.

**III. Adding a conductance**

In the folder *Adding_a_potassium_M_conductance*, we show step-by-step how to add a potassium M conductance to the Arduino program. We also show how to add it to the Processing GUI we use to control the dynamic clamp simulation during an experiment.
