# Supplementary material for: A Dynamic Clamp on Every Rig
Source: eNeuro. 2017 Oct 23;4(5):ENEURO.0250-17.2017. doi: 10.1523/ENEURO.0250-17.2017 (PMC5659377; doi:10.1523/ENEURO.0250-17.2017)
Supplement: Extended Data 1 [file enu005172417so1.zip › Extended_data/5_Using_and_modifying_Processing_software/Using and modifying Processing software.docx]

**Using and modifying Processing software**

**I. Using the Processing software**

You should already have installed Processing following the instructions in the document *Obtaining_and_installing_Arduino_and_Processing*.

The Processing program is in the folder *processing_control*. It consists of a single file called *processing_control.pde*. Open it by double-clicking.

Find the line that reads: “myPort = new Serial(this,"COM7",115200);” and change COM7 to the name of the USB port to which you attached the Teensy microcontroller. Save the revised file.

Press the run button (right-facing arrow at upper left) in the Processing window.

This opens up a graphical user interface that looks like this:


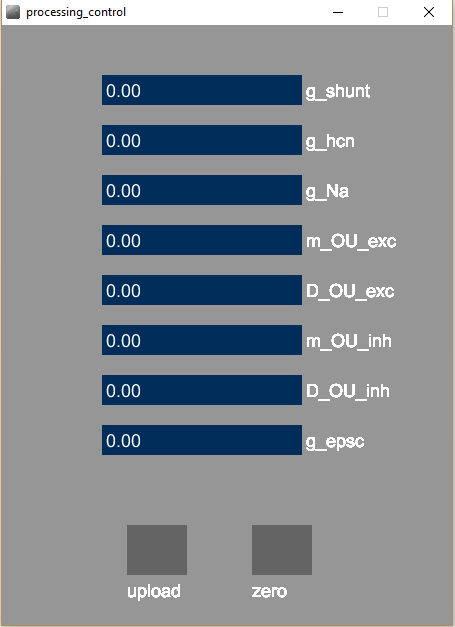
The eight parameters that can be changed during an experiment are the ones specified in the main text. All of the conductances are in nS; all of the diffusion constants are in nS^2^/ms.

When the Processing applet is first run, all the numbers are set to zero. To change them, use the sliders to move to the desired values and the press the ***Upload*** button at the bottom left.

For example, to change the shunt conductance to 5.00 nS and the HCN conductance to 1.99 nS, the window should look like the picture on the following page. When you press ***Upload***, the button will be briefly highlighted. The numbers will be sent by Processing to the Teensy microcontroller and the new conductance values will take effect immediately.


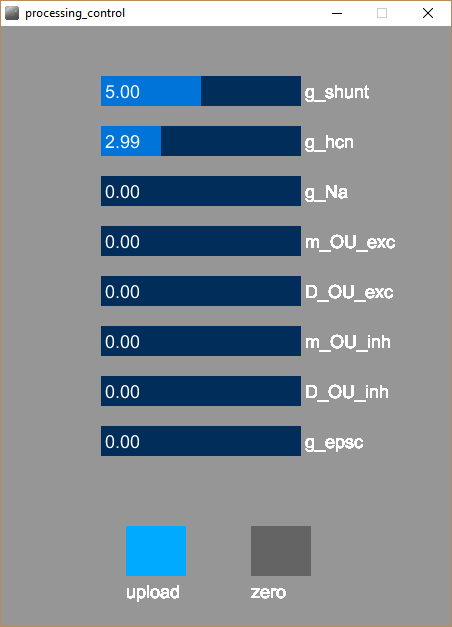
 Subsequently pressing the ***Zero*** button will zero all the numbers and return the microcontroller to its initial state.

**II. Modifying the Processing program**

In the supplementary document *Adding_a_potassium_M_conductance*, we describe both how to code the conductance in the Arduino program and to add it to the Processing program. That should serve as a good example of how to add other types of conductances.

Of the existing conductances, the only things users are likely to wish to modify the minimum value, maximum value, and default value. These are set in the *setup()* function. For example, in the case of the sodium conductance, look for this line:

dcControl.addSlider("g_Na", 0, 200, 0, 100, 150, 200, 30);

The control is explained at this web page <http://sojamo.de/libraries/archive/controlP5-0-3-14/reference/controlp5_method_addslider.htm>. In particular, the first argument (“g_Na”) is the name of the control, the second (0) is the minimum value, the third (200) is the maximum value, and the fourth (0) is the default value. The other four numbers control the x position, y position, width, and height of the slider.
