# Supplementary material for: A Dynamic Clamp on Every Rig
Source: eNeuro. 2017 Oct 23;4(5):ENEURO.0250-17.2017. doi: 10.1523/ENEURO.0250-17.2017 (PMC5659377; doi:10.1523/ENEURO.0250-17.2017)
Supplement: Extended Data 1 [file enu005172417so1.zip › Extended_data/6_Adding_a_potassium_M_conductance/Adding_a_potassium_M_conductance.docx]

**Adding a potassium M conductance**

**I. M conductance**

We wish to add a potassium M conductance to the dynamic clamp system. The conductance we have chosen is one that appears in Fransen et al., J. Neurosci. (2002). The conductance is modeled in the Hodgkin-Huxley way by a single activation gate:

$$\frac{dm(t)}{dt}=-(m\left( t \right)-m_{inf}\left( V_{m} \right))/\tau_{m}(V_{m})$$

Here, $m(t)$ represents the fraction of open M channels at an instant in time $t$. Its steady-state value depends on membrane potential $V_{m}$:

$$m_{inf}= \left\{ 1+e^{-(V_{m}+35)/5} \right\}^{-1}$$

So does its time constant:

$$\tau_{m}= \left\{ 3.3e^{(V_{m}+35)/40}+e^{-(V_{m}+35)/20} \right\}^{-1}$$

This is what they look like:


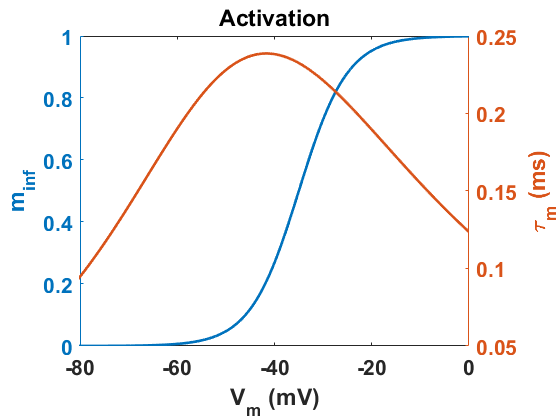


The current this conductances passes is given by:

$$I_{M}=-g_{M}m(t)\left( V_{m}-E_{K} \right)$$

where $g_{M}$ is the maximal M condutance (in nS) and $E_{K}$ is the potassium reversal potential (-80 mV).

**II. Modifying the Arduino program**

To add this conductance to the Arduino program, we have to do two things: (1) add references to the M conductance to the main file *dynamic_clamp.ino*, and (2) add a tabbed file defining the conductance.

Open the dynamic clamp program in Arduino.

*Main file*

(1) Add the maximal M conductance $g_{M}$ to the list of global variables in the first section.


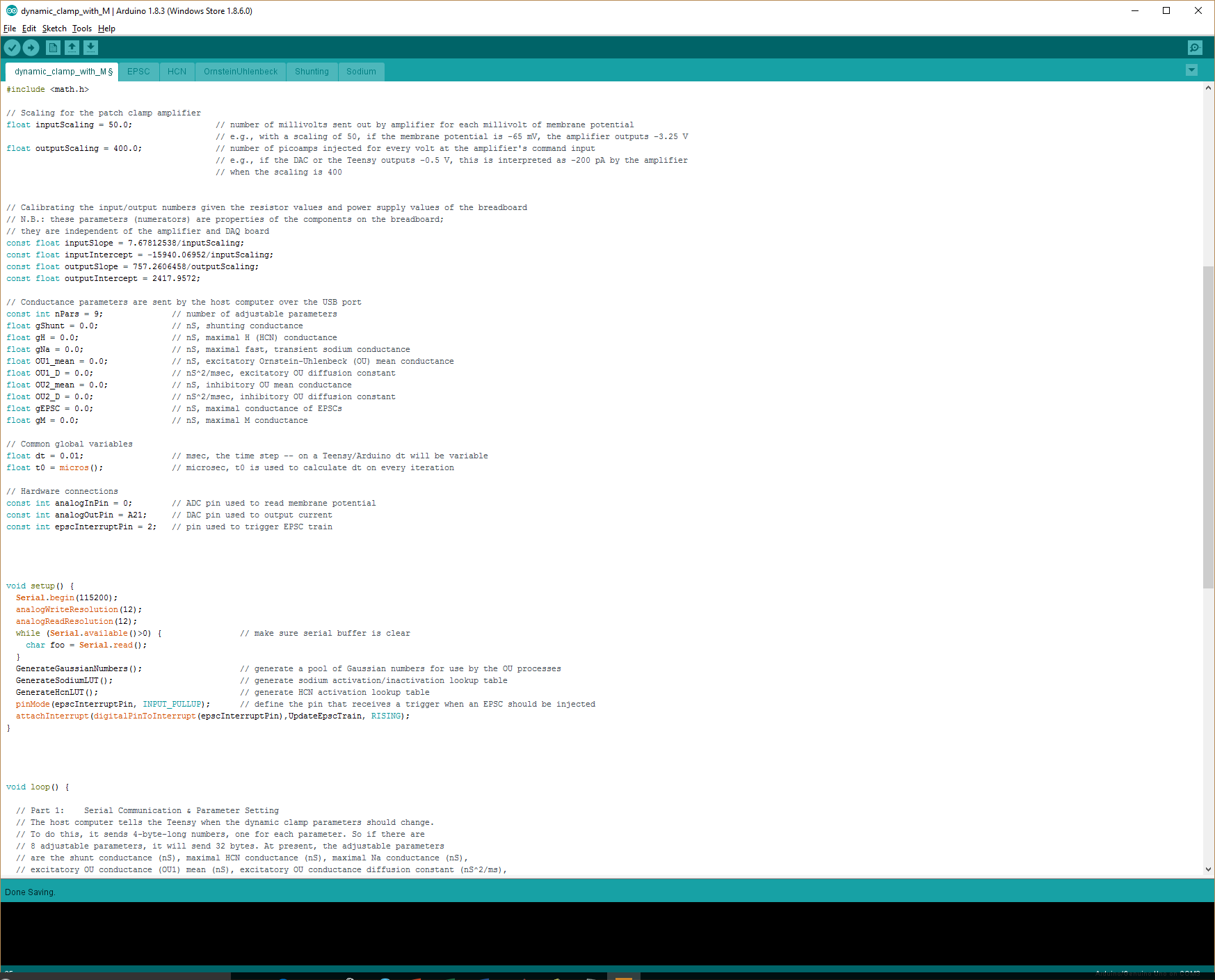


Note that we both changed the number of parameters (nPars) to 9 and added the last line declaring $g_{M}$ and setting its initial value to 0.0

(2) In the *setup()* function, add a call to a function *GenerateMLUT()* that will create lookup tables of values of $m_{inf}$ and $\tau_{m}$ for a wide range of membrane potentials. Rather than doing the calculations at every time step, we will simply look up the appropriate values for the measured $V_{m}$, thus allowing the program to execute more quickly.

The function *GenerateMLUT()* will be defined in the tabbed file we will create shortly.


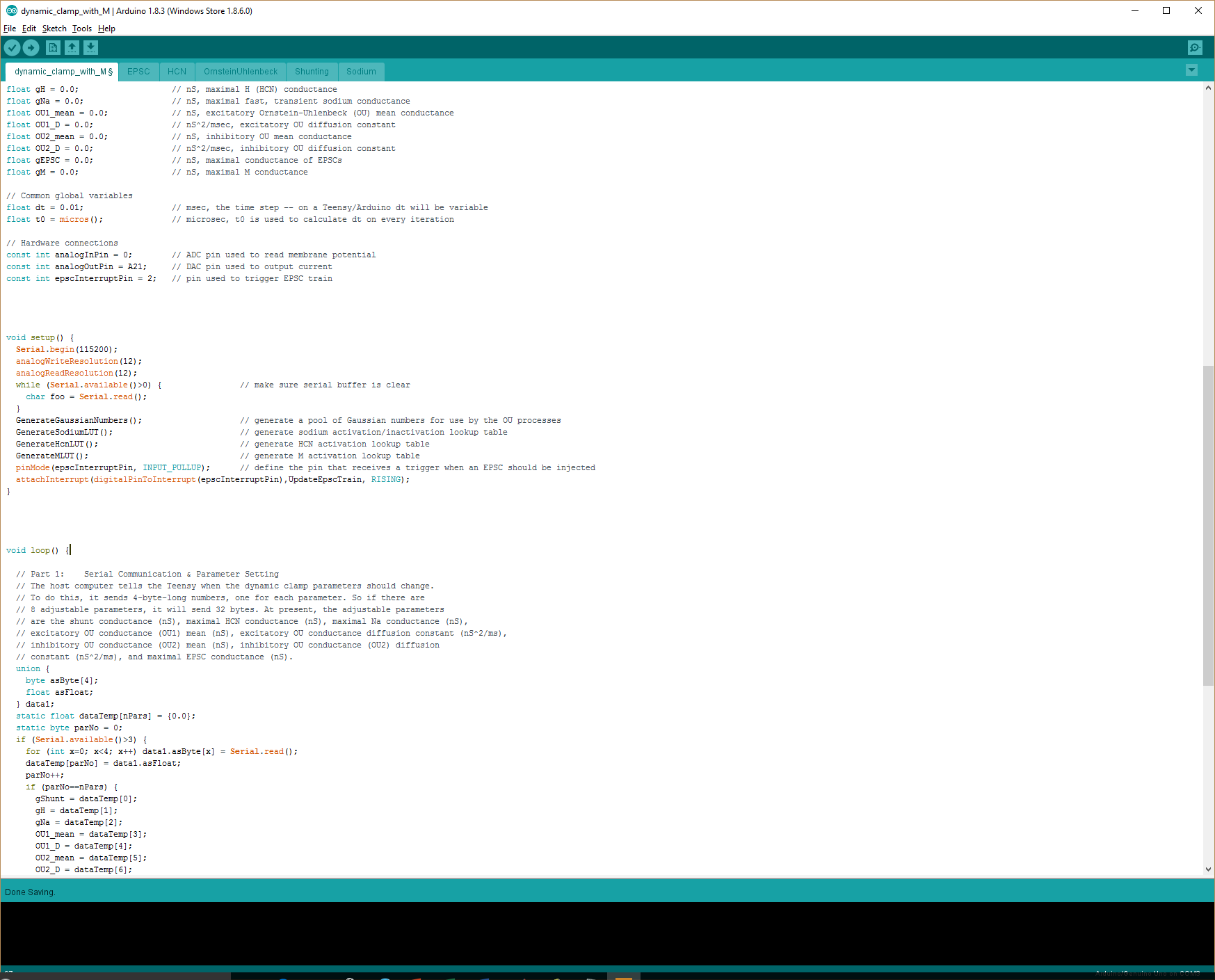


(3) Add the maximal M conductance $g_{M}$ to the list of parameters the Teensy receives over the USB port from the Processing sketch.


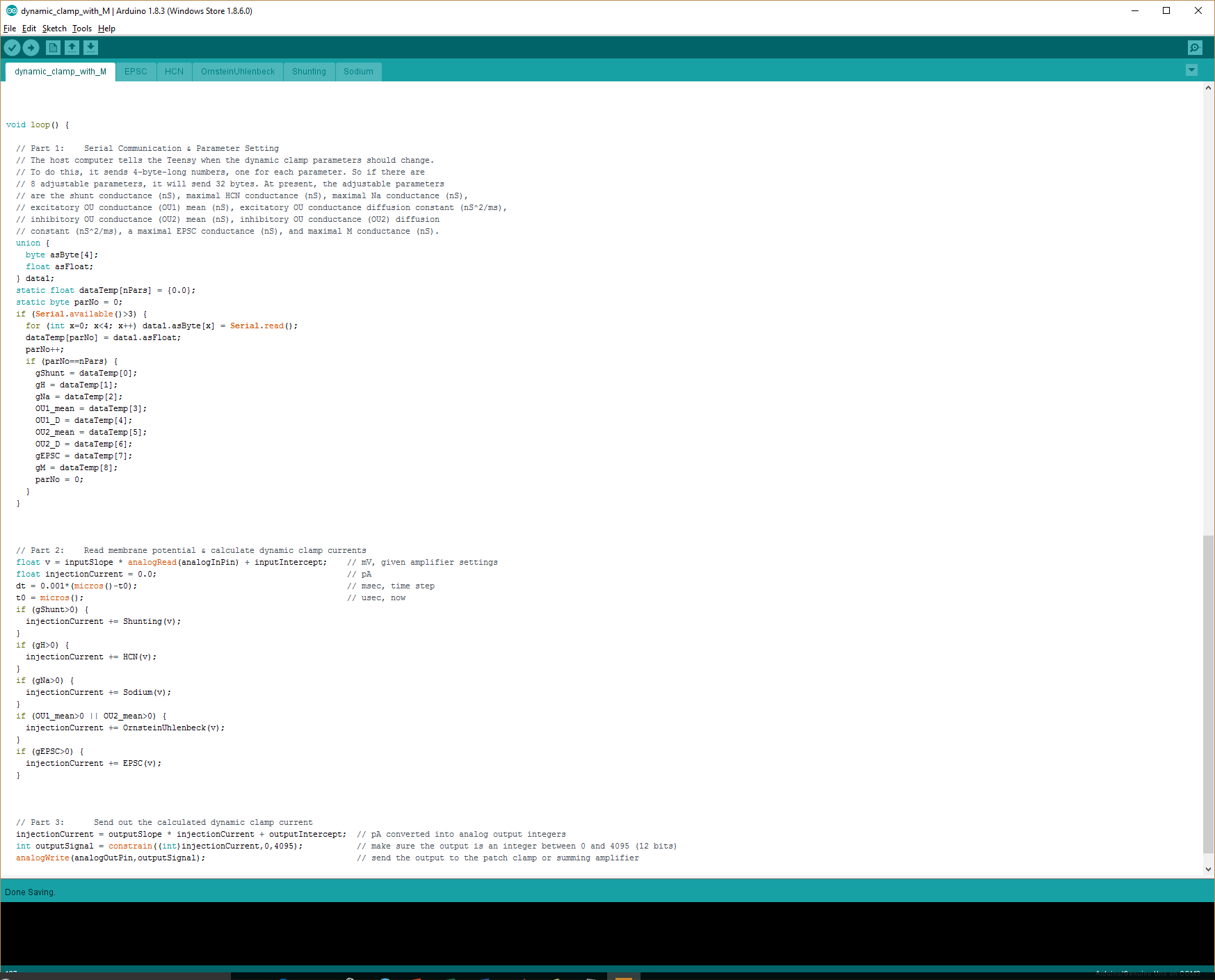


(4) If $g_{M}$>0, have the simulated M current added to the other simulated currents. If it equals 0, then we don’t bother since the current will simply be zero.


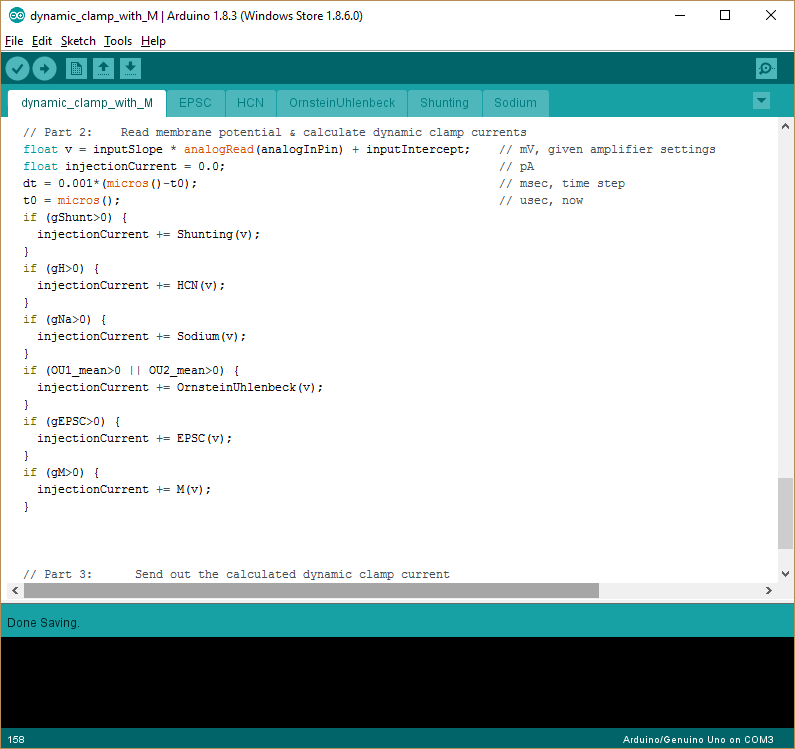


*Tabbed file*

Now we create a tabbed file that defines the M conductance.


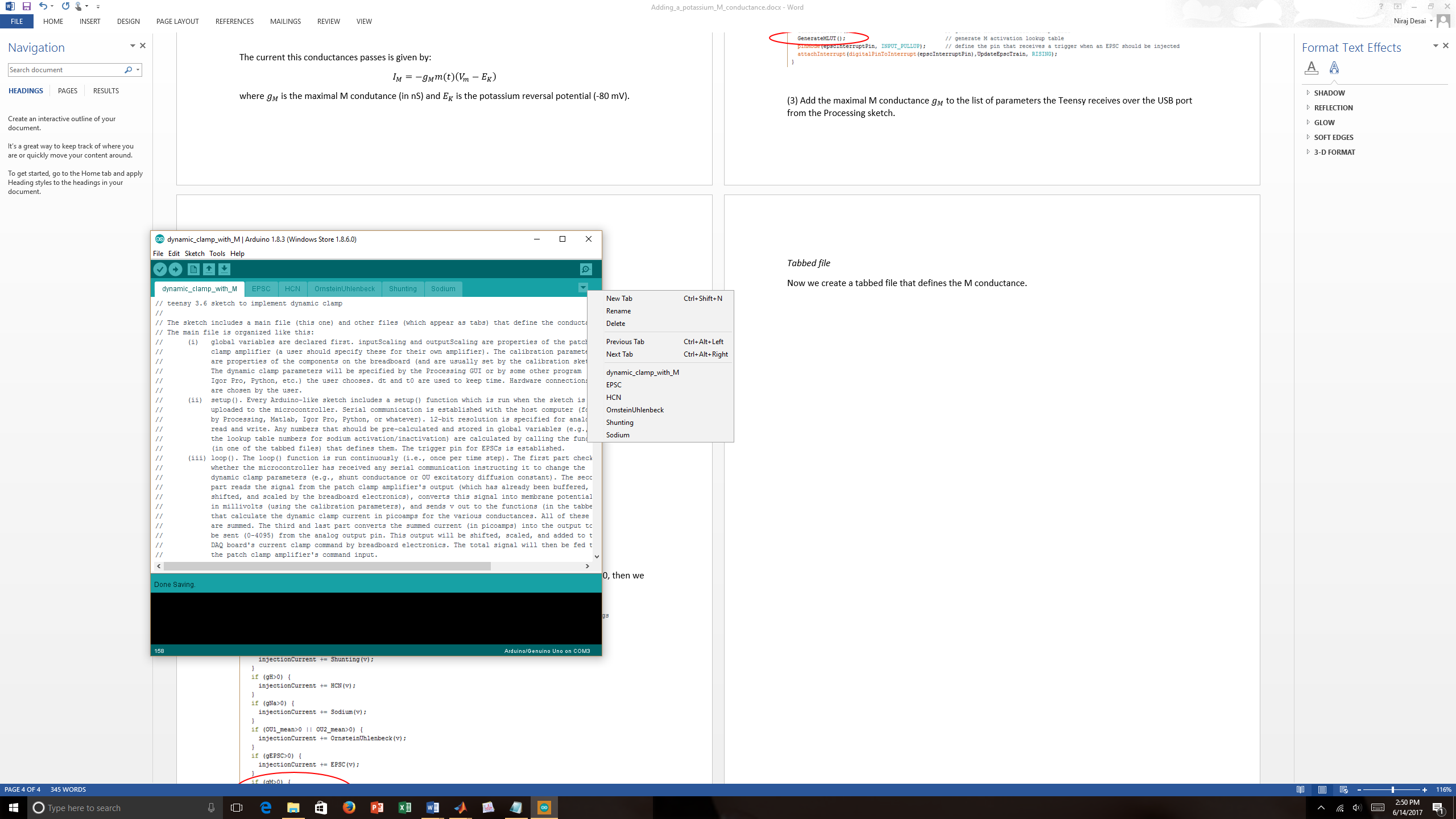


(1) Create a new file. In the Arduino window, look for the downward-facing arrow (indicated by the red arrow). Click it and select “New Tab”. You will then be asked to name the new tabbed file – just call it “M”.

(2) In that M file, enter the three parts shown below: (i) array declaration in which the lookup table values of $m_{inf}$ and $\tau_{m}$ will be stored; (ii) the function *GenerateMLUT()* that generates the lookup tables (for membrane potentials between -100 mV and +50 mV); and (iii) the function that numerically integrates $m(t)$ and calculates the M current at every time step.


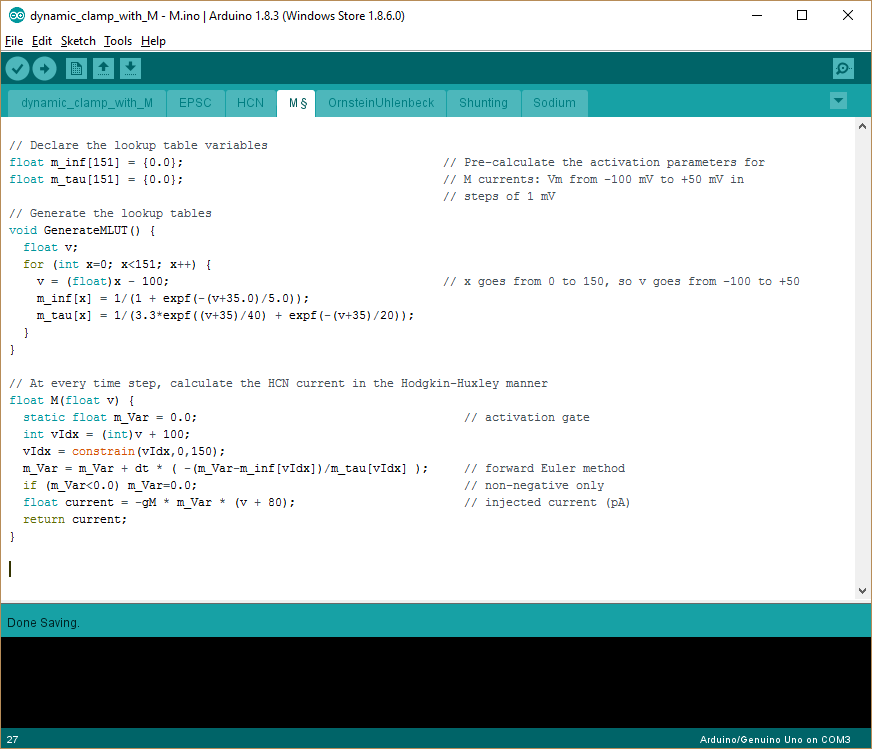


A few things to note. (1) We use the function *expf()* in preference to *exp()* because the former instructs the compiler to make use of Teensy’s floating point unit (FPU), thus speeding up the calculations. (2) We use the built-in function *constrain()* and an *if* statement to make sure vIdx and m_Var stay within bounds. These lines aren’t strictly necessary because an out-of-bounds situation may never arise, but better safe than sorry. (3) We use the forward Euler method to integrate $m(t)$, which is called *m_Var* in the code. The time step is short enough (compared to the kinetics of the M conductance) that this is acceptable.

Finally, save the modified program and upload it to the microcontroller by pressing the right-facing arrow at upper left.

**III. Modifying the Processing program**

To modify the Processing program (*processing_control.pde*) so that it will specify the maximal M conductance $g_{M}$ in addition to the other parameters it already specifies, we need only add four lines.

(1) Add $g_{M}$ to the list of variables declared when the GUI opens.

(2) Add a slider with which to specify the value of $g_{M}$.

(3) Add a statement to write the value of $g_{M}$ to the Teensy microcontroller through the USB port.

(4) Add $g_{M}$ to the list of variables zeroed out when the user presses the ***Zero*** button.


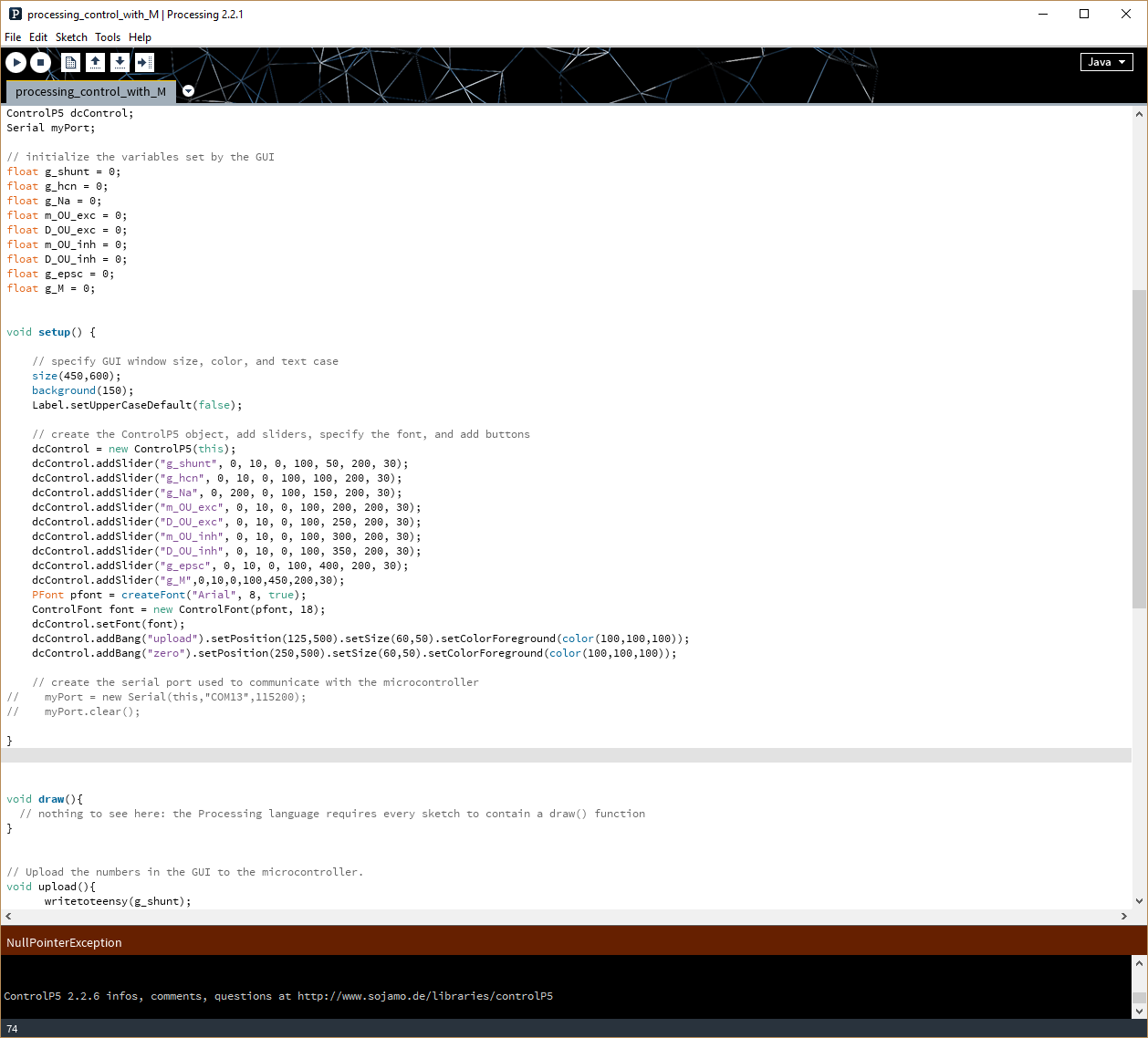


**1**


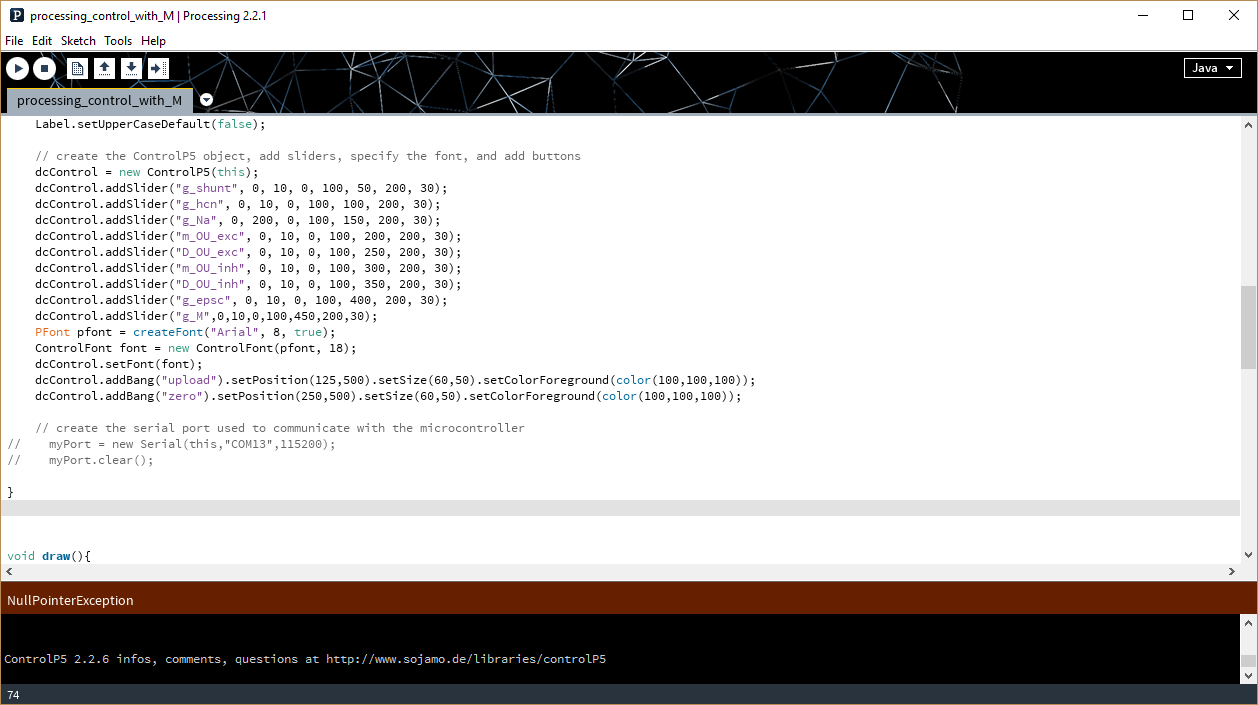


**2**


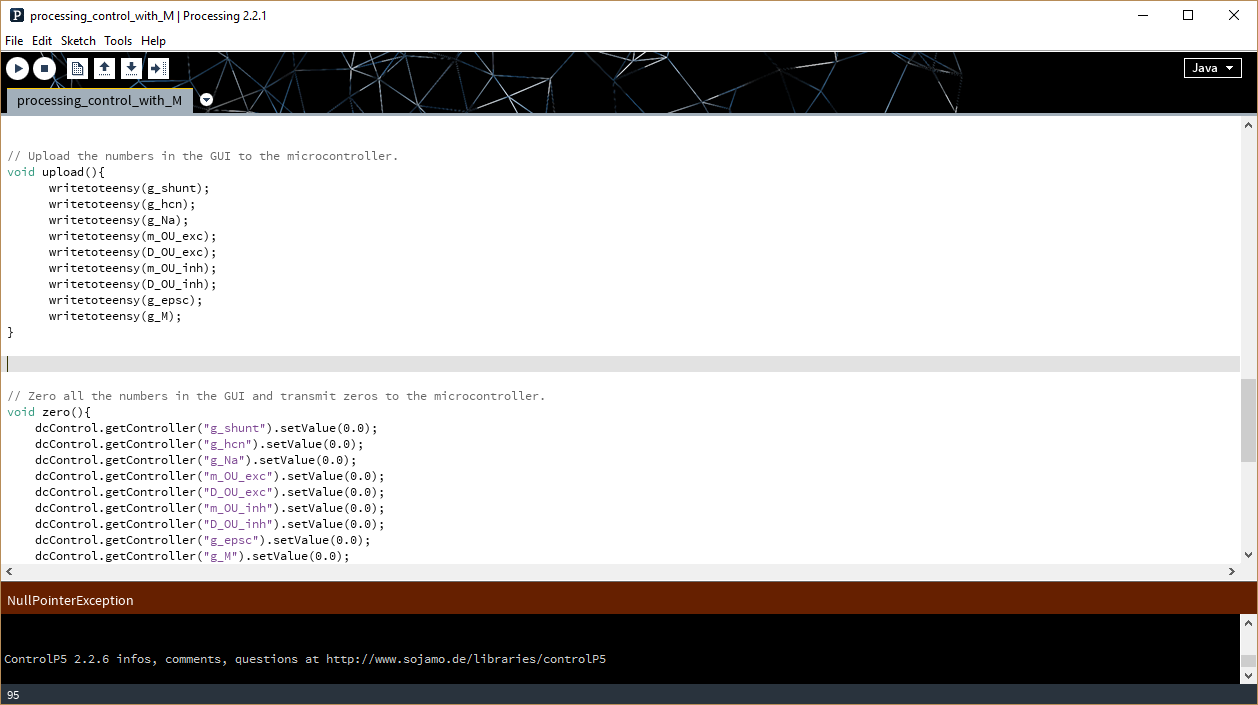


**3**


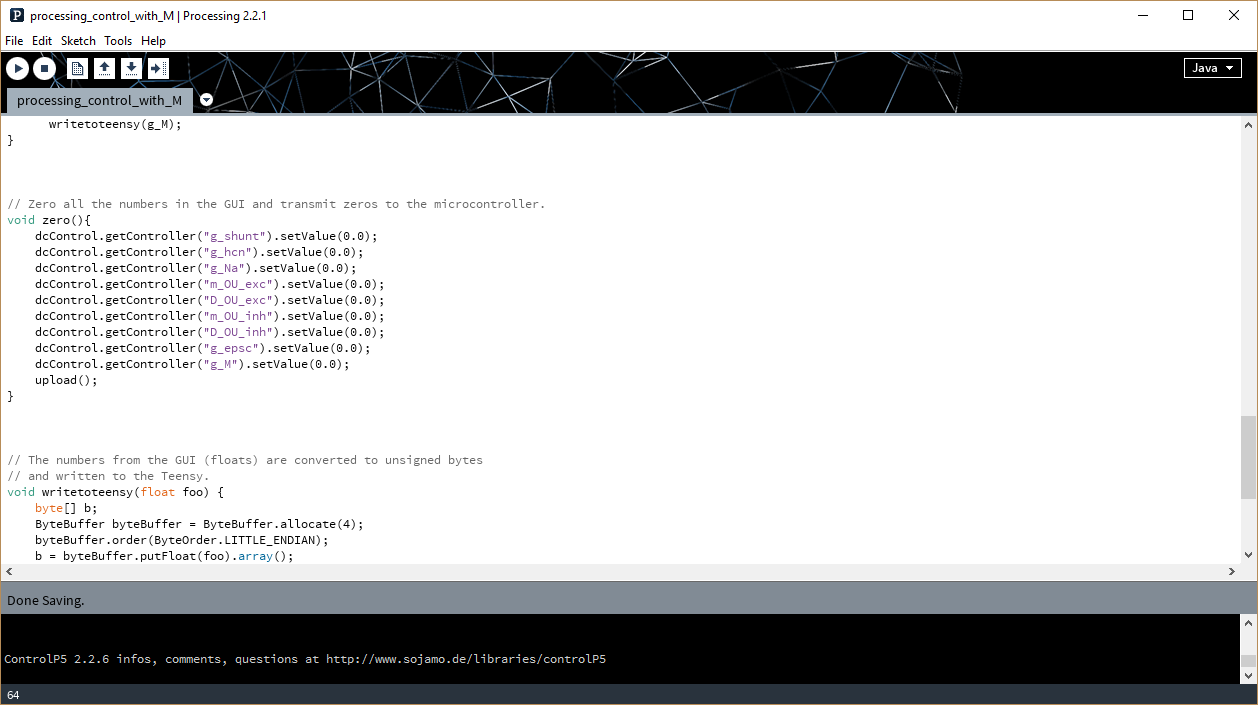


**4**

Save the changes and then run the Processing program. It should open up a GUI window that looks like this:


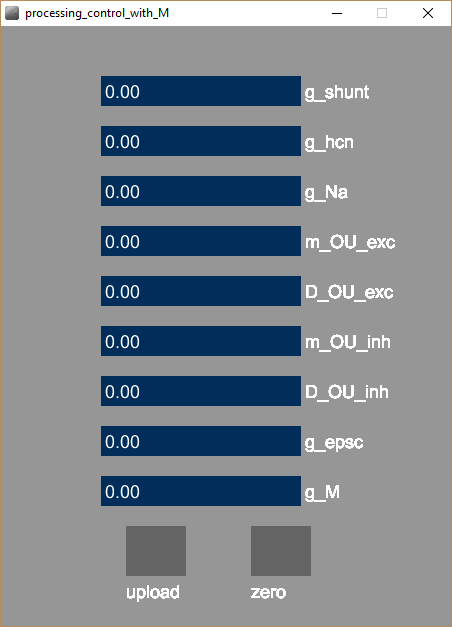
Notice that a slider for the M conductance has been added to the end. It can be used just as the others are.

The completed Arduino and Processing programs, with the changes specified in this document, can be found in the folders called *dynamic_clamp_with_M* and *processing_control_with­_M*.
