# Supplementary material for: A Dynamic Clamp on Every Rig
Source: eNeuro. 2017 Oct 23;4(5):ENEURO.0250-17.2017. doi: 10.1523/ENEURO.0250-17.2017 (PMC5659377; doi:10.1523/ENEURO.0250-17.2017)
Supplement: Extended Data 1 [file enu005172417so1.zip › Extended_data/7_Matlab_alternative_to_Processing/Matlab_alternative.docx]

**Matlab alternative to Processing**

We used the open-source environment Processing ([www.processing.org](http://www.processing.org)) to control the Teensy microcontroller. Processing is useful because it is free and platform independent. However, many alternatives exist and these may be especially attractive to researchers who use other programs to acquire patch clamp data. All that is required is that the program can send a list of numbers to the microcontroller through the USB port.

One possibility is Matlab, which has an extensive user base in the neuroscience community and for which free electrophysiology software is available (e.g., wavesurfer.janelia.org or clm.utexas.edu/robotpatch).

In our example software, the Teensy microcontroller waits to receive eight numbers through the USB port. The eight numbers represent the values of (1) shunt conductance (g_shunt in nS), (2) HCN conductance (g_HCN in nS), (3) sodium conductance (g_Na in nS), (4) excitatory Ornstein-Uhlenbeck (OU) mean conductance (m_OU_exc in nS), (5) excitatory OU diffusion constant (D_OU_exc in nS^2^/ms), (6) inhibitory Ornstein-Uhlenbeck (OU) mean conductance (m_OU_inh in nS), (7) inhibitory OU diffusion constant (D_OU_inh in nS^2^/ms), and (8) EPSC conductance (g_EPSC in nS). When the microcontroller receives these numbers, it changes the value of the conductances and diffusion constants appropriately.

Each number must be a byte array representation of single precision float point (4 bytes containing 32 bits). What this means is described here (<https://en.wikipedia.org/wiki/Single-precision_floating-point_format>). This topic can be somewhat arcane, but fortunately most languages have built-in methods to convert numbers from one type to another. For example, in Matlab, we can convert the number *pi* into a byte array using the function *typecast* like this:

>> pi = 3.141592653589793; % 64-bit representation

>> pi_single_precision = single(pi) % 32-bit representation

pi_single_precision =

3.1415927

>> pi_byte_array = typecast(pi_single_precision,'uint8') % byte array representation

pi_byte_array =

219 15 73 64

One can also go the other way: to convert the byte array back into a (32-bit) floating point, enter this line at the command prompt: typecast(pi_byte_array, ’single’).

In the Matlab script *Matlab_simple_example.m*, we show how to write eight conductance/constant numbers to the Teensy. Before using this script, users should change the serial port name (e.g., ‘COM3’) to the name of the USB port to which their Teensy is attached.

We also provide a Matlab graphical user interface (GUI) with which users can control the board. It has the same functionality as the Processing GUI. To use it, (1) save the two files (*DC.m* and *DC.fig*) in the Matlab current directory; (2) open *DC.m* and find the line “initializeteensy(‘COM3’)”; (3) change COM3 to the name of the USB port for your set-up; (4) save the file; and (5) open the GUI by typing “DC” at the command line. This will open up the GUI, which looks like this:


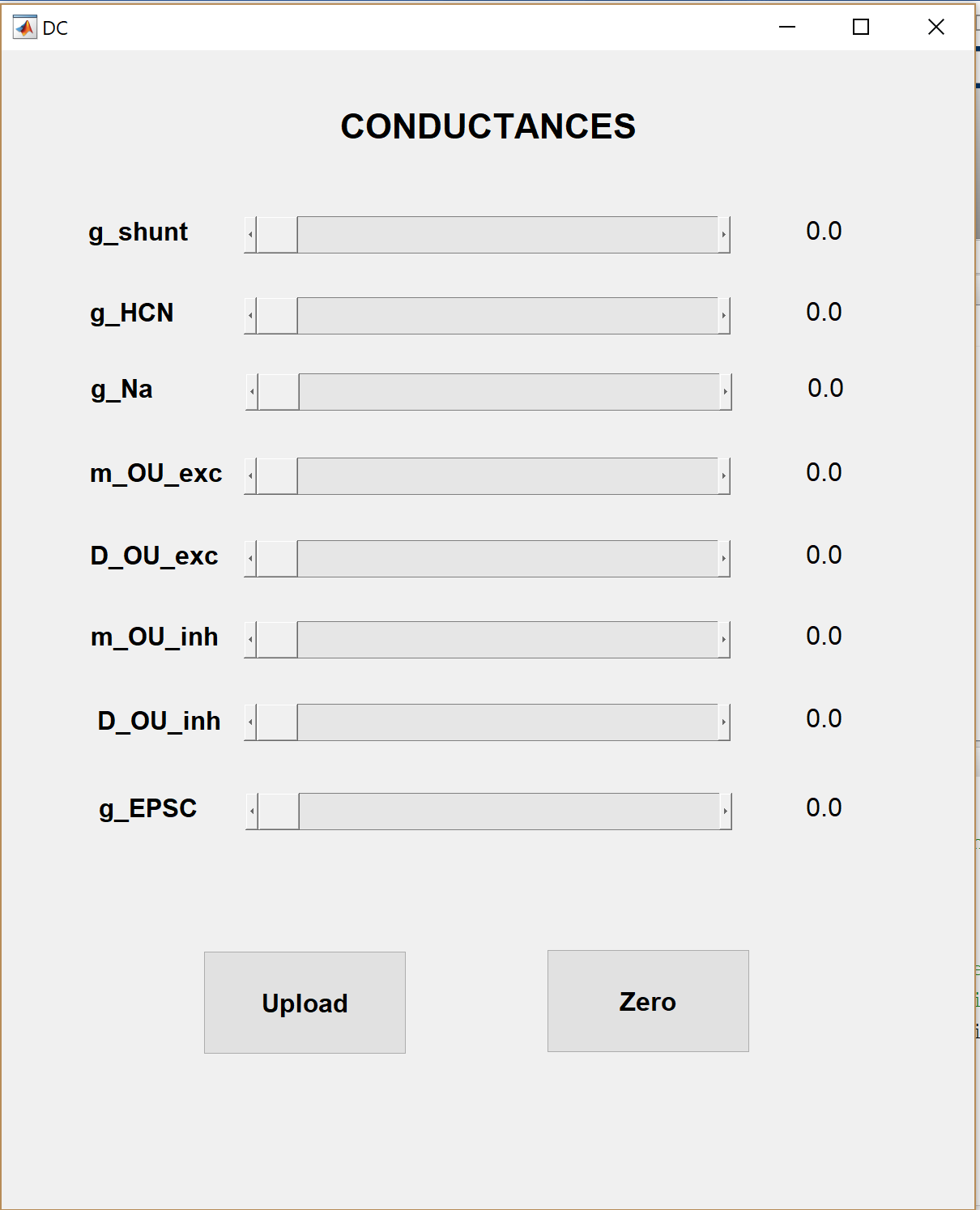


The sliders can be moved to select new values for the conductances and constants. Pressing ***Upload*** sends these values to the Teensy microcontroller. Pressing ***Zero*** zeroes all the values and sends zeros to the microcontroller (i.e., so that no dynamic clamp conductances are being simulated).
