# Supplementary material for: A Dynamic Clamp on Every Rig
Source: eNeuro. 2017 Oct 23;4(5):ENEURO.0250-17.2017. doi: 10.1523/ENEURO.0250-17.2017 (PMC5659377; doi:10.1523/ENEURO.0250-17.2017)
Supplement: Extended Data 1 [file enu005172417so1.zip › Extended_data/8_Calibration_procedure/Calibration_procedure.docx]

**Calibration procedure**

**I. Expected values**

Resistor and capacitor values are inexact and operational amplifiers do not behave ideally. Therefore calculations of the relationship between inputs and outputs will not be quite correct for the electronic circuits we described in Figure 2 of the main text. Even so, it is useful to do those calculations in order to establish baseline behavior. The empirically-determined relationships (once the system is built on a breadboard) will be different but close to the calculated ones if all is well. But if the numbers differ by too much (>5%), that is a sign that something is wrong – e.g., a connection was made between the wrong points or a resistor value is wrong.

So, let’s do the exact (theoretical) calculations.

*Relating V_m_ to the ADC input of the Teensy*

In the main text, we noted that the voltage input to the circuit of Figure 2B is related to the voltage output like this:

$$V_{OUT}=(1+ \frac{R_{6}}{R_{5}})\left[ \frac{R_{4}R_{2}}{\left( R_{3}+R_{4} \right)\left( R_{1}+R_{2} \right)}V_{IN}+9\frac{R_{3}}{\left( R_{3}+R_{4} \right)} \right]$$

With the resistor values of the caption to that figure (R_1_=2200 Ω, R_2_=470 Ω, R_3_=4700 Ω, R_4_=22000 Ω, R_5_=10000 Ω, and R_6_=100 Ω), this becomes:

$$V_{OUT}=0.1465 V_{IN}+1.6001$$

Now, $V_{OUT}$ is not read directly by the Teensy microcontroller. Rather, for a given voltage, the ADC input to the microcontroller equals (4095/3.3)*voltage. That is, the ADC input maps voltages from 0 to 3.3 V onto the range 0 to 4095. (Note: 12 bits = 2^12 = 4096. There are 4096 distinct integer levels between 0 and 4095.) So, in terms of the ADC input, the equation is:

$$ADC=181.7855V_{IN}+1985.5940$$

Inverting this equation, we have:

$$V_{IN}=0.0055 ADC-10.9227$$

Now, $V_{IN}$ is the voltage (in volts) sent out by the patch clamp amplifier. If the amplifier is set so that its input gain is ${gain}_{INPUT}$ (the number of millivolts sent out for every millivolt of membrane potential read in), then the relationship between the ADC reading and the membrane potential $V_{m}$ (in mV) is this:

$$V_{m}= \left( \frac{5.5010}{{gain}_{INPUT}} \right) ADC+\left( \frac{-10920.27}{{gain}_{INPUT}} \right)$$

*Relating I to the DAC output of the Teensy*

The dynamic clamp simulation specifies what current $I_{DC}$ (in pA) should be injected into the neuron, but the signal to the DAC output must be an integer between 0 and 4095 (12 bits) representing voltages between 0 and 3.3 V. We have to map one representation onto the other.

The amplifier receives a voltage command and interprets this number as a current depending on its output gain, ${gain}_{OUTPUT}$:

$$I_{DC}={gain}_{OUTPUT}*V_{DC}$$

where $I_{DC}$ and $V_{DC}$ are the dynamic clamp current and command voltage, respectively. They are defined in Figs. 1B and 2D. As we noted in the main text, the command voltage $V_{DC}$ is related to the voltage from the Teensy, $V_{DAC}$, like this:

$$V_{DC}=\left( 1+\frac{R_{10}}{R_{9}} \right)\left[ \frac{R_{8}}{\left( R_{7}+R_{8} \right)}V_{DAC}-9\frac{R_{7}}{\left( R_{7}+R_{8} \right)} \right]$$

Combining the two equations and using the resistor values in the caption of Fig. 2, we have the following relationship between current $I_{DC}$ (in pA) and the DAC voltage output $V_{DAC}$ (in V):

$$\frac{I_{DC}}{{gain}_{OUTPUT}}=2.459 V_{DAC}-4.8689$$

$V_{DAC}$ (in V) is related to the DAC output (0-4095) in this way: $V_{DAC}=\frac{3.3}{4095}DAC$. Finally, we have the relationship between the current the dynamic clamp system specifies $I_{DC}$ and the DAC output of the Teensy:

$$DAC= \frac{504.6777}{{gain}_{OUTPUT}}I_{DC}+2457.016$$

*Putting the calibration numbers into the Arduino program*

We can use the two boxed equations to add calibration numbers to the main file of the Arduino program. On our system (Multiclamp 700B, 500 MΩ feedback resistor, with the current clamp gain set at 5), the input gain ${gain}_{INPUT}$ is 50 mV/mV and the output gain ${gain}_{OUTPUT}$ is 400 pA/V. So, we will fill out the calibration section of the main file in this way:


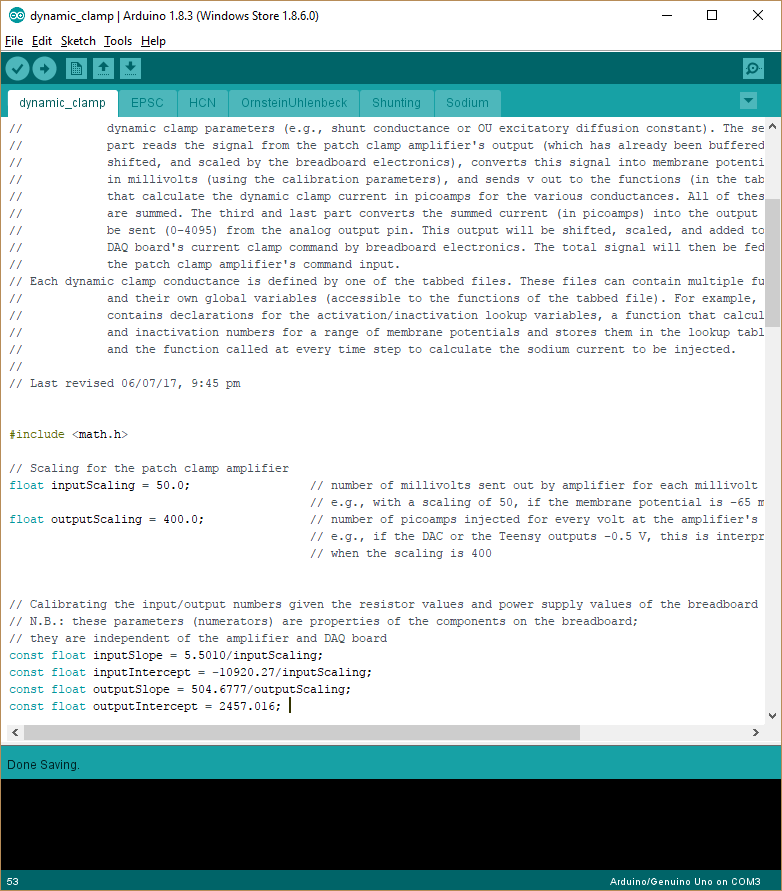


**II. Calibration program**

Even with high quality components, the calibration numbers we have just calculated will be slightly off. Therefore, it is better to measure the input and output of the dynamic clamp system directly and use those numbers instead.

*Direct method*

The most direct way of calibrating the system exactly is to use one analog input and one analog output of your DAQ system (e.g., National Instruments board or ITC-18). Connect the analog output to the input ($V_{IN}$) of Fig. 2B and the analog input to pin A21 (DAC0) of the Teensy.

To get the input scaling numbers, upload the Arduino sketch *passthrough* contained in this folder to the Teensy microcontroller. It reads the input to the Teensy and simply writes the same number to the output. Now vary the output voltage from your DAQ system across some broad range (e.g., ±5 V) and measure the Teensy voltage at the input. The relationship between the two should be strongly linear (as in Fig. 3A) and by fitting a straight line you can extract the inputSlope and inputIntercept numbers. Be careful to convert the Teensy voltage $V_{OUT}$, which is in volts, into the ADC number, which is an integer between 0 and 4095, as mentioned above: $ADC=\frac{4095}{3.3}* V_{OUT}$.

To get the output scaling numbers, upload the Arduino sketch *specifyoutput* contained in this folder to the Teensy microcontroller. This time connect your DAQ system’s analog input so that it measures $V_{DC}$ of Fig. 2D. You can send the Teensy a number between 0-4095 over the USB port and it will output that number from its DAC port. Again, the relationship between the DAC numbers and $V_{DC}$ will be strongly linear (Fig. 3A). Convert $V_{DC}$ into $I_{DC}$, using $I_{DC}= {gain}_{OUTPUT}* V_{DC}$, and fit a straight line with $I_{DC}$ as the x variable and the DAC numbers as the y variable. The slope and intercept will equal outputSlope and outputIntercept of the main Arduino program.

These calibration numbers inputSlope, inputIntercept, outputSlope, and outputIntercept should not differ from the calculated values by any more than 5%. Comparing the two sets of numbers is a good way to make sure the circuit on the board was assembled correctly.

*Model cell method*

A less direct but simpler method is to attach a model cell to the amplifier’s headstage and use the Arduino program (*teensy_calibration.ino*) and the Processing program (*processing_calibration.pde*) provided in this folder.

In general the equivalent circuit of the model cell will look something like this:


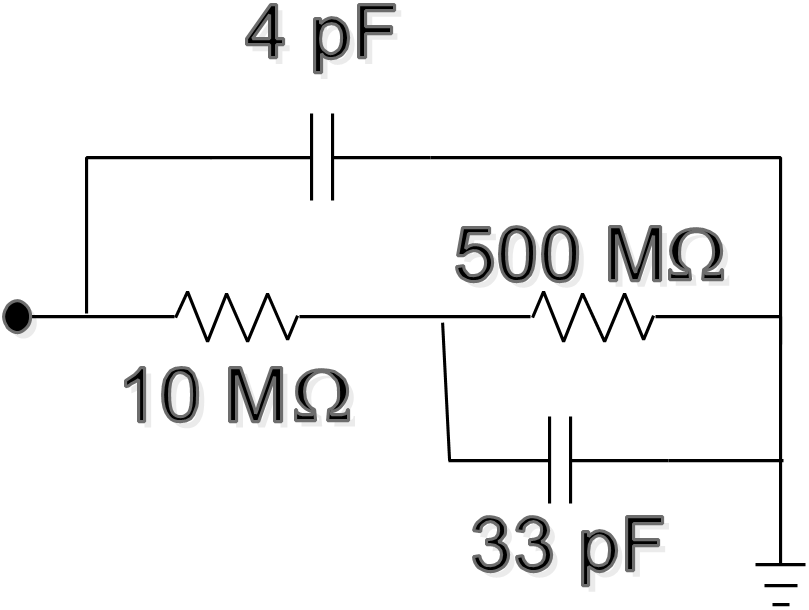


This is the Patch-1U model cell (“cell mode”) from Molecular Devices. When subjected to a constant current $I_{0}$, it should exhibit a steady-state voltage deflection of $\Delta V_{SS}=I_{0}*510 MΩ$. (Note that this depends only on the resistors; in the steady state, the capacitors have dropped out.) In practice, because of imperfections in the components that make up the model cell (see, e.g., <http://bit.ly/2qHavi1>) the total resistance will be somewhat different from 510 MΩ. But this can be calculated directly – just as one would calculate input resistance during a whole cell recording. You should do so for your own model cell.

(1) Having gotten this number, put it in the Processing sketch:


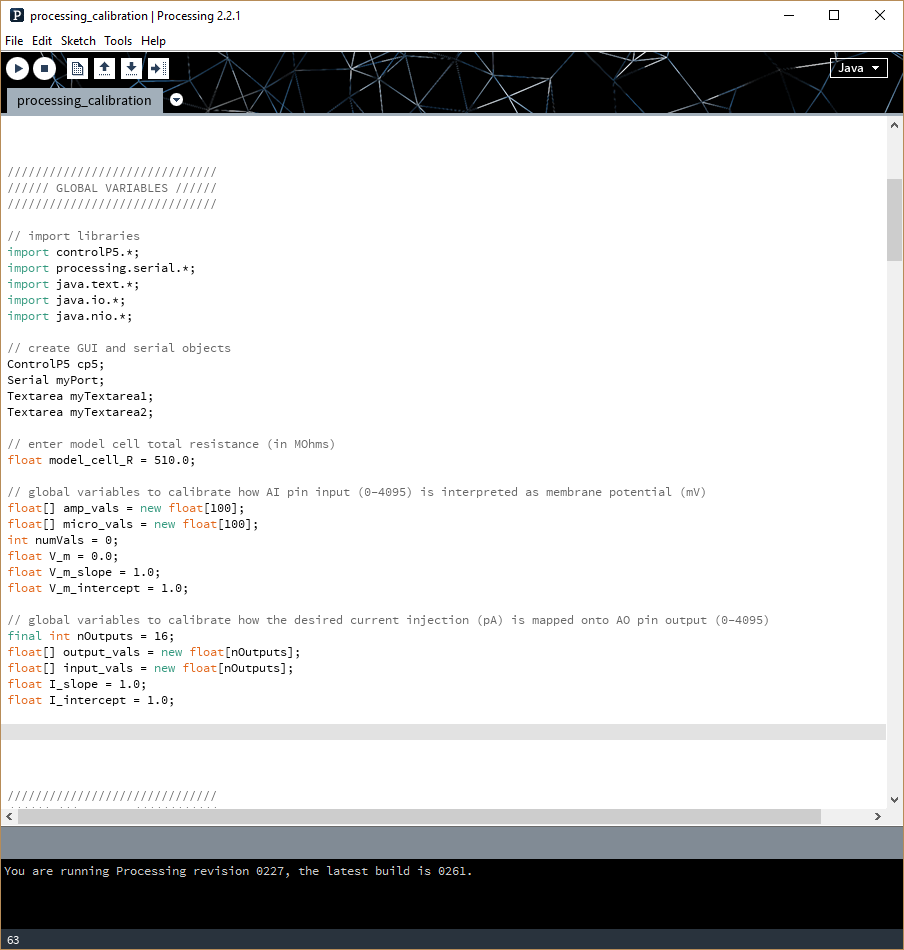


(2) Enter the name of the USB port to which the Teensy microcontroller is attached in the Processing program in its *setup()* function. The default is “COM3”.


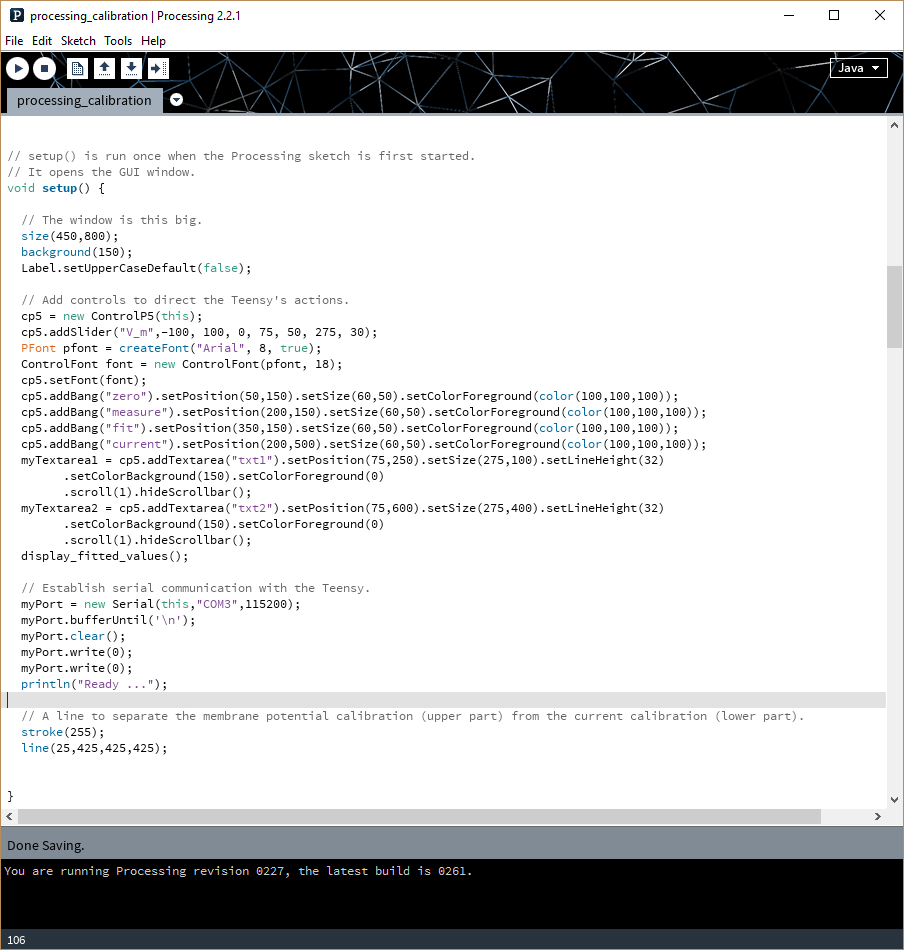


(3) Upload the Arduino program to the Teensy microcontroller.

(4) Run the Processing program by pressing the right-facing arrow at the upper right of the Processing window. This will open up the following GUI:


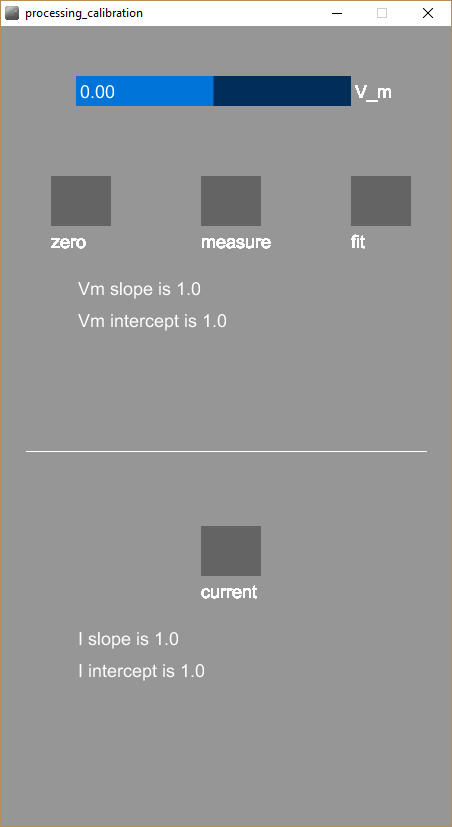


The top half concerns calibration of the Teensy’s input (how it relates a given ADC reading with a membrane potential $V_{m}$). The bottom half concern’s calibration of the Teensy’s output (how it relates a desired dynamic clamp current $I_{DC}$ with an output signal to send to the Teensy’s DAC output). In other words, the top half calculates the values of inputSlope and inputIntercept, and the bottom half calculates the values of outputSlope and outputIntercept.

First, we run the top half of the GUI, thus fixing inputSlope and inputIntercept. Then, we run the bottom half, fixing outputSlope and outputIntercept. The order matters: we need the Teensy to be able to take a good measurement of $V_{m}$ in order to get the output parameters right.

(5) Change your patch clamp amplifier to I=0 mode. Use the amplifier’s offset control to set the $V_{m}$ reading to 0 mV. Use the slider on the Processing GUI to set the V_m to 0 as well. Press the ***measure*** button. Processing has now read a value from the Teensy’s ADC input and associated it with $V_{m}$= 0 mV.

(6) Repeat this procedure for 5-10 values of $V_{m}$ between -80 mV and +50 mV. If you make a mistake and want to start over, press ***zero***; this clears all the saved values.

(7) Press the ***fit*** button. Processing will fit a straight line using the saved values of amplifier $V_{m}$ and the microcontroller ADC input. The slope and intercept will be displayed in the GUI window. You can use these to set the values of inputSlope and inputIntercept in the main Arduino dynamic clamp program, as we did at the end of the ***Expected values*** section above. Remember to adjust the values based on the ${gain}_{INPUT}$ parameters of yoru amplifier.

Pressing ***fit*** also saves the values used in the fitting to a text file saved to the same folder as the *processing_calbration.pde* file. It will have a name like Vm_YYYY_MM_DD_hh_mm_ss.txt, where YYYY is the year, MM is the mohth, DD is the day, hh is the hour, mm is the minue, and ss is the second. The first column will be the $V_{m}$ readings, the second column will be the ADC readings. The two columns will be separated by commas and the numbers can be loaded, for example, into Excel in case you wish to check Processing’s calculations.

***ALTERNATIVE: You can do parts 5-7 in I-clamp mode. Start with zero injected current and then change*** $\boldsymbol{V}_{\boldsymbol{m}}$ ***by changing the holding (dc) current.***

(8) Having established the inputSlope and inputIntercept numbers, we now can establish the outputSlope and outputIntercept numbers. Switch the amplifier to I-clamp mode. Press the ***current*** button at the bottom of the Processing GUI. Teensy will now set its DAC output to a range of values between 0 and 4095 while recording membrane potential. By diving the latter numbers by the model cell’s resistance, it will convert them into current readings (in pA). It will then fit a straight line and display the values of outputSlope and outputIntercept. Use these to fill out the calibration section of the main Arduino dynamic clamp program, as we did at the end of the ***Expected values*** section above. Again, be mindful to use the value of ${gain}_{INPUT}$ specific to your amplifier.

The numbers used in the fitting will be saved to a text file in the same folder as the *processing_calibration.pde* file. It will have a name like I_YYYY_MM_DD_hh_mm_ss.txt. The first column will be the DAC output, the second column will be the current. They will be separated by commas and the numbers can be imported into Excel and many other programs, so that you can check that there are no problems.

Before ending, it is important to emphasize the importance of comparing any measured calibration numbers (whether direct method or model cell method) to the expected numbers of the exact calculations (section I). While resistors and op-amps are not ideal, even the cheapest ones are not really *that* bad. Well-calibrated numbers should differ from the calculated ones by (much) less than 5%. Any discrepancy larger than this should cause you to double check all the connections and use a multimeter (or the equivalent) to check each component.
