# Supplementary material for: A Dynamic Clamp on Every Rig
Source: eNeuro. 2017 Oct 23;4(5):ENEURO.0250-17.2017. doi: 10.1523/ENEURO.0250-17.2017 (PMC5659377; doi:10.1523/ENEURO.0250-17.2017)
Supplement: Extended Data 1 [file enu005172417so1.zip › Extended_data/9_Alternatives_to_Teensy/Alternatives to Teensy.docx]

**Alternatives to Teensy**

We built the dynamic clamp system around the Teensy 3.6 microcontroller because, compared to alternatives of its class and price point, it is faster and has more memory. However, it is important to note that other microcontrollers could be used in its place. In particular, microcontrollers that can be programmed using the Arduino integrated development environment (IDE) require only small modifications to the code. There are many microcontrollers like this because Arduino has emerged as a standard in the community.

To demonstrate this point, we describe in this document how to use an Arduino Due or a chipKit uC32 in place of the Teensy 3.6. Neither is the equal of the Teensy 3.6 in speed or memory, but both are plausible alternatives.

**I. Arduino Due**

The Arduino line of microcontrollers includes many products ([www.arduino.cc](http://www.arduino.cc)). At this writing (08/26/17), the product best suited to the dynamic clamp technique is the Arduino Due (clock speed 84 MHz, 512 kB flash memory, 92 kB SRAM memory). It includes 12 ADC inputs and 2 DAC outputs.

In the folder *dynamic_clamp_arduino_due*, we have modified the dynamic clamp code to work with the Arduino Due.

*Main file.* The main file differs from that of the Teensy main file in only two ways. (1) The pin connections have different names. The first DAC output of the Due is called “DAC0” whereas that of the Teensy is “A21”. The EPSC trigger pin we use for the Due is number 13 rather than number 2. (2) To keep track of time, we use the built-in Arduino functions *millis()* and *micros()* rather than the data types *elapsedMillis* and *elapsedMicros*. The latter types are also available for the Due but using them requires installing a separate library. Of course, this is simple to do but we chose instead to illustrate how to use *millis()* and *micros()* in place of the elapsed time data types; both methods work with the Teensy 3.6.

*Tabbed (conductance) files.* The Teensy has a floating point unit (FPU). To instruct the compiler to use the FPU, one uses special functions that end in the letter *f* in place of standard functions. One uses *expf*() to calculate an exponential rather than *exp()*; one uses *sinf()* to calculate the sine rather than *sin()*. The Due does not have an FPU. To make the code compatible with the Due, we simply removed the letter *f* – that is, we replaced the special FPU functions with the standard functions.

One other thing to keep in mind when using the Due: although the DAC outputs are nominally 3.3 V, they do not cover the full range between 0 V and 3.3 V. Instead they only cover the range 0.55 V to 2.75 V. The output calibration parameters (slope and intercept) will therefore be different from what one would have calculated using a Teensy.

**II. chipKit uC32**

chipKit is a line of microcontrollers based on Microchip Technology’s PIC32 microcontroller chip ([chipkit.net](http://www.chipkit.net)). Even though it is not an Arduino product, it can still be programmed using the Arduino IDE.

One nice thing about the chipKit uC32 is that there is an add-on called Analog Shield (<http://bit.ly/2wehEpp>) available for it. This add-on allows the uC32 to read and write voltages between -5 V and +5 V directly. This means that users will not need to build the differential amplifier parts of the system (parts 2 and 4 of Fig. 1B of the main text). Of course, this convenience comes at a price ($50 as of this writing).

In the folder *dynamic_clamp_chipKit_uC32*, we have modified the dynamic clamp code to work with the chipKit uC32.

*Main file.* In addition to the two changes that apply to the Arduino Due (pin connections with different names; using *millis() and micros()* instead of the elapsed time data types), the uC32 main file must be modified in other respects to take advantage of the Analog Shield. (1) At the top of the file, we include two necessary libraries (analogShield.h and SPI.h). (2) The functions *analogRead()* and *analogWrite()* are replaced by *analog.read()* and *analog.write*(). (3) The ADC inputs and DAC outputs of the Analog Shield are 16 bit rather than 12 bit. So the calibration numbers are all different from those for the Teensy and the output is constrained to be less than 65536 (= 2^16) rather than less than 4096 (= 2^12).

*Tabbed (conductance) files*. As in the Arduino Due case, we must remove the letter *f* because the uC32 lacks an FPU.
