# Supplementary figures and images for: A Dynamic Clamp on Every Rig
Source: eNeuro. 2017 Oct 23;4(5):ENEURO.0250-17.2017. doi: 10.1523/ENEURO.0250-17.2017 (PMC5659377; doi:10.1523/ENEURO.0250-17.2017)

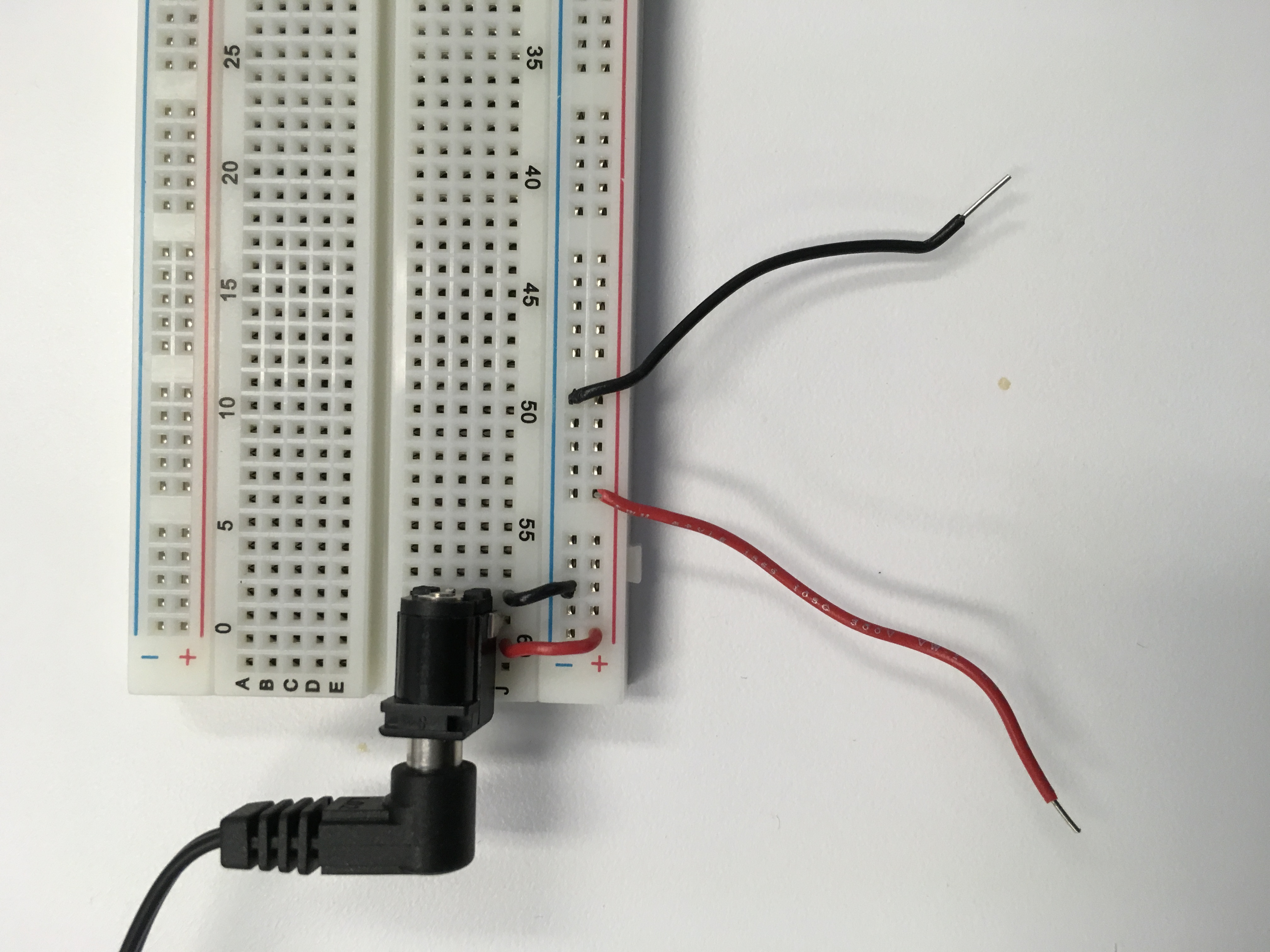

Supplement: Extended Data 1 [file enu005172417so1.zip › Extended_data/2_Assembling_the_system/Higher_quality_images/1_barrel_connector.JPG]

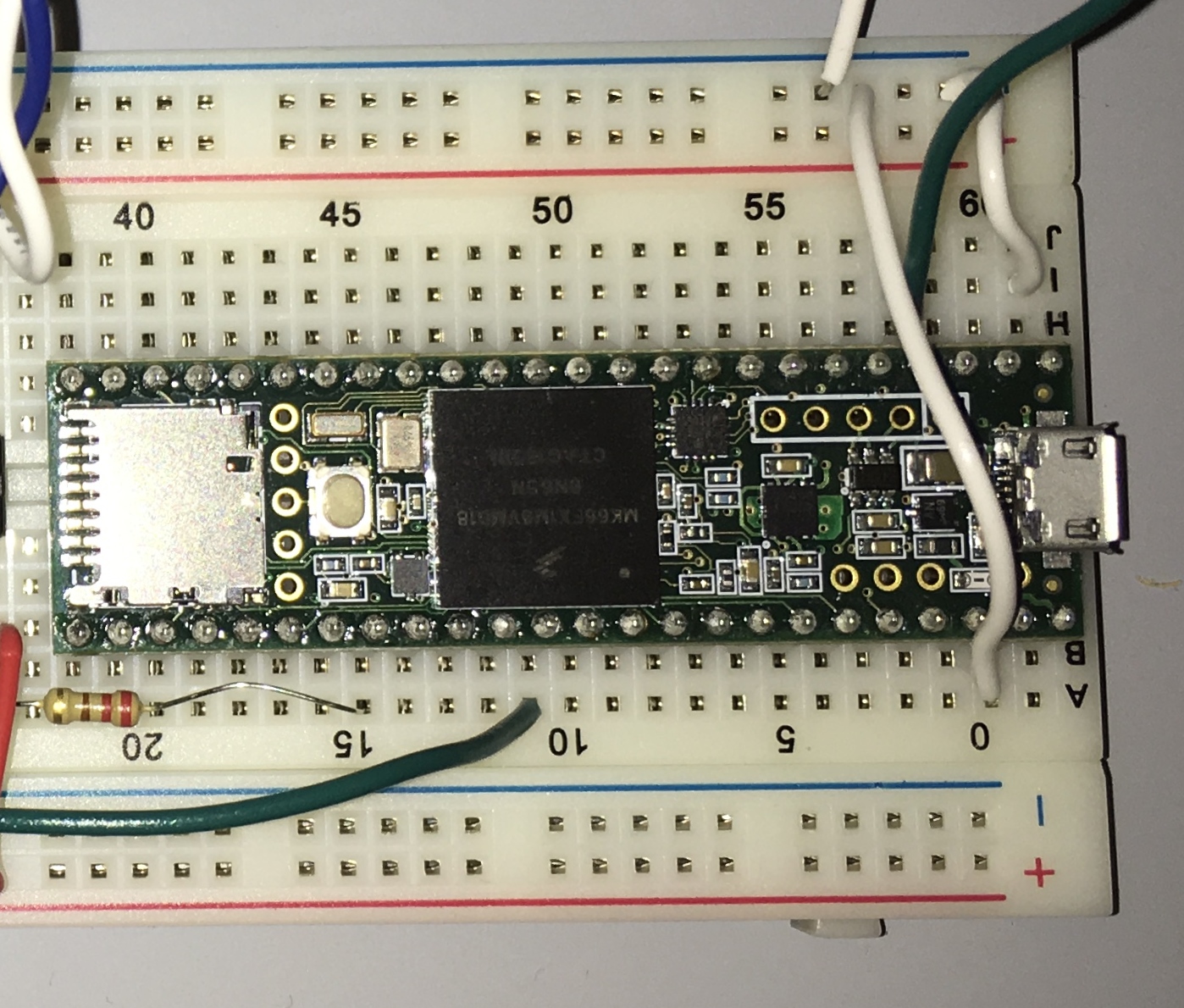

Supplement: Extended Data 1 [file enu005172417so1.zip › Extended_data/2_Assembling_the_system/Higher_quality_images/10_teensy_connections.JPG]

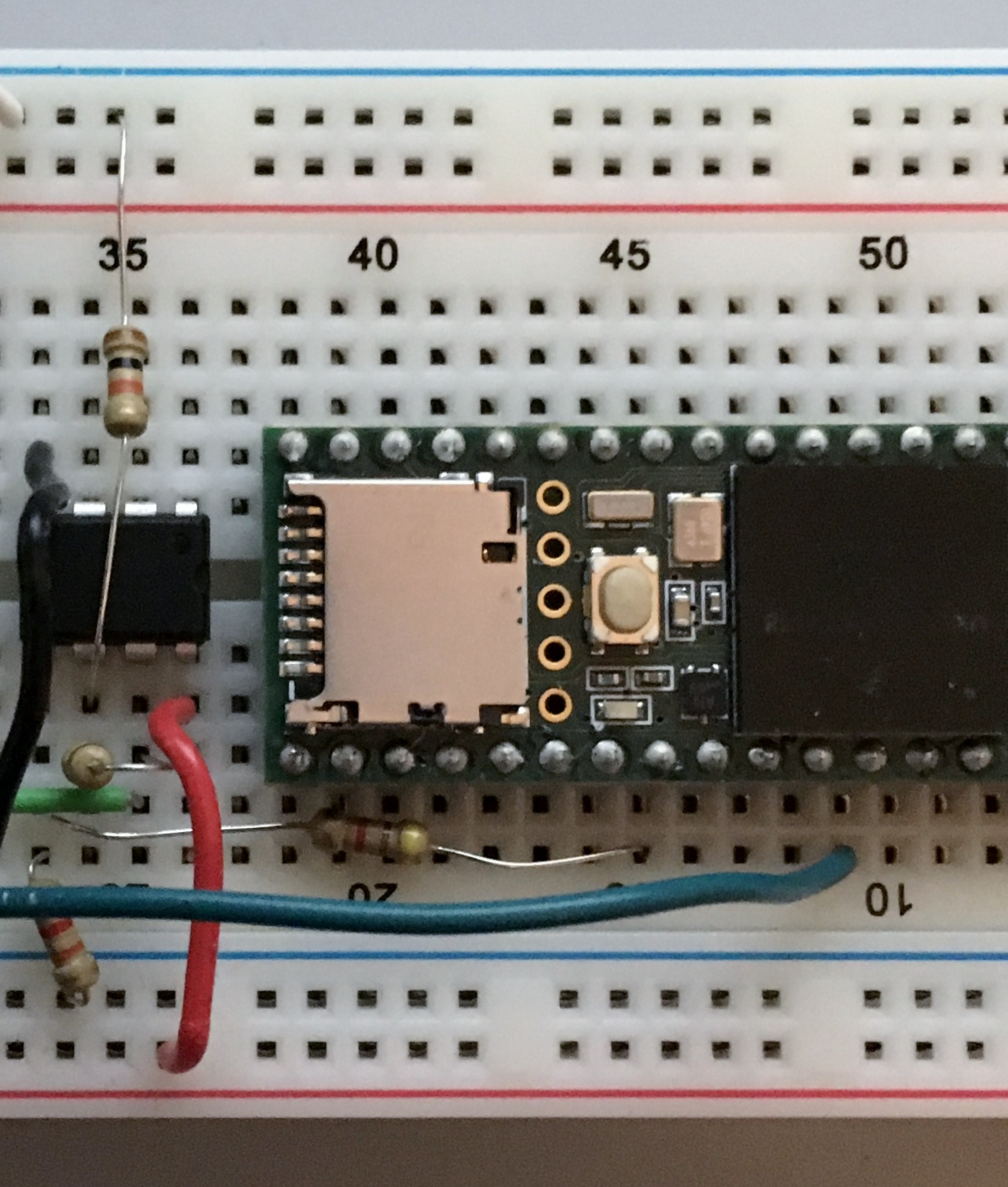

Supplement: Extended Data 1 [file enu005172417so1.zip › Extended_data/2_Assembling_the_system/Higher_quality_images/11_teensy_connections.JPG]

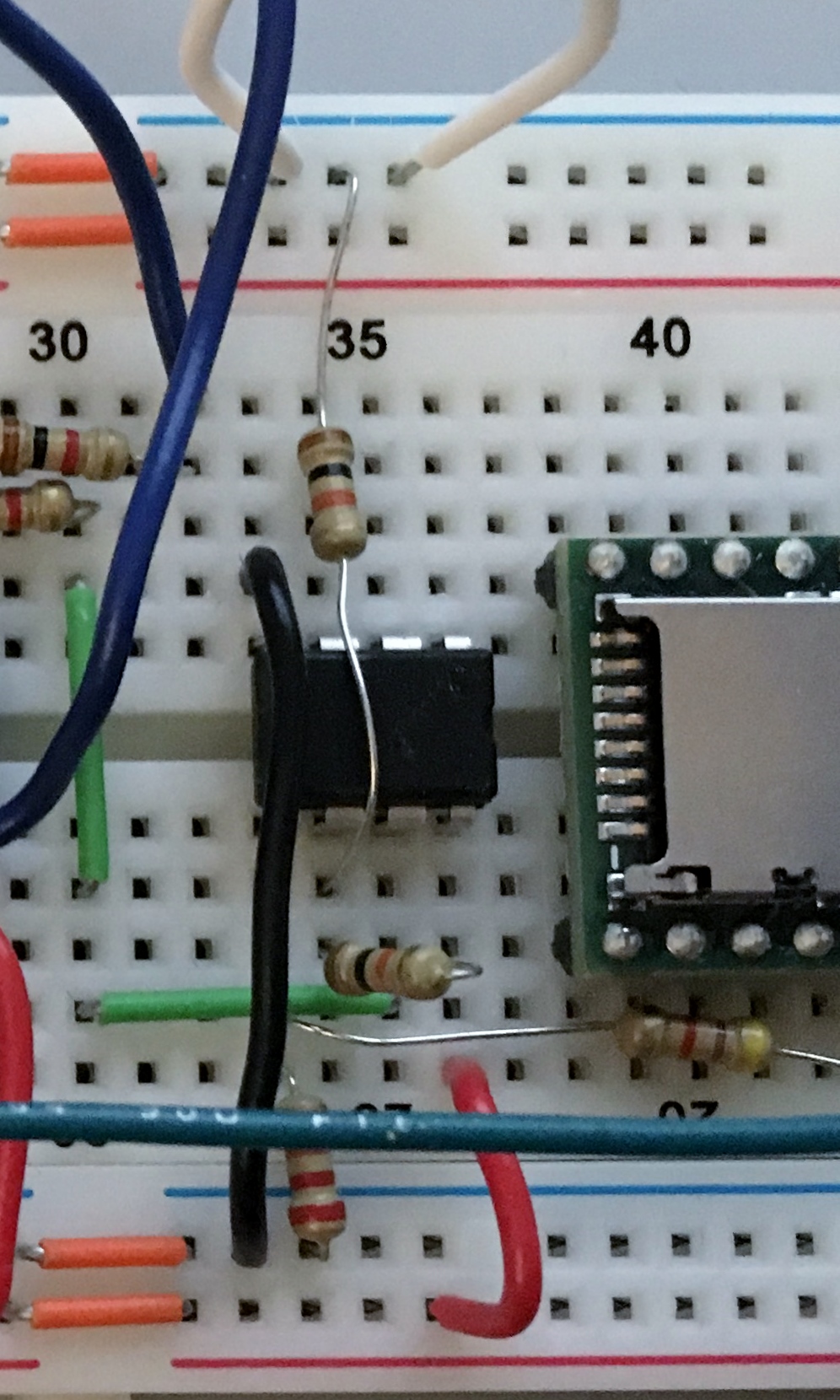

Supplement: Extended Data 1 [file enu005172417so1.zip › Extended_data/2_Assembling_the_system/Higher_quality_images/12_differential_amplifier2.JPG]

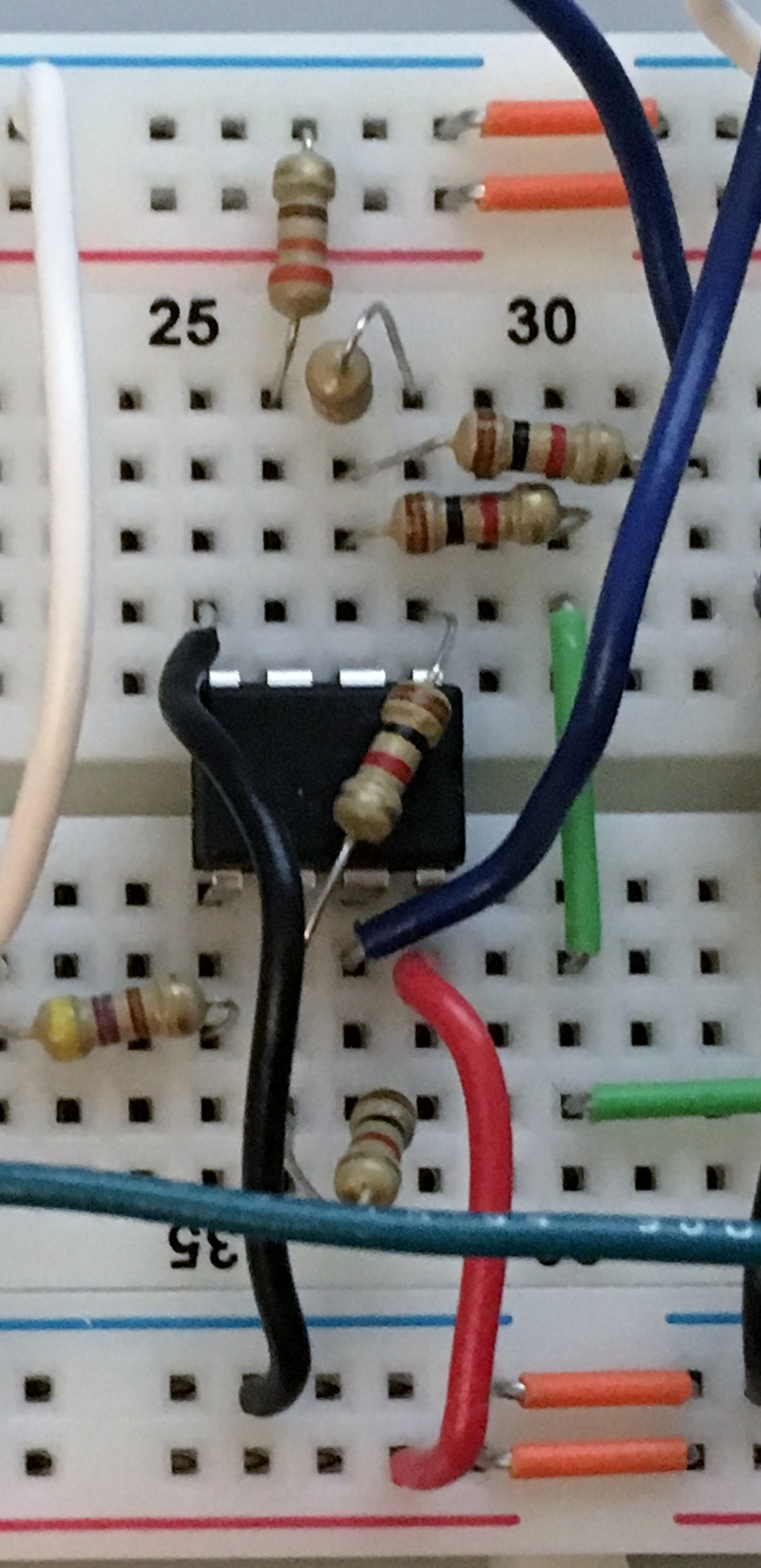

Supplement: Extended Data 1 [file enu005172417so1.zip › Extended_data/2_Assembling_the_system/Higher_quality_images/13_summing_circuit.JPG]

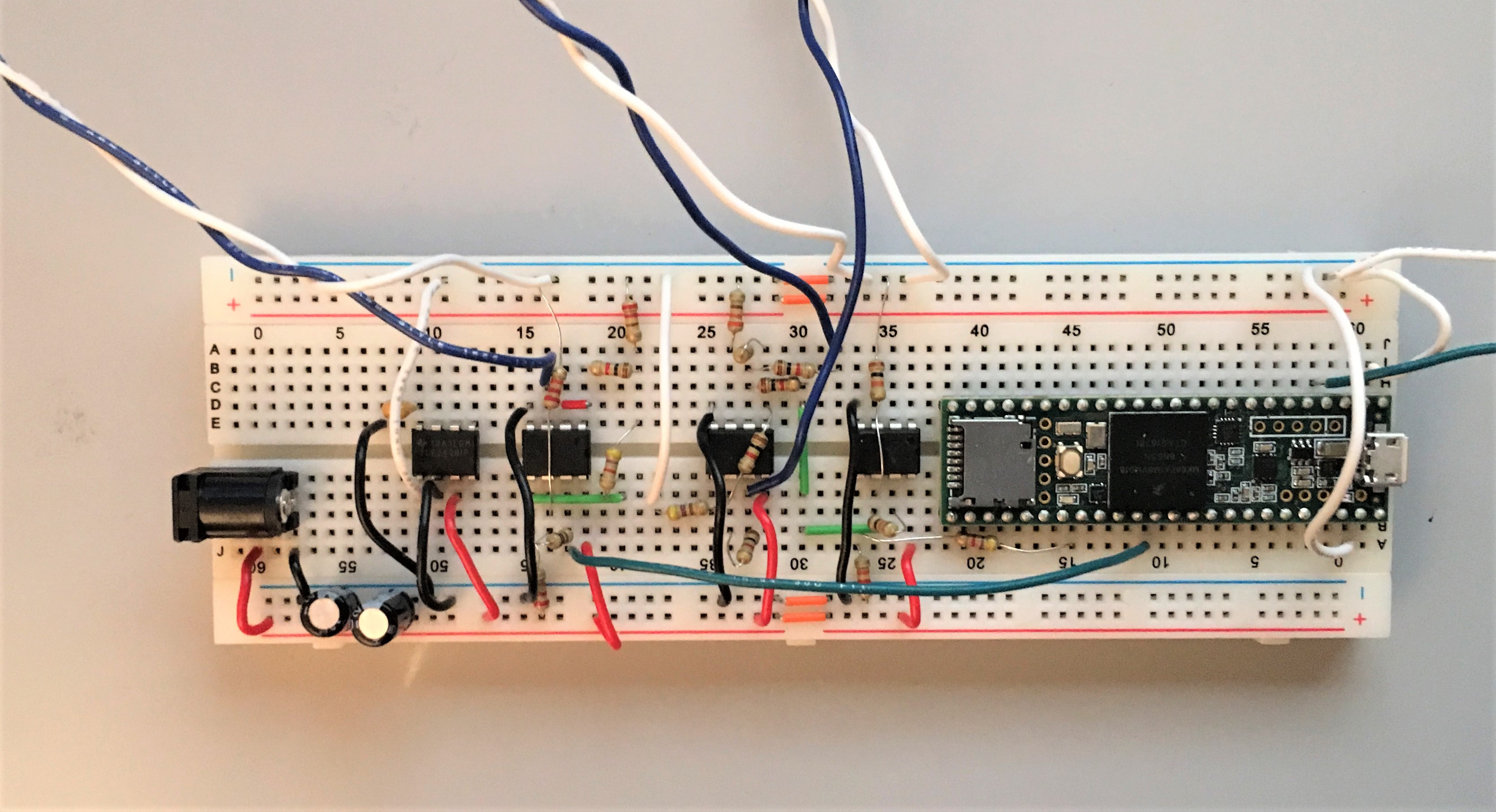

Supplement: Extended Data 1 [file enu005172417so1.zip › Extended_data/2_Assembling_the_system/Higher_quality_images/14_full_ciruit.JPG]

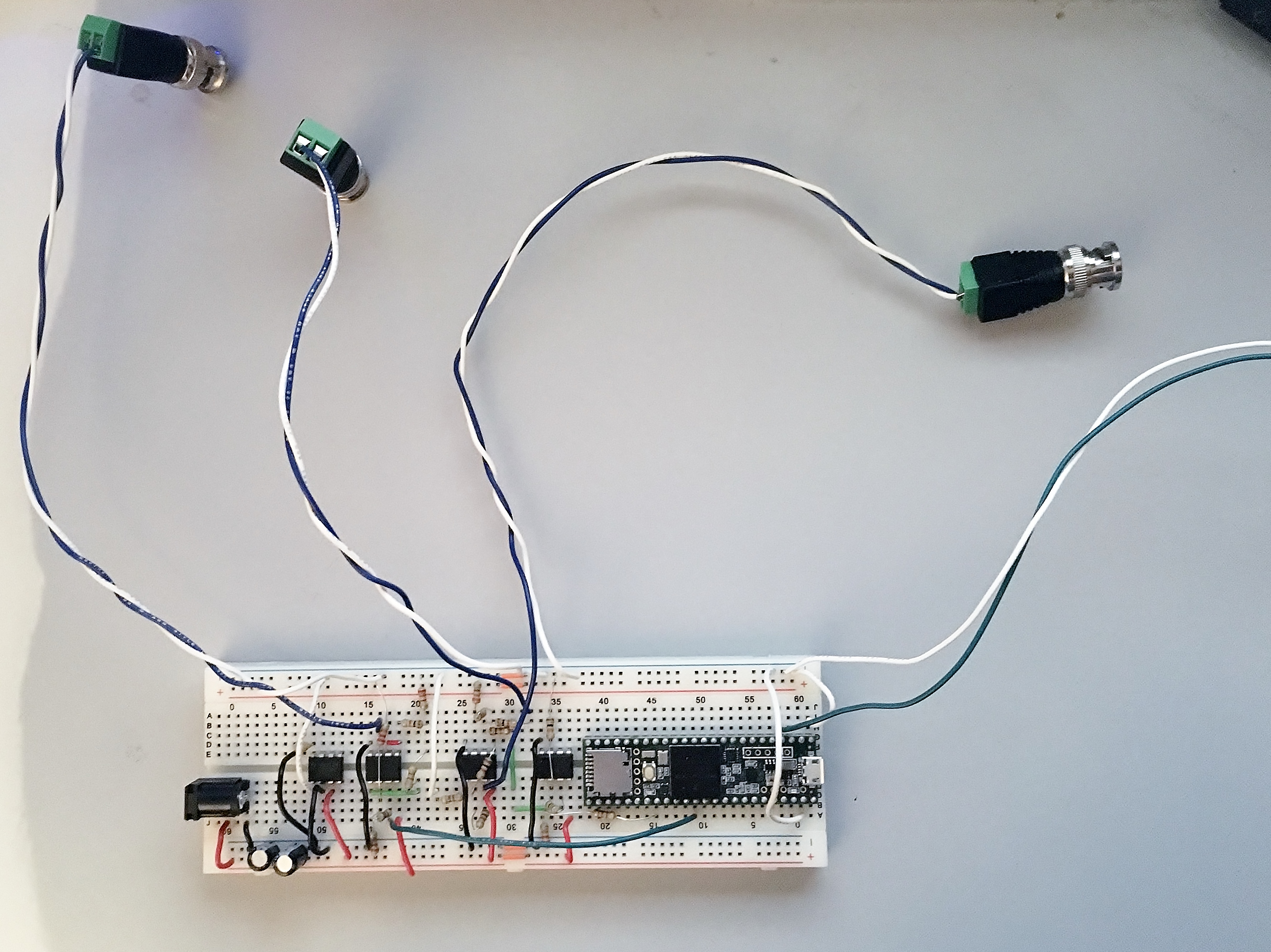

Supplement: Extended Data 1 [file enu005172417so1.zip › Extended_data/2_Assembling_the_system/Higher_quality_images/15_full_circuit.JPG]

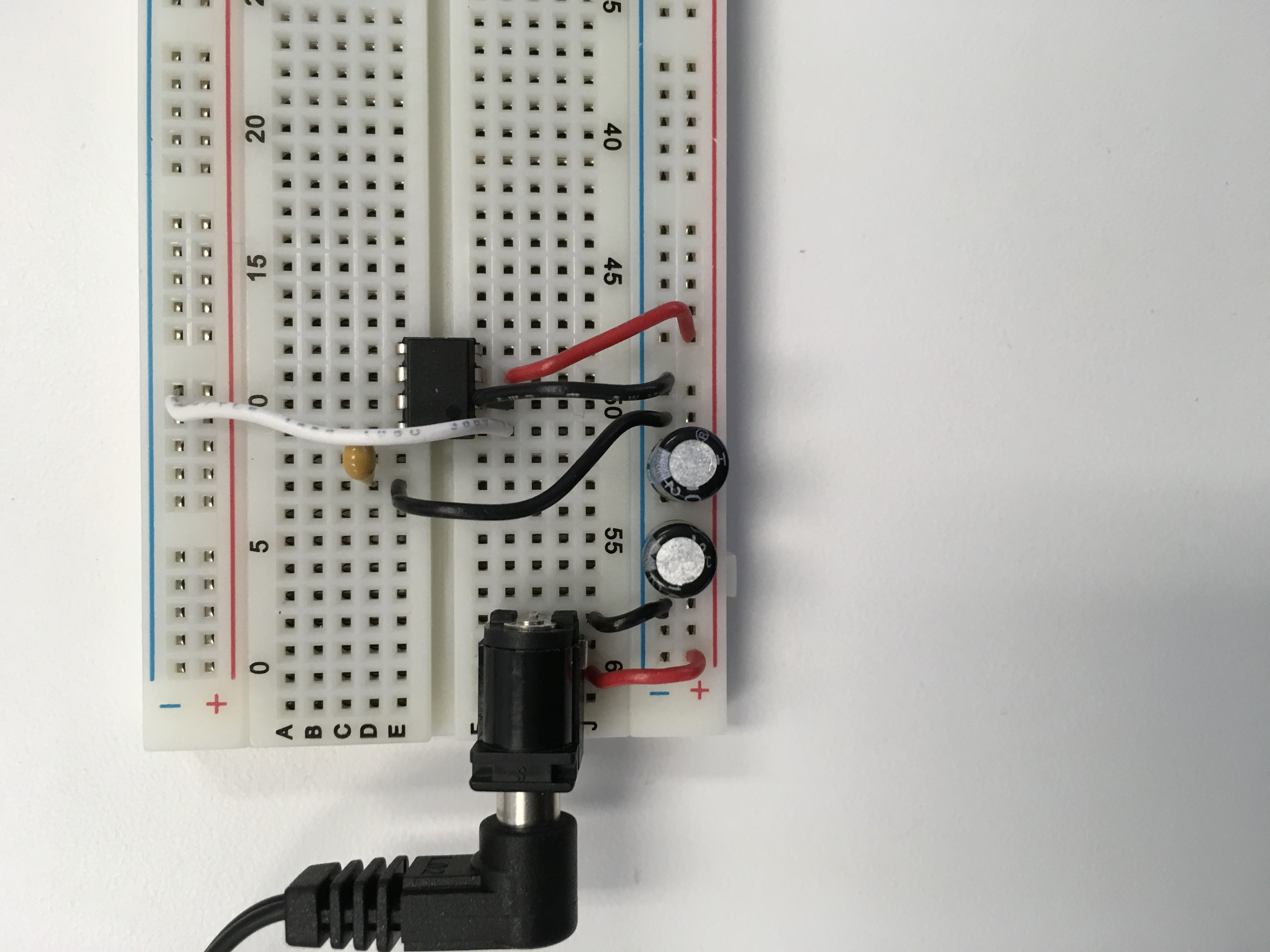

Supplement: Extended Data 1 [file enu005172417so1.zip › Extended_data/2_Assembling_the_system/Higher_quality_images/2_power_supply.JPG]

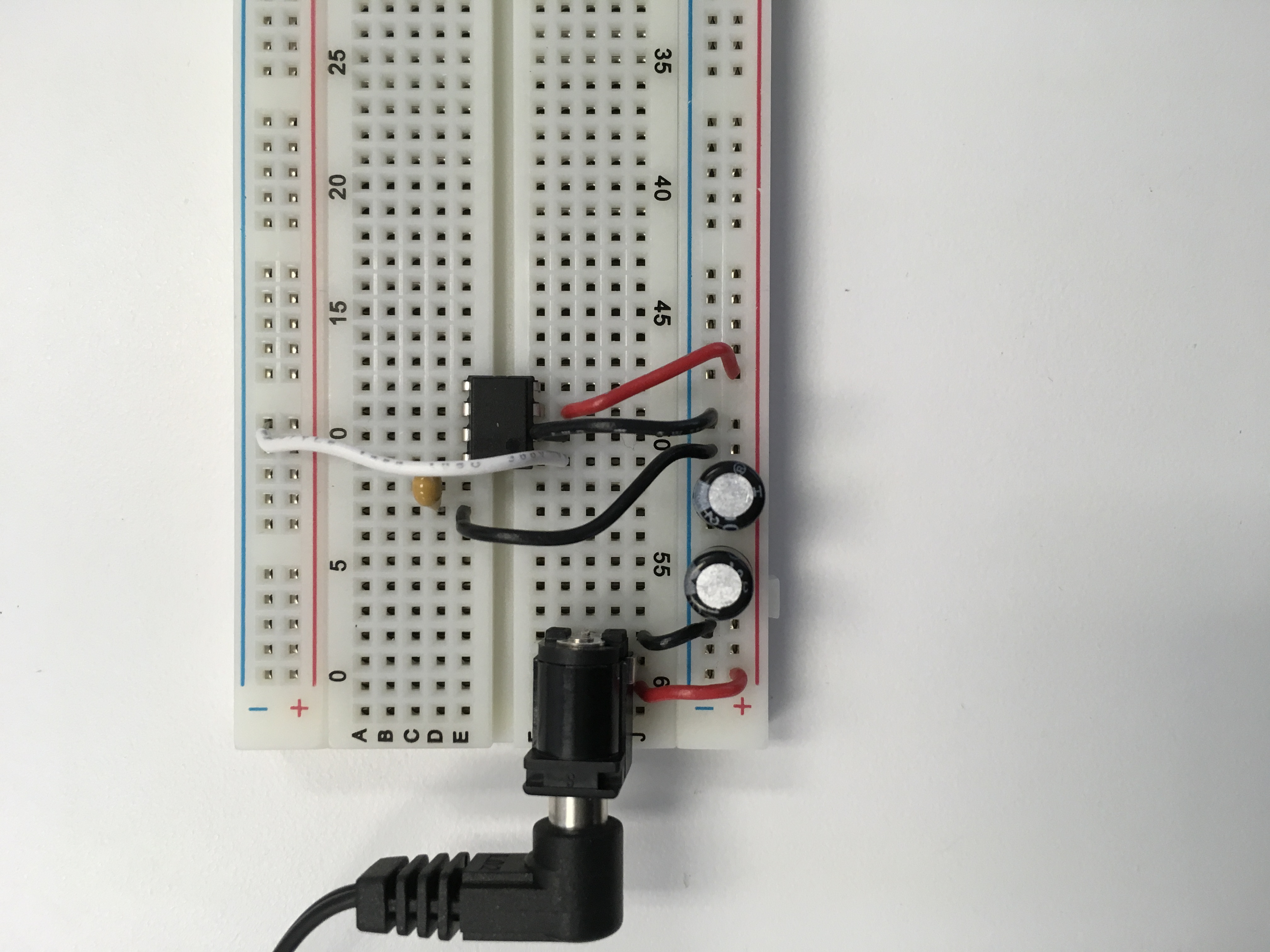

Supplement: Extended Data 1 [file enu005172417so1.zip › Extended_data/2_Assembling_the_system/Higher_quality_images/3_power_supply.JPG]

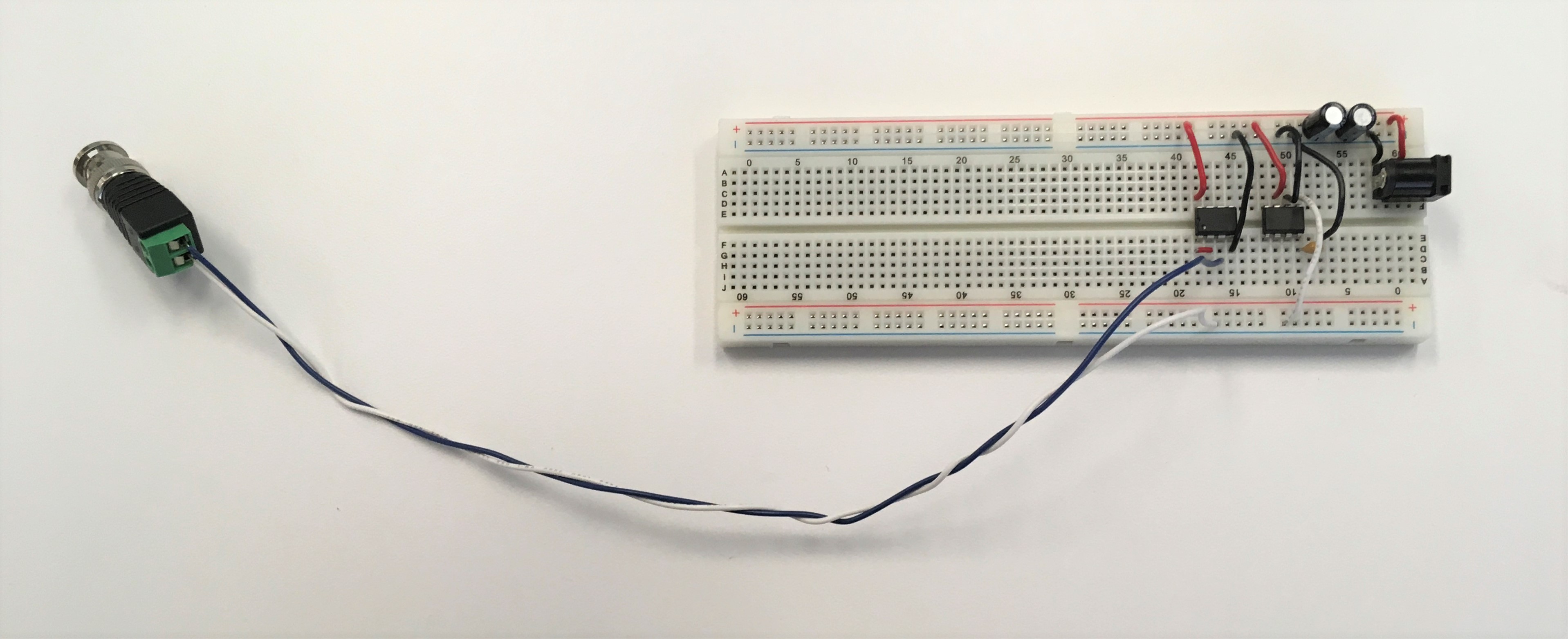

Supplement: Extended Data 1 [file enu005172417so1.zip › Extended_data/2_Assembling_the_system/Higher_quality_images/4_follower_full_view.JPG]

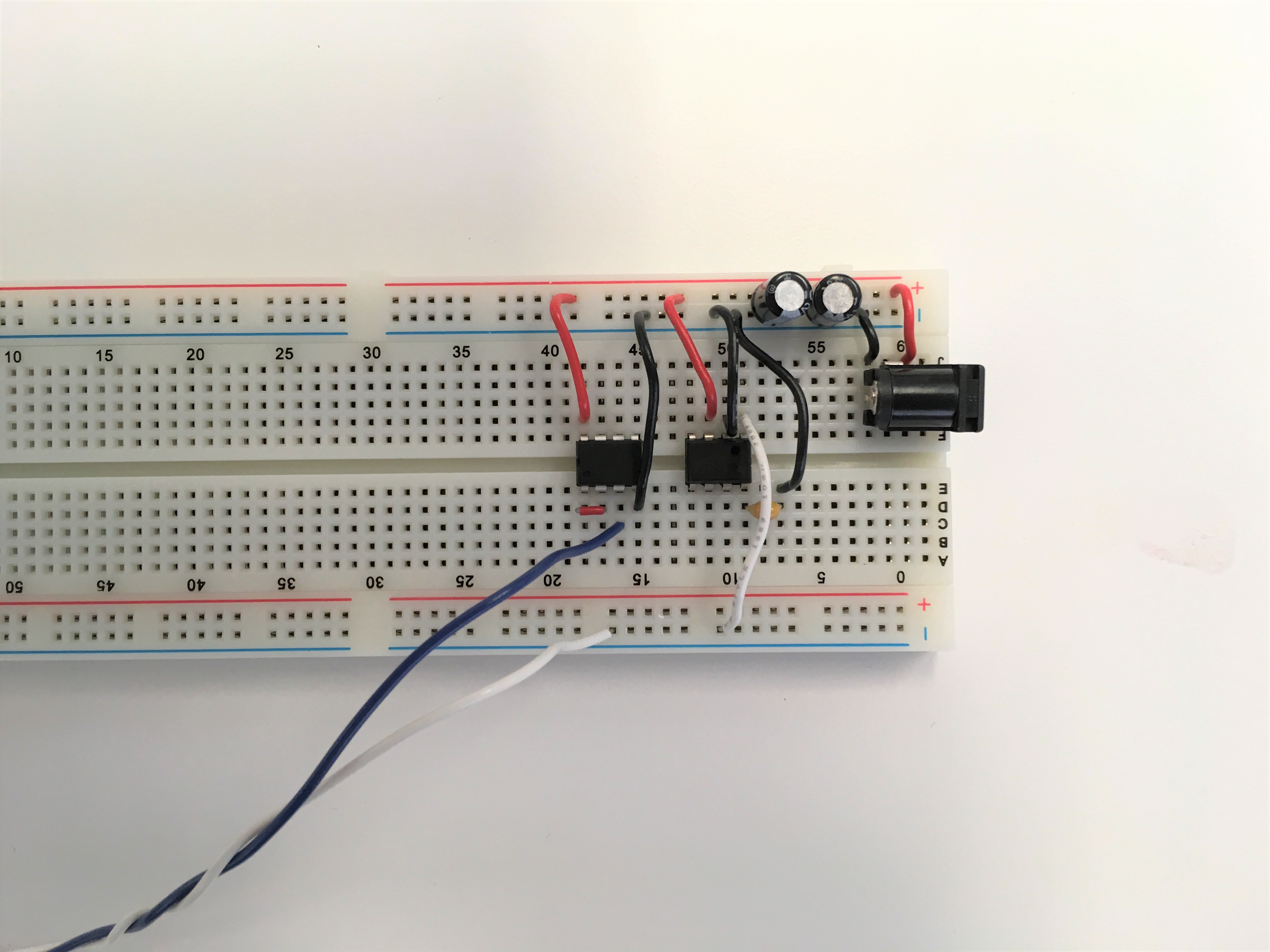

Supplement: Extended Data 1 [file enu005172417so1.zip › Extended_data/2_Assembling_the_system/Higher_quality_images/5_follower.JPG]

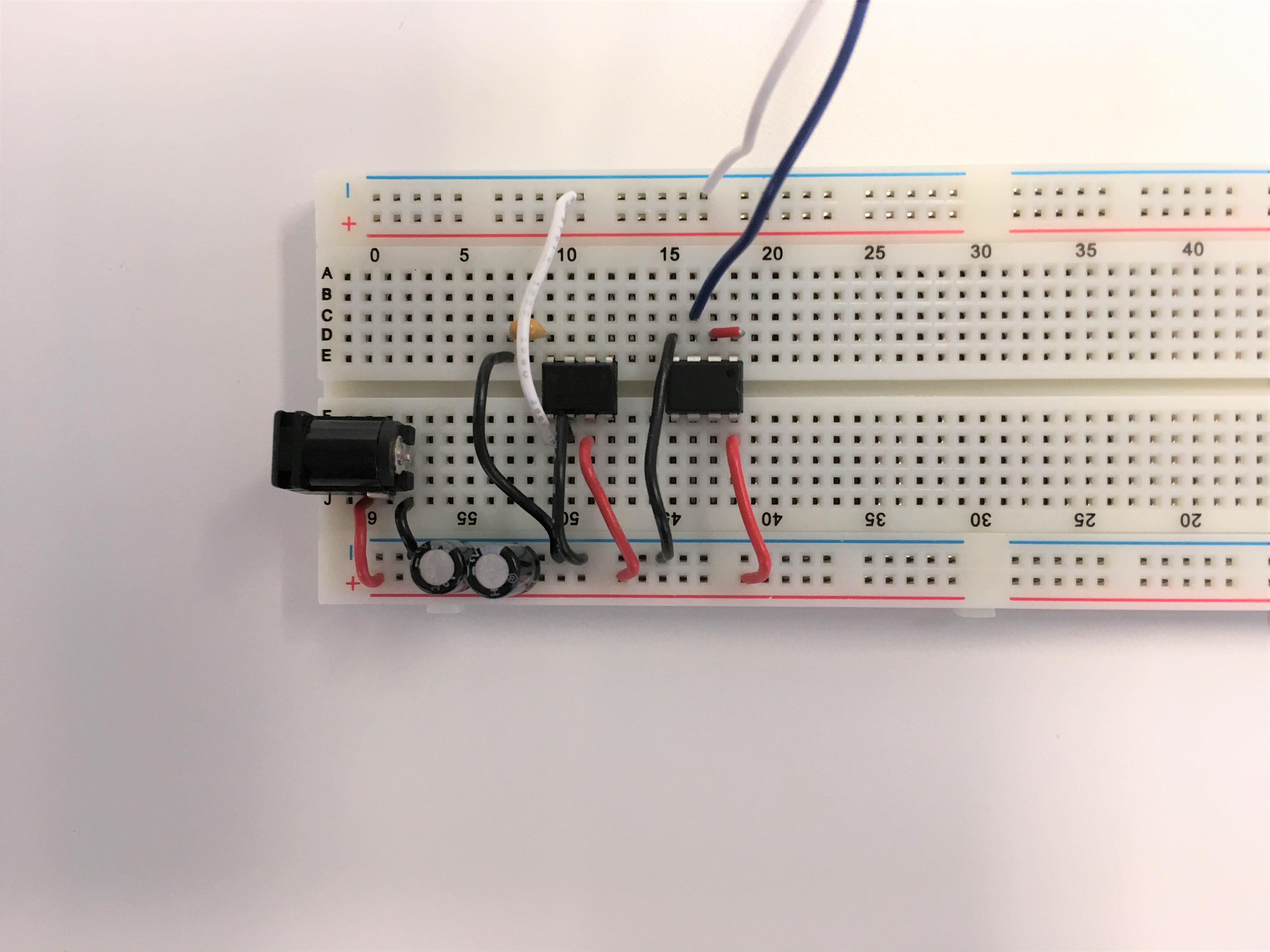

Supplement: Extended Data 1 [file enu005172417so1.zip › Extended_data/2_Assembling_the_system/Higher_quality_images/6_follower.JPG]

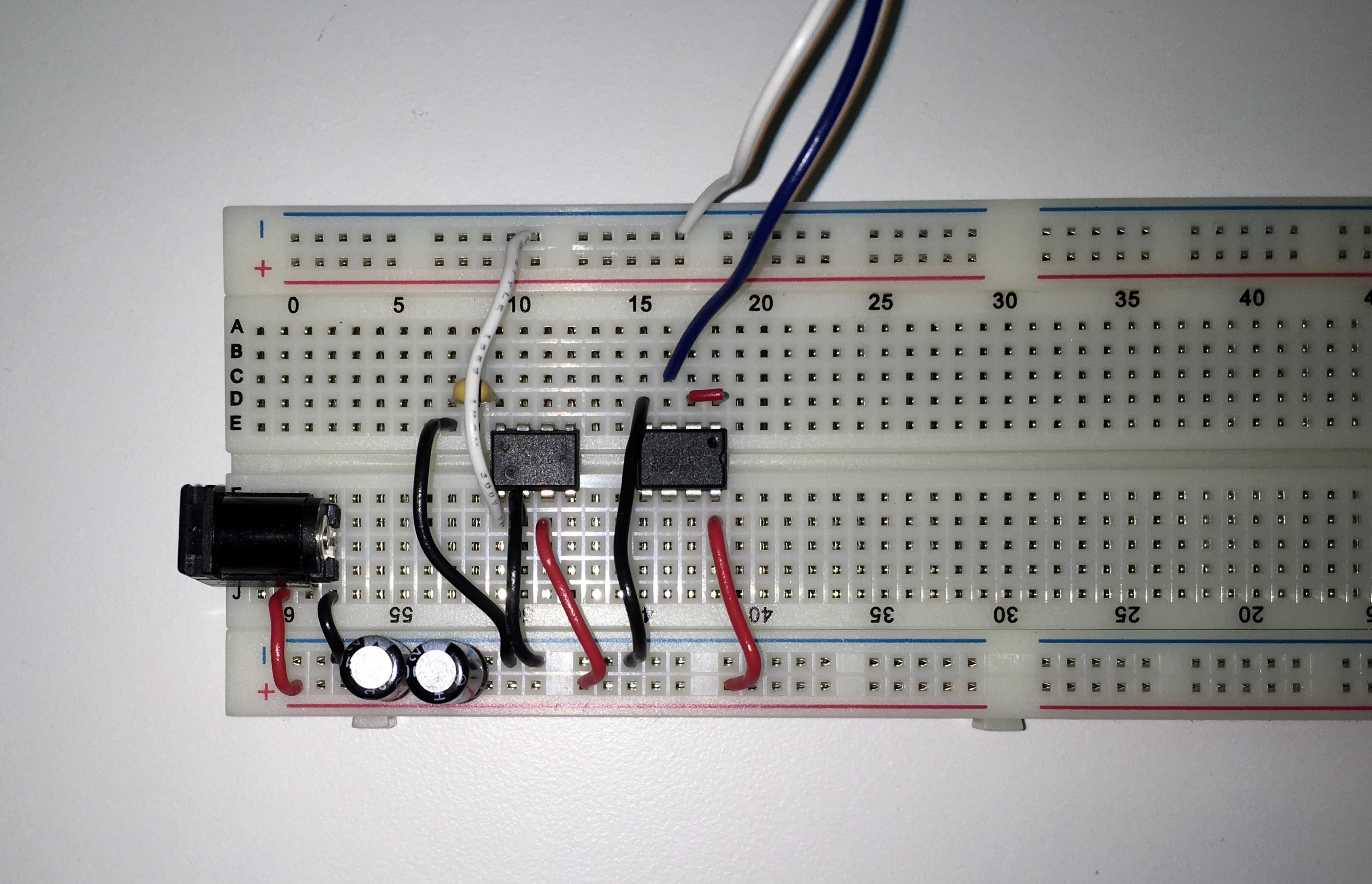

Supplement: Extended Data 1 [file enu005172417so1.zip › Extended_data/2_Assembling_the_system/Higher_quality_images/7_follower.JPG]

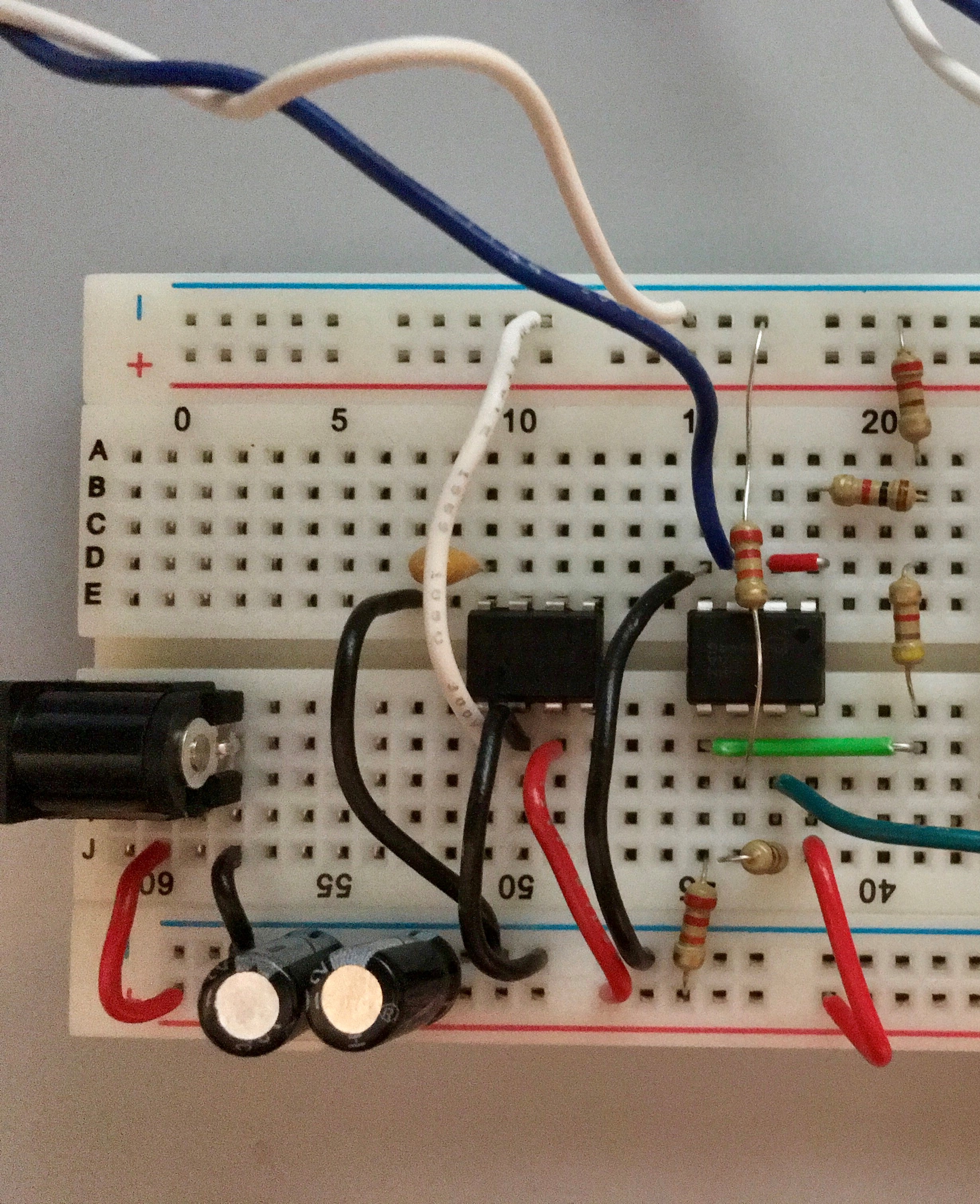

Supplement: Extended Data 1 [file enu005172417so1.zip › Extended_data/2_Assembling_the_system/Higher_quality_images/8_voltage_divider_and_differential_amplifier1.jpg]

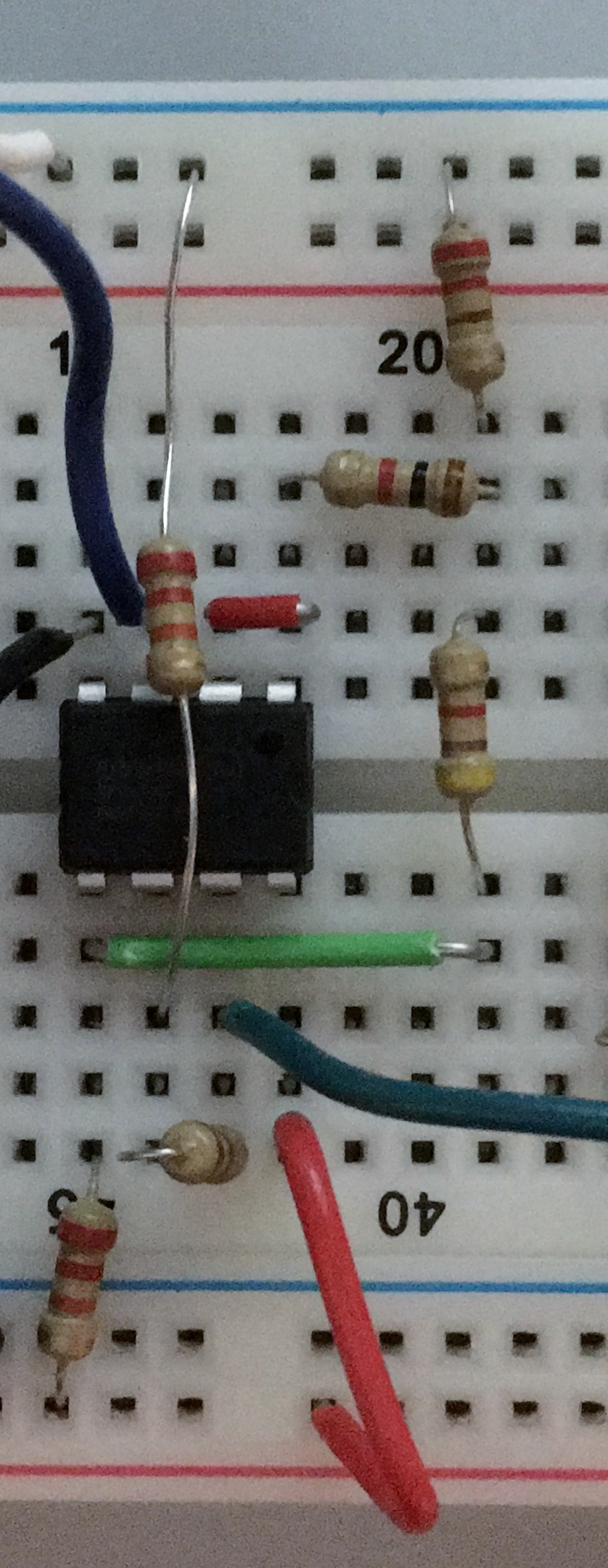

Supplement: Extended Data 1 [file enu005172417so1.zip › Extended_data/2_Assembling_the_system/Higher_quality_images/9_voltage_divider_and_differential_amplifier1.JPG]
